# Structure-Guided Design and Synthesis of a Pyridazinone Series of *Trypanosoma cruzi* Proteasome Inhibitors

**DOI:** 10.1021/acs.jmedchem.3c00582

**Published:** 2023-07-28

**Authors:** Michael G. Thomas, Kate McGonagle, Paul Rowland, David A. Robinson, Peter G. Dodd, Isabel Camino-Díaz, Lorna Campbell, Juan Cantizani, Pablo Castañeda, Daniel Conn, Peter D. Craggs, Darren Edwards, Liam Ferguson, Andrew Fosberry, Laura Frame, Panchali Goswami, Xiao Hu, Justyna Korczynska, Lorna MacLean, Julio Martin, Nicole Mutter, Maria Osuna-Cabello, Christy Paterson, Imanol Peña, Erika G. Pinto, Caterina Pont, Jennifer Riley, Yoko Shishikura, Frederick R. C. Simeons, Laste Stojanovski, John Thomas, Karolina Wrobel, Robert J. Young, Filip Zmuda, Fabio Zuccotto, Kevin D. Read, Ian H. Gilbert, Maria Marco, Timothy J. Miles, Pilar Manzano, Manu De Rycker

**Affiliations:** † Drug Discovery Unit, University of Dundee, School of Life Sciences, Dow Street, Dundee, U.K., DD1 5EH; ‡ GlaxoSmithKline, Chemistry, Medicines Research Centre, Gunnels Wood Road, Stevenage, U.K., SG1 2NY; § GlaxoSmithKline, Discovery DMPK, IVIVT, Severo Ochoa 2, PTM, Tres Cantos, Madrid ES 28760, Spain; ∥ GlaxoSmithKline, Global Health R&D, Severo Ochoa 2, PTM, Tres Cantos, Madrid ES 28760, Spain; ⊥ Blue Burgundy Ltd, Ampthill, Bedfordshire, U.K., MK45 2AD

## Abstract

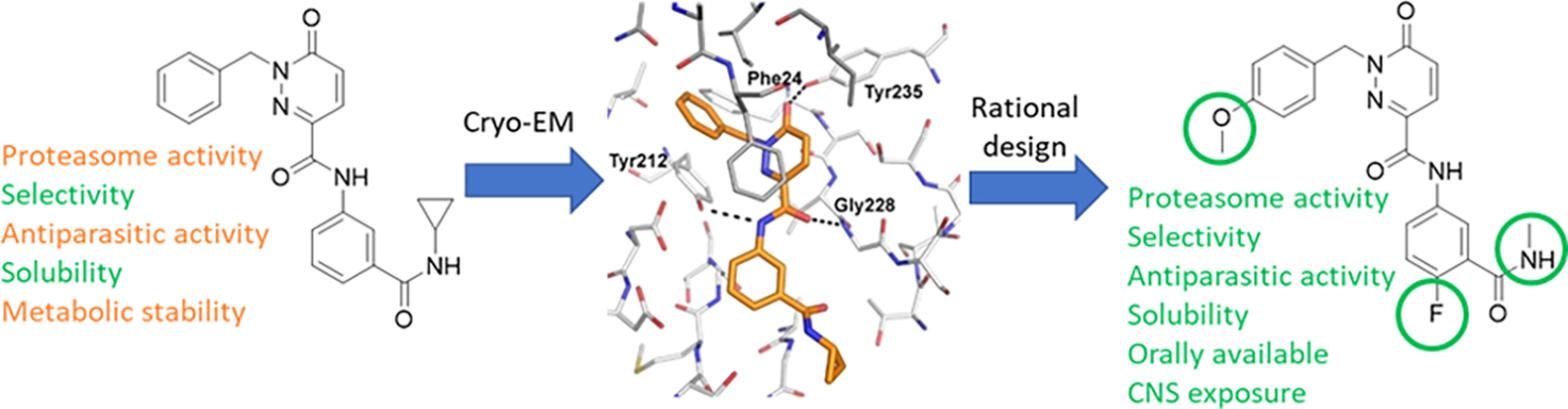

There is an urgent need for new treatments for Chagas disease, a parasitic infection which mostly impacts South and Central America. We previously reported on the discovery of GSK3494245/DDD01305143, a preclinical candidate for visceral leishmaniasis which acted through inhibition of the *Leishmania* proteasome. A related analogue, active against *Trypanosoma cruzi*, showed suboptimal efficacy in an animal model of Chagas disease, so alternative proteasome inhibitors were investigated. Screening a library of phenotypically active analogues against the *T. cruzi* proteasome identified an active, selective pyridazinone, the development of which is described herein. We obtained a cryo-EM co-structure of proteasome and a key inhibitor and used this to drive optimization of the compounds. Alongside this, optimization of the absorption, distribution, metabolism, and excretion (ADME) properties afforded a suitable compound for mouse efficacy studies. The outcome of these studies is discussed, alongside future plans to further understand the series and its potential to deliver a new treatment for Chagas disease.

## Introduction

The kinetoplastids, a group of related parasites, are responsible for diseases which cause an enormous health and economic burden on countries in tropical regions of the world.^1^ One of these, Chagas disease, which is caused by infection with *Trypanosoma cruzi* (*T. cruzi*), is mainly found in South America, Central America, and Mexico, with estimates suggesting there are 6–7 million infected people worldwide, resulting in around 10,000–14,000 deaths per annum.^2−5^ Chagas disease is primarily transmitted *via* an insect vector, the triatomines (kissing or vampire bugs), although other routes of transmission do occur. The disease passes through a series of distinct phases: after the initial acute infection, patients enter the indeterminate phase, which is characterized by low levels of parasites and lack of overt symptoms. A subset of patients develops symptomatic disease with serious health conditions such as cardiomyopathy and digestive disorders.

There are currently only two approved treatments for Chagas disease, benznidazole (BNZ) and nifurtimox,^6,7^ whose use is hampered by issues including limited efficacy and side effects which can lead to early treatment discontinuation. Recent clinical trials, such as the BENDITA trial (NCT03378661),^8^ suggest improved dosing regimens for BNZ might be beneficial, but the pipeline of alternative treatments is still very limited, incorporating fexinidazole and the oxaborole compounds DNDi-6148^9,10^ and AN15368.^11^

**Figure 1:**
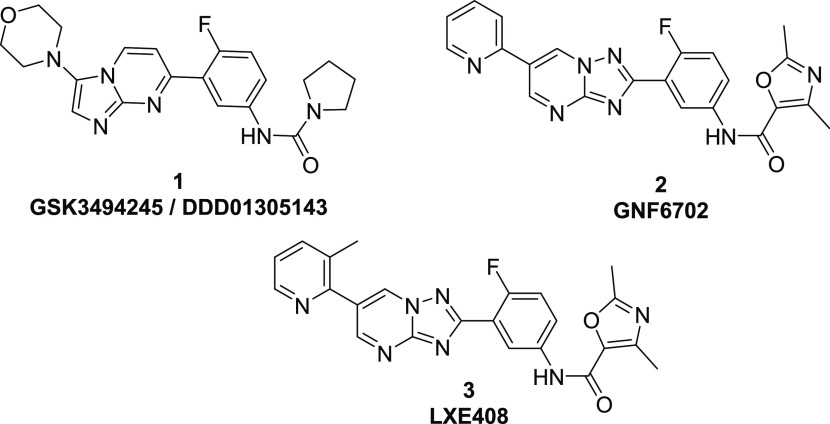
Structures of GSK3494245/DDD01305143 (**1**), GNF6702 (**2**) and LXE408 (**3**).

There is therefore an urgent need for new treatments, particularly ones with differentiated modes of action compared to those already in the clinic and those which have been shown to be ineffective (such as CYP51 inhibitors).^12−15^ A potential start-point for new therapies would be compound series that target the related parasites *Trypanosoma brucei* and *Leishmania*, whose mechanism of action has the potential to be applied to Chagas disease. From a collaboration between the Drug Discovery Unit at the University of Dundee and GSK’s Global Health Research Centre in Tres Cantos, we recently disclosed the clinical candidate GSK3494245/DDD01305143 **1** for visceral leishmaniasis (VL) (Figure 1).^16,17^ While the series was developed phenotypically, it was subsequently demonstrated to act through inhibition of the β5 (chymotrypsin) proteolytic activity of the parasite proteasome, with a cryo-EM structure of the proteasome–inhibitor complex being generated. The proteasome is a multi-subunit protease that is conserved between higher eukaryotes and protozoan parasites and is a key component of the ubiquitin-proteasome system which is essential for the maintenance of intracellular protein homeostasis.^18^ The core particle of the proteasome consists of 28 subunits organized into four rings of seven subunits and harbors the three proteolytic activities: caspase-like (β1 subunit, preferential cleavage after acidic amino acids), trypsin-like (β2 subunit, preferential cleavage C-terminal to positively charged amino acids), and chymotrypsin-like (β5 subunit, preferential cleavage after hydrophobic amino acids).^19,20^ Due to its essential role in protein homeostasis, the proteasome is expected to be a good drug target across protozoan infectious diseases.^21−23^ Specifically for *T. cruzi*, *in vivo* chemical validation of the proteasome as a suitable drug target was obtained with the Novartis compounds GNF6702 **2**(24) and LXE408 **3**,^25^ two compounds related to **1**. This series has pan-kinetoplastid activity and **3** is currently progressing to clinical trials for VL (Figure 1).

Herein, we disclose our efforts to repurpose the series, which led to **1** into Chagas disease, and a subsequent screening campaign to identify novel chemical start-points. We report the use of cryo-EM to aid design, the development of compounds suitable for dosing in a chronic mouse model of Chagas disease, and the results of these efficacy studies.

## Results and Discussion

### Repurposing of Antileishmanial Proteasome Inhibitors for Chagas Disease

**Figure 2:**
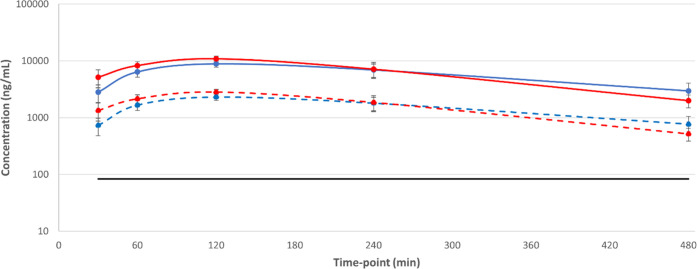
Total and free blood concentrations for compound **4** from a mouse efficacy study for visceral leishmaniasis after initial doses on days 1 and 5, with *T. cruzi* EC_99_ plotted to demonstrate potential coverage in *in vivo* models of Chagas disease. Solid blue line = day 1 total concentration, solid red line = day 5 total concentration; dashed blue line = day 1 free concentration, dashed red line = day 5 free concentration; solid black line = *T. cruzi* EC_99_. Dosing was initiated 7 days post-infection.

From the previously reported series of *Leishmania* proteasome inhibitors,^16,17^ one of the compounds, **4**, was profiled for its potential repurposing for Chagas disease. As shown in Table 1, **4** was a potent, selective inhibitor of the *T. cruzi* proteasome,^26^ and this translated into potency in the intracellular *T. cruzi* assay^27^ with a pEC_50_ of 6.9 and no activity against the host VERO cells. **4** was tested in an *in vitro* washout assay,^28^ and we were encouraged to see that after 8 days of treatment **4**, at 50-fold EC_50_, performed better than BNZ, with parasites relapsing on day 34, compared to day 21 for BNZ. **4** had previously been run in a mouse efficacy model of VL, dosed at 50 mg/kg and plotting exposure obtained from this study against the *T. cruzi* EC_99_ (Figure 2) showed that exposure in blood was well above the *T. cruzi* EC_99_ for >8 h, even when correcting for plasma protein binding. Although these were VL-infected mice, we have not generally seen marked differences in drug exposure between uninfected and *Leishmania*/*T. cruzi*-infected mice (unpublished data).

**Table 1:**
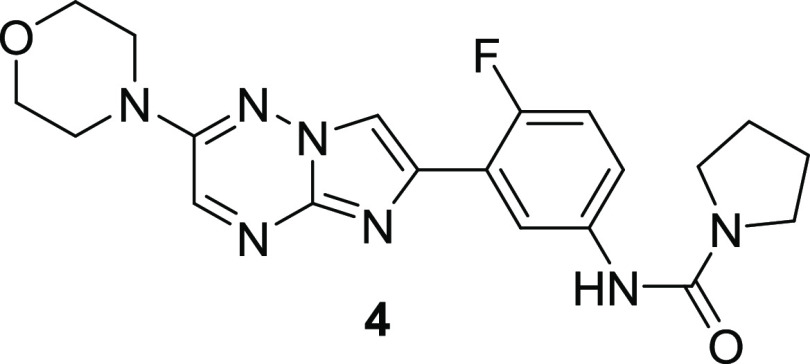
*In Vitro* Profile of **4**

*T. cruzi* prot pIC_50_^a^	7.3
Hs prot pIC_50_^a^	<4
*T. cruzi* pEC_50_^b^	6.9
VERO pEC_50_^b^	<4.3
aqueous solubility (μM)^c^	59
FaSSIF solubility (μM)^d^	32
mouse CL_*i*_ (mL/min/g)^e^	<0.5
Papp(A–B) (nm s^–1^)/ER^f^	413/22

a Inhibition of *T. cruzi* and human proteasome, data from at least three independent replicates, standard deviations ≤0.2. All data are provided in the Supporting Information.

b *T. cruzi* pEC_50_: potency against intracellular *T. cruzi* amastigotes and host VERO cells, data from at least three independent replicates, standard deviation ≤0.3. All data are provided in the Supporting Information.

c Kinetic solubility measured by chemiluminescent nitrogen detection (CLND).^29^

d FaSSIF solubility is the fasted state simulated intestinal fluid solubility.

e Cl_*i*_ is the mouse liver microsomal intrinsic clearance; scaling factor used is 52.5 mg of microsomal protein/g liver.

f Permeability in MDR1-transfected MDCK cells in the presence of a Pgp inhibitor (GF120918)/efflux ratio.

Based on its *in vitro* profile, together with the pharmacokinetic profile obtained from the VL efficacy study, **4** was progressed into a chronic mouse efficacy model of Chagas disease.^30^ In this case, after treating the mice for 5 days at 50 mg/kg bid, there was no visible effect on parasite levels even though exposure was consistent with the VL efficacy study (BNZ dosed at 100 mg/kg reduced parasite levels below detectable limits).

**Figure 3:**
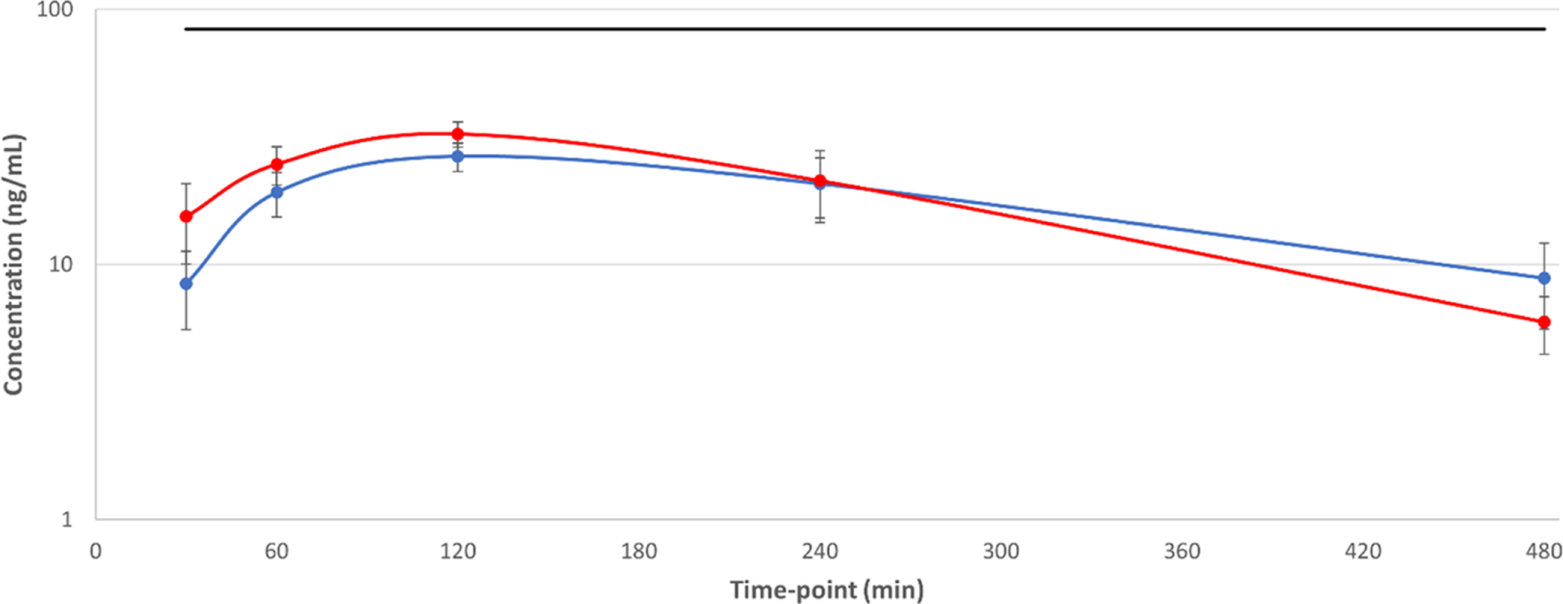
Predicted free exposure of **4** in the CNS, based on the blood–brain ratio of 0.07, brain tissue free fraction of 4.3%, and the blood exposure from the chronic mouse efficacy model of Chagas disease. Solid blue line = day 1 predicted free brain concentration; solid red line = day 5 predicted free brain concentration; solid black line = *T. cruzi* EC_99_.

Based on the promising results in the washout study, we were keen to understand the lack of effects in the *in vivo* study, where differences between parasite and compound distribution could be an important factor, with the parasites being widely distributed to sites including adipose tissue, ovaries, and the CNS.^31,32^ Monitoring both parasite and compound distribution *in vivo* would be challenging so we elected to utilize CNS exposure as a surrogate for wider compound distribution to the other conserved sites where the parasite resides. The brain–blood ratio for **4** was shown to be 0.07, suggesting limited CNS penetration, likely due to **4** being a strong Pgp substrate with an efflux ratio (ER) of 22 in an MDR1-MDCK assay. Indeed, predicted brain exposure of **4** was well below *T. cruzi* EC_99_ (Figure 3).

A large number of analogues of **4** were screened in the MDR1-MDCK assay, but no potent, low-efflux compounds were identified (data not shown). Because of this, we decided to utilize our knowledge of the mechanism of action of the series and our capability to generate cryo-EM structures to initiate a hit-discovery campaign against the *T. cruzi* proteasome.

### Identification of *T. cruzi* Proteasome Inhibitors

Approximately 26,000 compounds from the GSK collection with activity against *T. cruzi*(33) were screened in a *T. cruzi* proteasome assay with actives also tested in a human proteasome assay^26^ in order to identify hits that were potent against the parasite proteasome and selective against the human proteasome, as well as having antiparasitic activity. Although there was no guarantee that intracellular activity was solely driven by proteasome inhibition, this could be monitored as a hit series was developed. Any hits would also be screened in the MDR1-MDCK assay, with the aim of identifying a hit with markedly reduced Pgp substrate interaction compared to **4**.

This screening cascade led to the identification of **5**, a functionalized pyridazinone with our desired potency/selectivity profile and reasonable LE/LLE (0.29, 2.5), albeit with an ∼10-fold drop-off in potency from *T. cruzi* proteasome inhibition to antiparasitic activity. Further profiling showed **5** to have good aqueous solubility, reasonable lipophilicity, and high permeability in the MDR1-MCDK assay (Table 2). The ER was 6, which we considered a reasonable start-point for optimization, especially compared to **4** with an ER of 22. Ideally, we aimed to reduce the ER to <3 during the medicinal chemistry program, to maximize our chances of achieving efficacious free drug levels in the CNS and other conserved sites.

**Table 2:**
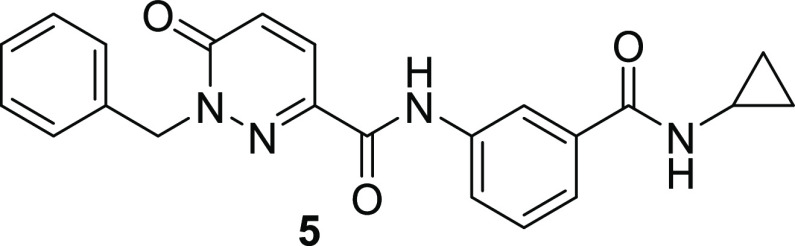
*In Vitro* Profile of **5**

*T. cruzi* prot pIC_50_^a^	6.2
Hs prot pIC_50_^a^	<4
*T. cruzi* pEC_50_^b^	5.0/<4.3
aqueous solubility μM^c^	221
Chrom Log *D*_pH7.4_^d^	3.9
LE/LLE (Prot)^e^	0.29/2.5
Papp(A–B) nm s^–1^/ER^f^	337/6.0

a Inhibition of *T. cruzi* and human proteasome, data from at least three independent replicates, standard deviations ≤0.2 All data are provided in the Supporting Information.

b Potency against intracellular *T. cruzi* amastigotes and host VERO cells, data from at least three independent replicates, standard deviation ≤0.25. All data are provided in the Supporting Information.

c Kinetic aqueous solubility measured by a UHPLC system equipped with a UV/visible and single-quadrupole mass spectrometer.

d Chrom Log *D*_pH7.4_ = CHI_pH7.4_ × 0.0857 – 2, where CHI is the chromatographic hydrophobicity^34^ index.

e Ligand efficiency/lipophilic ligand efficiency from *T. cruzi* proteasome IC_50_.

f Permeability in MDR1-transfected MDCK cells in the presence of a Pgp inhibitor (GF120918)/efflux ratio.

As **5** showed a suitable profile for hit expansion, we were keen to utilize our cryo-EM platform to generate structural information. Due to challenges with obtaining the *T. cruzi* proteasome cryo-EM structure, we used the closely related *Leishmania tarentolae* proteasome as a surrogate (β5 subunit amino acids are 95% similar and 87% identical).

### Cryo-Electron Microscopy Studies of **5**

The complex of **5** bound to the *L. tarentolae* 20S proteasome was determined by single-particle cryo-EM to 2.6 Å resolution. Clear features in the electron potential map located at the β4/β5 interface allowed the ligand to be modeled successfully (Supporting Figures 1 and 2).

**Figure 4:**
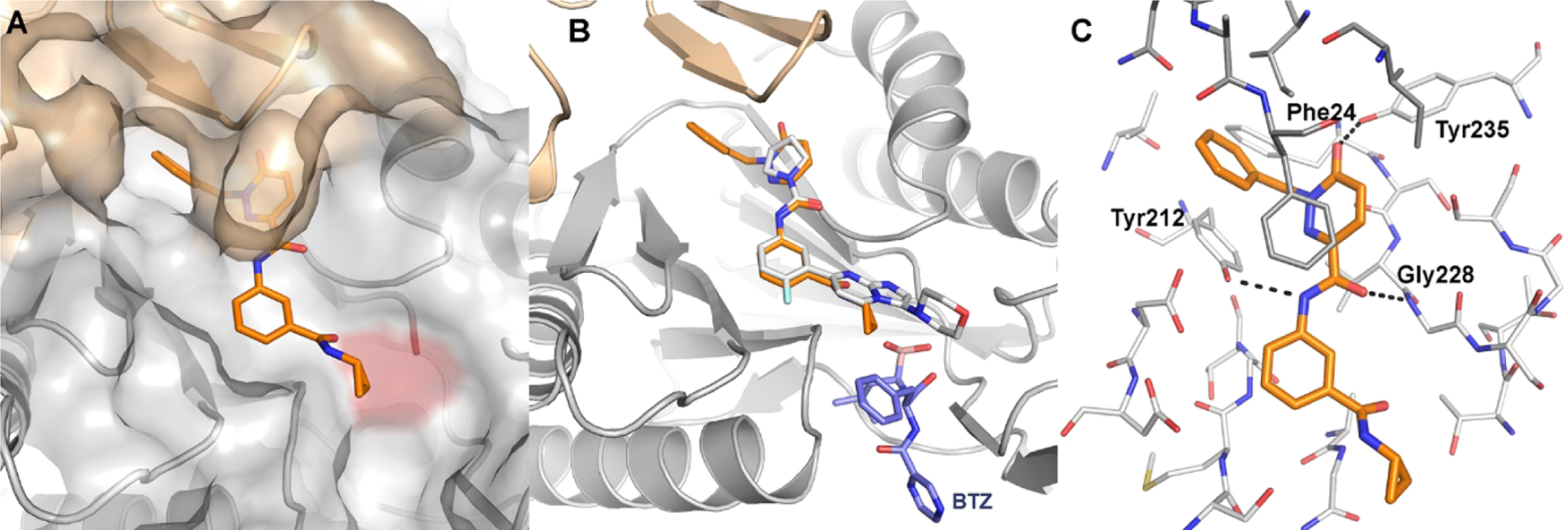
*L. tarentolae* 20S proteasome in complex with **5** (PDB 8OLU). A: Binding mode of **5** (C atoms gold) bound to the β4/β5 interface of the 20S proteasome. β4 subunit is colored brown, and β5 is colored gray with catalytic β5Thr100 highlighted in red. B: Binding mode of **5** (C atoms gold) compared to **1** (C atoms gray) and bortezomib (C atoms blue). C: Binding mode of **5** (C atoms gold) bound to *L. tarentolae* 20S proteasome (C atoms gray). Hydrogen bonds are shown in dotted lines.

Compound **5** is bound in a similar orientation to the previously reported **1**(17) and **3**(25) at the β4/β5 interface close to the catalytic β5Thr100 (Figure 4). Hydrogen-bond interactions are formed between the central amide with the backbone NH of β5Gly228 and the side-chain phenolic hydroxyl of β5Tyr212. Similar to **1**, conformational changes within the β4 subunit occur with respect to the apo *L. tarentolae* 20S proteasome to accommodate the pyridazinone moiety with a hydrogen bond formed between the pyridazinone carbonyl and the side chain of β5Tyr235. Additional conformational changes are observed in the β4 subunit as the chain of Ile29 rotates, allowing the benzyl moiety to occupy a hydrophobic pocket, forming π–π interactions with side chains of β5Tyr212 and β4Phe24.

**Figure 5:**
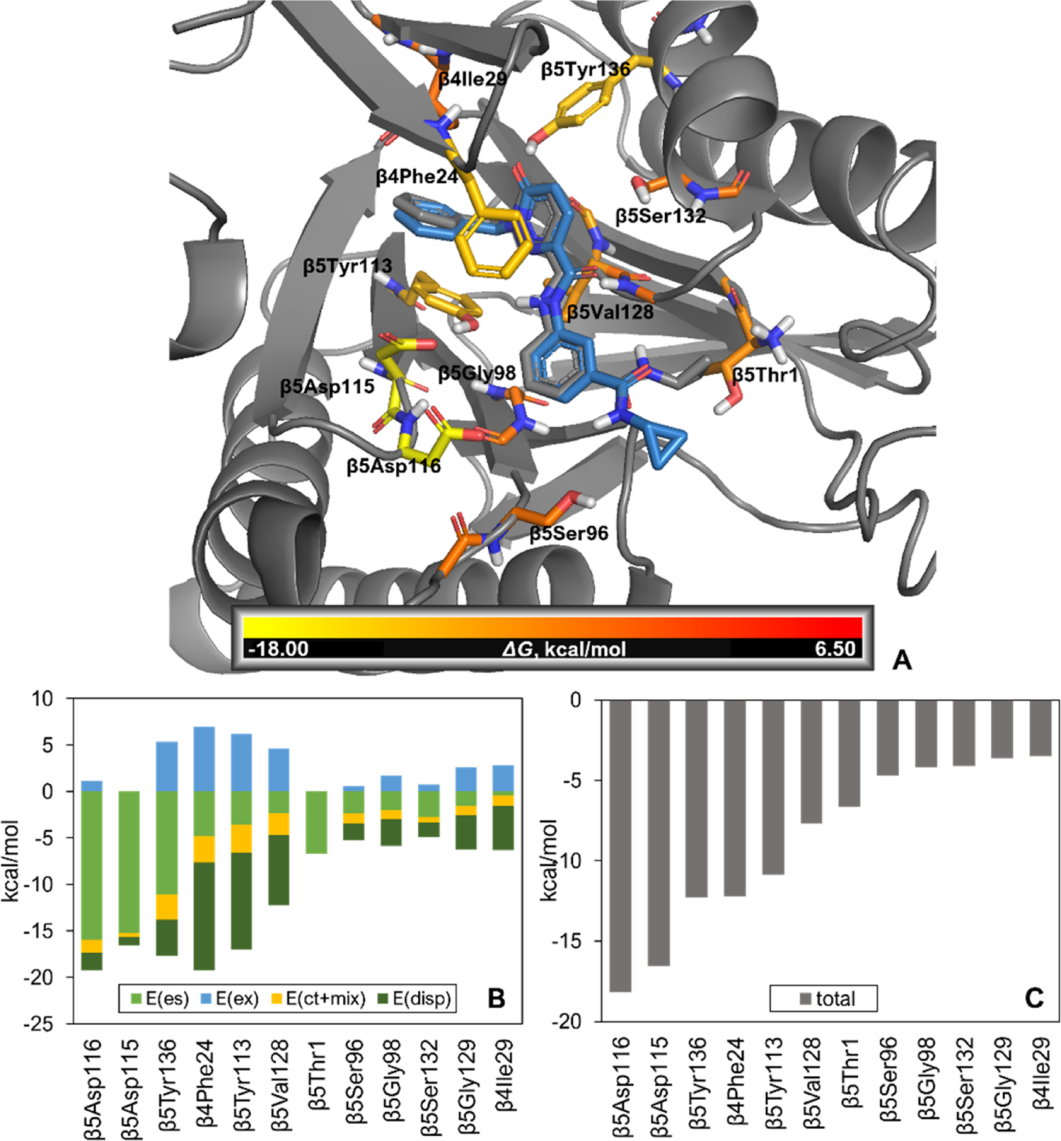
(A) Docked pose of **5** in a *T. cruzi* homology model (C atoms sea blue) overlaid with the *L. tarentolae* 20S proteasome cryo-EM crystal structure (gray, PDB 8OLU). The residues contributing less than −3.0 kcal·mol^–1^ are shown and colored according to calculated MP2-FMO energy contribution. (B, C) MP2-FMO PIEDA analysis of docked pose in *T. cruzi* 20s proteasome homology model. Decomposed (B) and total (C) energetic contributions are shown. Abbreviations for energetic components: E(es): electrostatic, E(ex): exchange repulsion, E(ct+mix): charge transfer, E(disp): dispersion.

To support prospective molecular design, molecules were docked into a homology model of the *T. cruzi* 20S proteasome developed from the complex of **5** bound to the *L. tarentolae* 20S proteasome using the homology modeling tool available in Maestro (Schrödinger suites version 2020-04). Docking of **5** to the *T. cruzi* 20S proteasome homology model replicated the key features of the binding mode defined experimentally for the *L. tarentolae* 20S proteasome, with the central amide, pyridazinone, and benzyl moieties overlapping as well as the protein residues that define the binding site (Figure 5). In order to gain a deeper understanding of the ligand–protein interactions driving the molecular recognition process, we carried out a quantum mechanics (MP2-FMO)^35,36^ analysis of the docked pose of **5** in the *T. cruzi* 20S proteasome homology model (Figure 5B,C). Compared to the previously reported analysis of **1**,^17^ the energy contribution from the N-terminal β5Thr1 is significantly weakened for compound **5**. β5Asp116 (−18.18 kcal·mol^–1^) and β5Asp115 (−16.55 kcal·mol^–1^) became the major contributing residues in terms of MP2-FMO PIEDA estimations. Moreover, residues β5Tyr136 (−12.30 kcal·mol^–1^), β4Phe24 (−12.23 kcal·mol^–1^), and β5Tyr113 (−10.89 kcal·mol^–1^) also show improved binding energy contribution, through either hydrogen bonding to the pyridazinone carbonyl (β5Tyr136) or hydrophobic interactions with the benzyl group (β4Phe24 and β5Tyr113), interactions that were not present in compound **1**.

**Figure 6:**
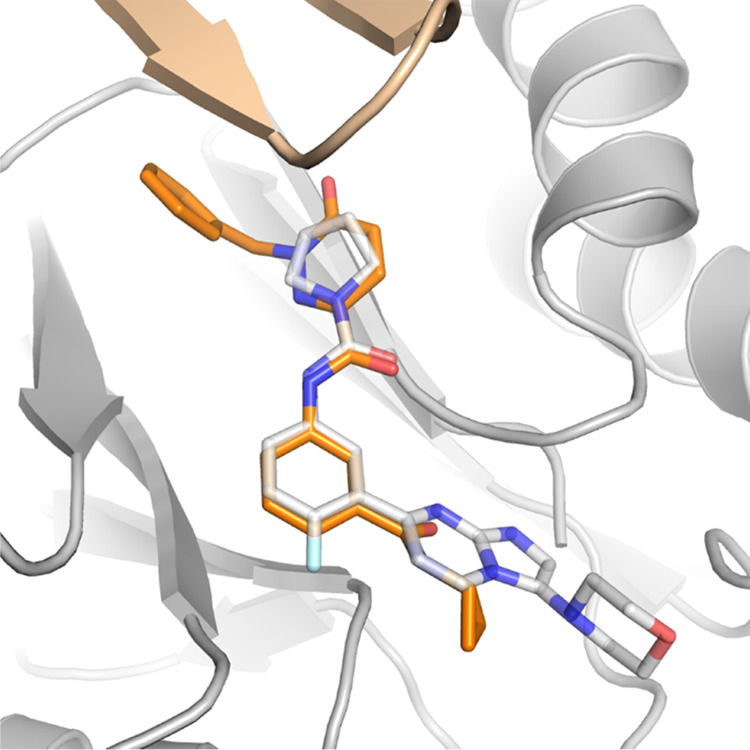
Overlay of **5** (C atoms gold) and **1** (C atoms gray) docked into the *T. cruzi* 20S proteasome homology model (based on PDB 8OLU).

Comparing the binding poses of **5** and **1** shows the central amide of **5** overlapping with the urea of **1**, the pyridazinone of **5** occupying the same space as the pyrrolidine group of **1** and the phenyl ring of **5** overlapping with the fluorophenyl moiety of **1**. The propyl amide group of **5** then occupies some of the space occupied by the bicyclic system of **1**. This suggested that transfer of structure–activity relationship (SAR) between the chemotypes might be possible (Figure 6). This was also supported by the MP2-FMO calculations, which suggested that potency could be increased by improved interaction with β5Thr1 (via extension of the cyclopropyl amide), and by fluorination of the central phenyl ring to increase its dipole and further strengthen the interaction with β5Asp115 and β5Asp116. The importance of the hydrophobic interactions of the benzyl group also showed that there would be limited value in making diverse changes in this position. Molecular development would therefore focus on fluorination of the central phenyl ring, substitutions to the benzyl moiety extending into an induced fit area of the binding site, and the amide vector extending further into the space occupied by **1**.

### Structure–Activity Relationship

Equipped with this structural information, starting from hit compound **5**, we embarked on a systematic exploration of the molecule to identify a compound suitable for an *in vivo* proof-of-concept study. The desired compound would have *T. cruzi* pIC_50_ > 6, aqueous solubility > 100 μM, intrinsic clearance <3 mL/min/g (mouse liver microsomes), and ER < 3. We initially explored changes to the central pyridazinone and phenyl groups of **5** (Table 3). Capping the amide nitrogen with a methyl gave **6**, with a complete loss of activity confirming that this NH was involved in a key hydrogen-bond interaction with β5Tyr113. Capping the terminal amide nitrogen also led to a significant loss in potency (**7**). Removing a nitrogen atom from the pyridazinone to give pyridone **8** led to a small drop in potency against the *T. cruzi* proteasome but a loss of measurable activity in the *T. cruzi* antiparasitic assay. Both the known SAR from **1** and the output of the MP2-FMO calculations suggested adding a fluorine in the 4-position of the central phenyl ring would result in improved potency. Pleasingly, as predicted, the addition of fluorine in this position to give **9** resulted in >0.5 log unit boost in potency. **9** also showed high aqueous solubility, but the microsomal intrinsic clearance was not suitable for progression to *in vivo* studies. Moving the fluorine to the 2-position to give **10** was less successful, this compound being 10-fold less potent than **9**. Again, this matched the SAR observed in the earlier series.^16^

**Table 3:**
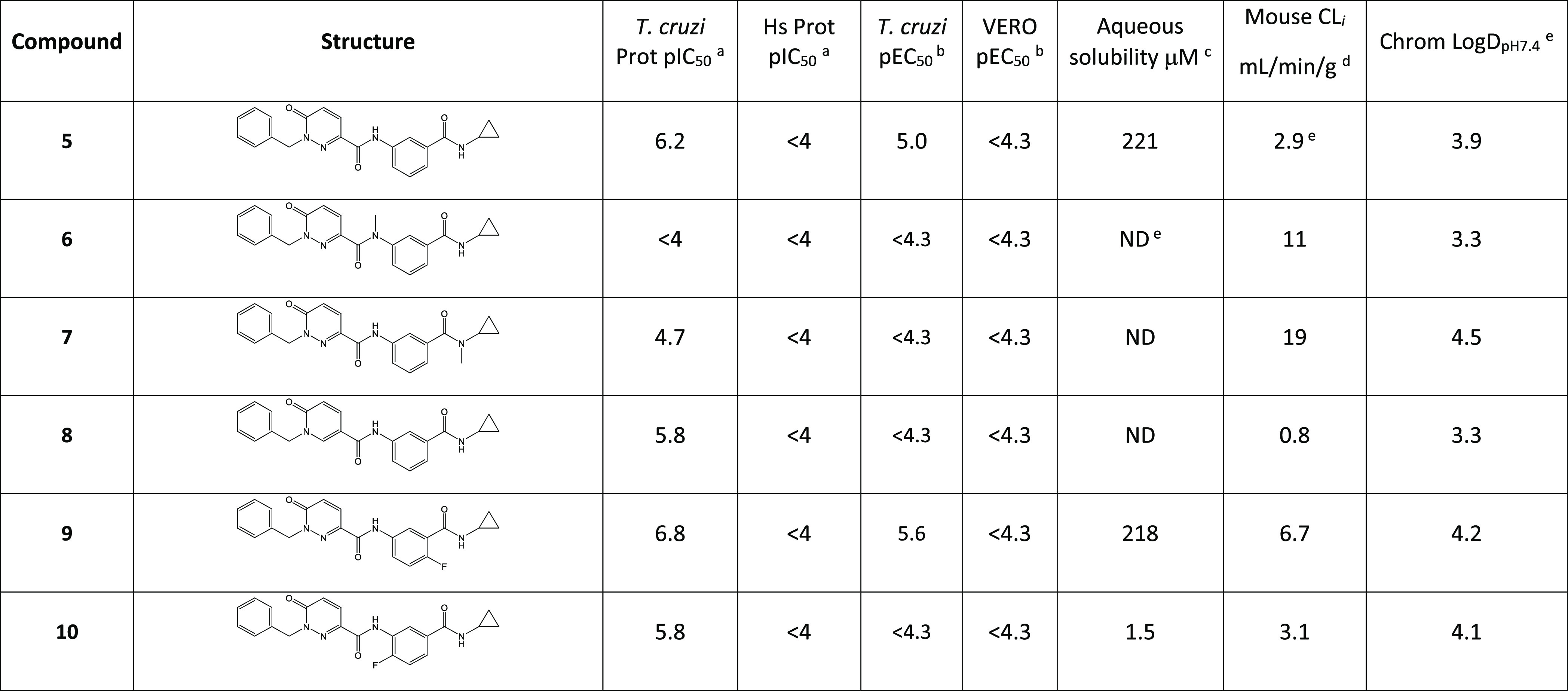


a Inhibition of *T. cruzi* and human proteasome, data from at least three independent replicates, standard deviations ≤0.2. All data are provided in the Supporting Information.

b Potency against intracellular *T. cruzi* amastigotes and host VERO cells, data from at least three independent replicates, standard deviations ≤0.2. All data are provided in the Supporting Information.

c Kinetic aqueous solubility measured by a UHPLC system equipped with a UV/visible and single-quadrupole mass spectrometer.

d Mouse liver microsomal intrinsic clearance, scaling factor used is 52.5 mg of microsomal protein/g liver.

e Chrom Log *D*_pH7.4_ = CHI_pH7.4_ × 0.0857 −2, where CHI is the chromatographic hydrophobicity index.

f ND: Not Determined.

Having identified compound **9** which had improved potency compared to the hit compound **5**, we used this as the start-point for the investigation of the benzyl substituent of the pyridazinone with the aim of improving potency and intrinsic clearance while maintaining the high solubility of **5** and **9** (Table 4). Saturation of the benzyl ring to give the cyclohexyl compound **11** resulted in a loss of potency, likely due to less optimal hydrophobic interactions with β4Phe24 and β5Tyr113. This compound also showed no improvement in microsomal intrinsic clearance. Based on the binding pose, we hypothesized that the addition of a 4-methoxy substituent to **9** could pick up a polar interaction with the side chain of β4Asn22. Pleasingly, this led to **12** with a 0.7 log unit increase in activity against the enzyme which translated to improved activity in the antiparasitic cell-based assay. Microsomal intrinsic clearance was also significantly improved compared to the unsubstituted benzyl compound **5** although solubility was decreased. Moving the methoxy substituent to the 3-position of the phenyl ring to give **13** resulted in a complete loss of potency against the *T. cruzi* proteasome. Retaining the 4-methoxy substituent and adding a chlorine (**14**) or a fluorine (**15**) in the 3-position resulted in a further improvement in potency, with pIC_50_ > 8 against the *T. cruzi* proteasome. **15** was also run in the MDR1-MDCK assay, showing good passive permeability (546 nm s^–1^) and a low ER of 3.0, although it still showed poor solubility (4 μM in the aqueous solubility assay and 11 μM FaSSIF solubility), precluding its progression to *in vivo* studies. As **14** and **15** showed reasonable intrinsic clearance but poor potency, we investigated alternative 3,4-disubstituted benzyl groups. A similar profile to **14** and **15** was observed for the 4-methoxy-3-nitrile compound **16**, while 4-chloro-3-fluoro analogue **17** exhibited good potency but was hampered by higher intrinsic clearance compared to the methoxy analogues. Adding a nitrogen into the ring to give **18** had a negative impact on potency with over a log unit drop compared to the matched pair compound **15**. This compound also showed inferior solubility and intrinsic clearance, possibly due to its increased Chrom LogD.

**Table 4:**
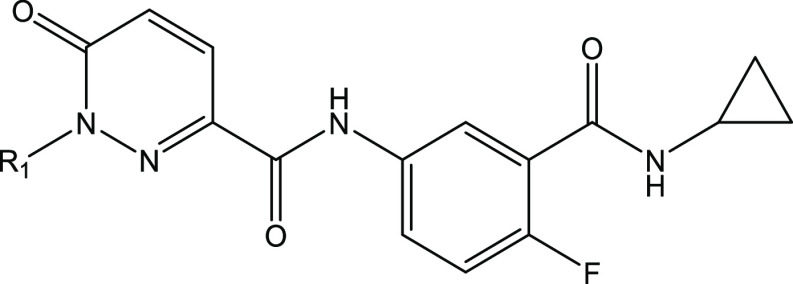

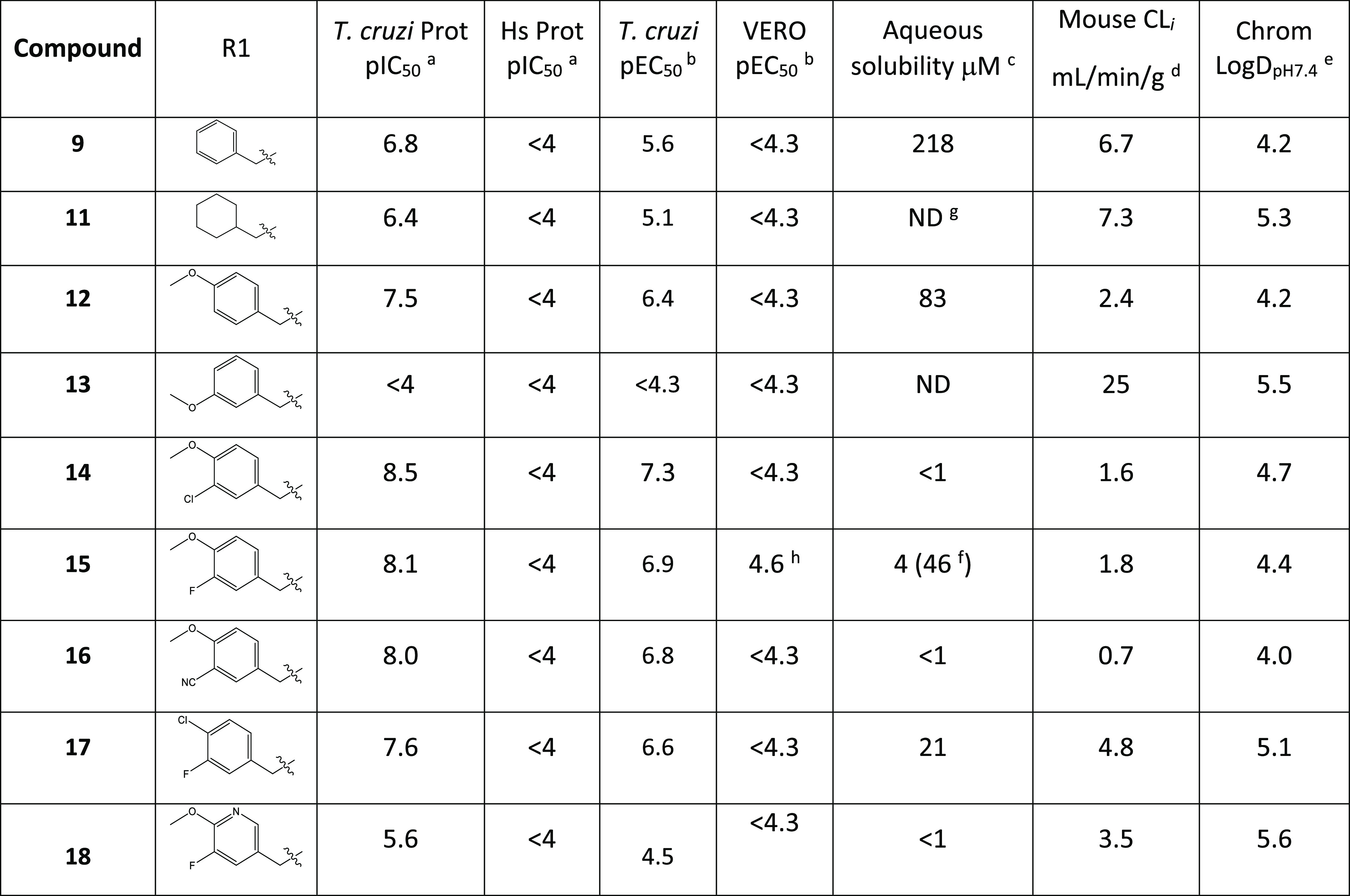


a Inhibition of *T. cruzi* and human proteasome, data from at least three independent replicates, standard deviations ≤0.2. All data are provided in the Supporting Information.

b Potency against intracellular *T. cruzi* amastigotes and host VERO cells, data from at least three independent replicates, standard deviations ≤0.2. All data are provided in the Supporting Information.

c Kinetic aqueous solubility measured by a UHPLC system equipped with a UV/visible and single-quadrupole mass spectrometer.

d Mouse liver microsomal intrinsic clearance, scaling factor used is 52.5 mg of microsomal protein/g liver.

e Chrom Log *D*_pH7.4_ = CHI_pH7.4_ × 0.0857 – 2, where CHI is the chromatographic hydrophobicity index.

f Number in parentheses refers to FaSSIF solubility (fasted state simulated intestinal fluid solubility, μM).

g ND: Not Determined.

h Inactive against Vero cells in seven replicates, potency shown is from one active replicate.

**Figure 7:**
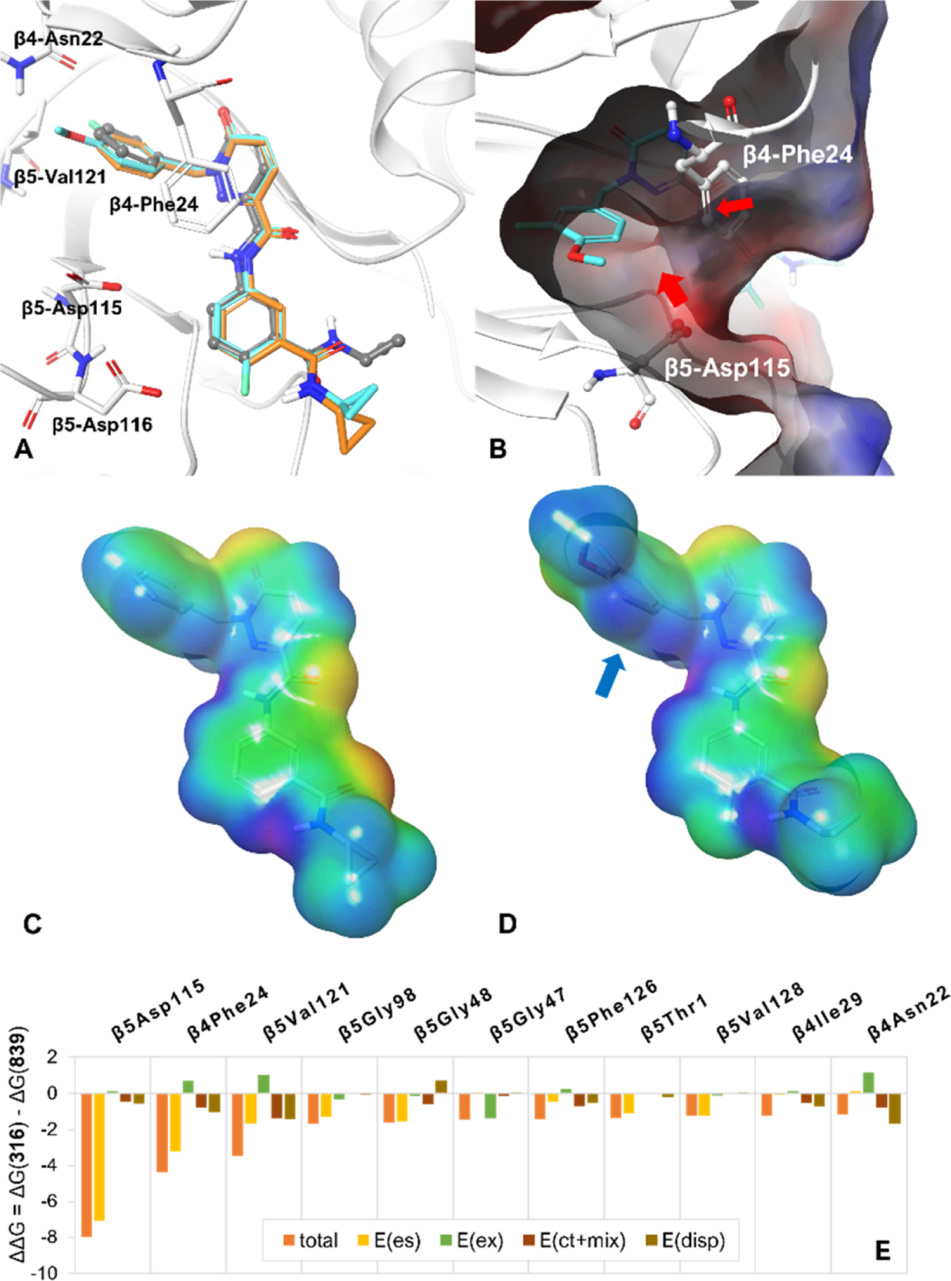
*In silico* analysis of the effect of benzyl substituents. (A) Docked poses of **5** (C atoms orange) and **15** (C atoms cyan) to the *T. cruzi* β4/β5 20S homology model, overlaid with the structure of **5** from the *L. tarentolae* crystal complex (gray, from PDB 8OLU). (B) Poisson–Boltzmann ESP analysis of the protein surface of the *T. cruzi* β4/β5 20S homology model, focussed around the benzyl substituent of **5**. (C, D) Electrostatic potential surface calculated using B3LYP-D3 theory and HF-6-31G** basic set in Jaguar for the molecular surface of **5** (C) and **15** (D), respectively. (E) MP2-FMO PIEDA analysis: residues with lower than −1 kcal·mol^–1^ ΔΔ*G* between MP2-FMO PIEDA Δ*G* of **15** and **5** are included. Different components of the total energies are also shown. Note that both β5Asp115 and β4Phe24 have ΔΔ*G* contribution mainly from electrostatic components, E(es). Blue and red arrows in (B) and (D) indicate the electrostatic complementarity area of benzyl group to β5Asp115 and β4Phe24. Abbreviations for energetic components: E(es): electrostatic, E(ex): exchange repulsion, E(ct+mix): charge transfer, E(disp): dispersion.

Docking studies using the *T. cruzi* β4/β5 20S proteasome homology model show very high consistency between the docked poses of **5** and **15**, compared to the *L. tarentolae* cryo-EM complex of **5** (Figure 7A). Comparison of the MP2-FMO analysis of **5** and **15** in the *T. cruzi* homology model confirmed the orientation of the benzyl head group, with the 4-methoxy substituent interacting with β4Asn22 (indicated by a −1.16 kcal·mol^–1^ improvement (ΔΔ*G*) in energy contribution) as expected, and also improving the interaction with β4Phe24 (ΔΔ*G* = −4.38 kcal·mol^–1^). Also as expected, the fluorine oriented toward β4Val121 (ΔΔ*G* = −3.45 kcal·mol^–1^), while also giving a more significant energetic contribution with β5Asp115 (ΔΔ*G* = −7.99 kcal·mol^–1^) within a total calculated binding energy improvement of −18.75 kcal·mol^–1^ going from **5** to **15**. Interestingly, the contribution from β4Phe24 and β5Asp115 is mainly through electrostatic interactions, rather than any other forms of energetics (Figure 7E). This corresponds well with electrostatic potential (ESP) analysis of both the protein and the compounds (Figure 7B–D) where the methoxy and fluoro substituents of **15** induce an uneven electrostatic distribution about the benzyl group, with partial positivity (Figure 7D) exposed to the side chains of β4Phe24 and β5Asp115, which both maintain partial negativity (Figure 7B). This electrostatic complementarity is likely facilitating the activity boost, alongside the dispersive component of β4Phe24.

Comparing the binding modes of **5** and **1** suggested there was an opportunity to expand the amide vector, with the MP2-FMO calculations showing that there might be the opportunity to pick up an interaction with β5Thr1. With the optimized benzyl substituents of **12** (4-methoxy) and **15** (3-fluoro-4-methoxy), we investigated alternative substitutions on the amide, aiming to retain (or improve) potency while improving solubility and intrinsic clearance. To explore this vector, we synthesized both 4-methoxy and 3-fluoro-4-methoxybenzyl analogues, as the latter generally led to more potent, but less soluble, compounds, and it was important to identify the molecules with the best overall profiles (Table 5). The 3-methoxy cyclobutene analogues **19** and **20** showed good potency and intrinsic clearance but were hampered by poor solubility. Morpholine analogues **21** and **22** showed much improved solubility profiles; however, intrinsic clearance was higher than desired and **22** was found to be a Pgp substrate when assessed in the MDR1-MDCK assay (ER 13.3). The 3-morpholino-cyclobutyl analogues **23** and **24** exhibited high potency. While *cis* isomer **24** suffered from low solubility, *trans* isomer **23** showed a good balance of properties but was found to be a Pgp substrate with ER of 22.2. Amino alcohol compound **25** also showed good potency against the target and in the whole cell assay as well as exhibiting good solubility and low intrinsic clearance. Unfortunately, this compound also had low passive permeability in the MDR1-MDCK assay (133 nm s^–1^) with a high ER of 21.2. As the extended analogues were not improving potency and did not deliver compounds with suitably balanced properties for progression to *in vivo* studies, we investigated the effect of truncating the amide moiety. Methyl amide **26** maintained similar potency to the more elaborated analogues and had very good overall properties with high passive permeability, an ER of 1.8, and FaSSIF solubility measured at 339 μM, making this compound of particular interest. As expected, fluorination of the phenyl ring of **26** to give compound **27** increased potency in both the biochemical and cellular assays, as well as improving intrinsic clearance. Unfortunately, **27** was significantly less soluble than **26**, so it was not progressed further.

**Table 5:**
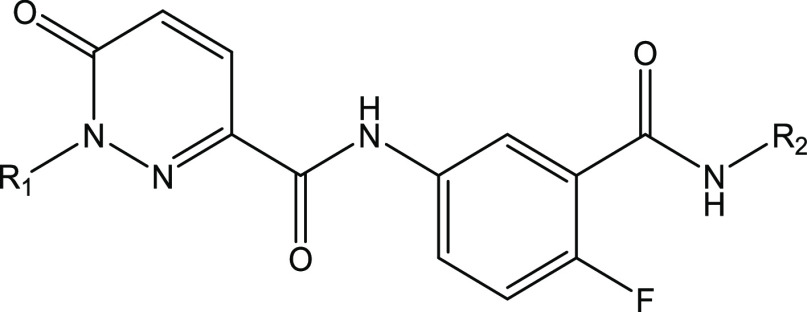

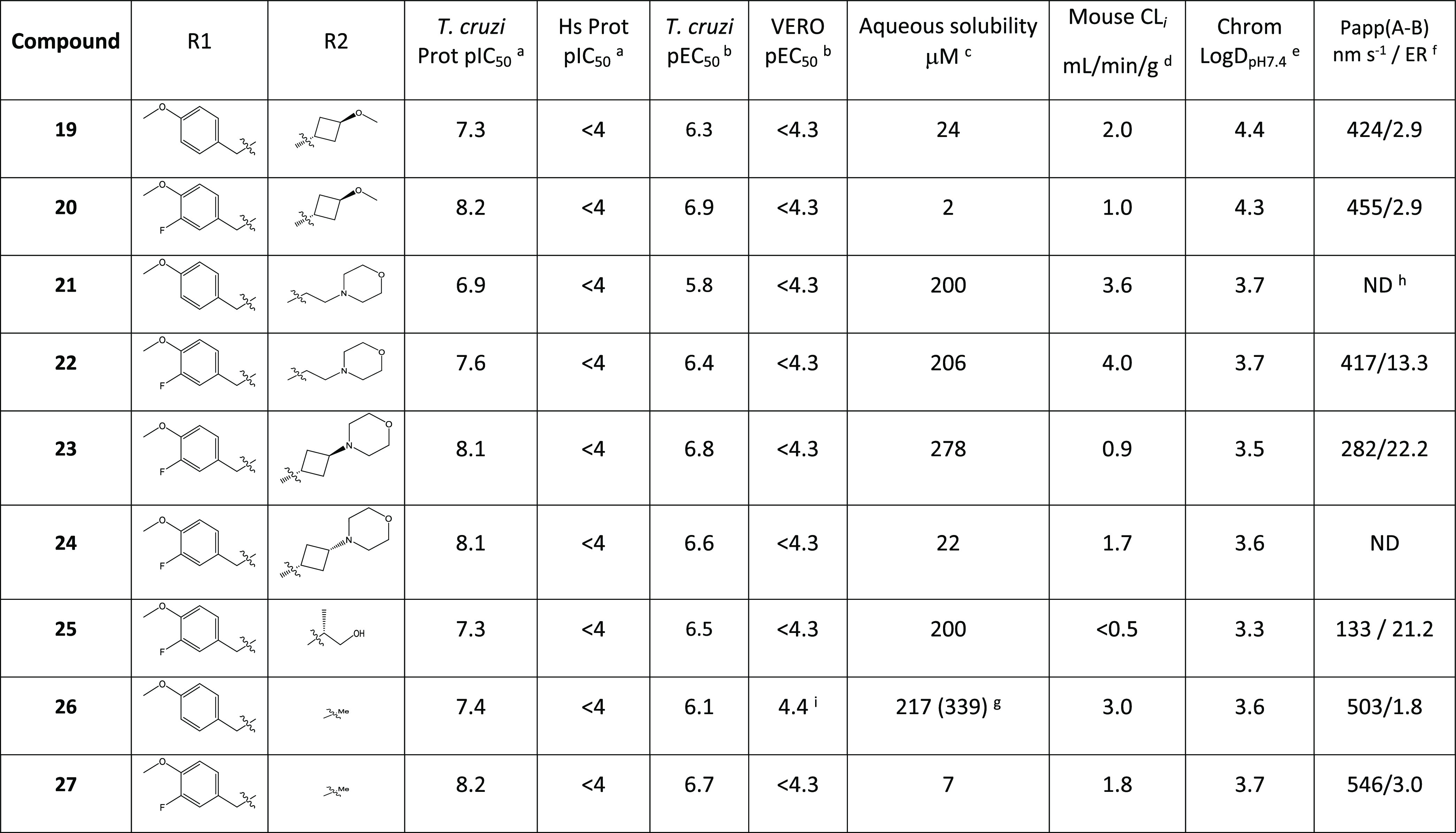


a Inhibition of *T. cruzi* and human proteasome, data from at least three independent replicates, standard deviations ≤0.2. All data are provided in the Supporting Information.

b Potency against intracellular *T. cruzi* amastigotes and host VERO cells, data from at least three independent replicates, standard deviations ≤0.2. All data are provided in the Supporting Information.

c Kinetic aqueous solubility measured by a UHPLC system equipped with a UV/visible and single-quadrupole mass spectrometer.

d Mouse liver microsomal intrinsic clearance, scaling factor used is 52.5 mg of microsomal protein/g liver.

e Chrom Log *D*_pH7.4_ = CHI_pH7.4_ × 0.0857 −2, where CHI is the chromatographic hydrophobicity index.

f Permeability in MDR1-transfected MDCK cells in the presence of a Pgp inhibitor (GF120918)/efflux ratio.

g Number in parentheses refers to FaSSIF solubility (fasted state simulated intestinal fluid solubility, μM).

h ND Not Determined.

i Inactive against Vero cells in 9 out of 10 replicates, data for active replicate shown.

**Figure 8:**
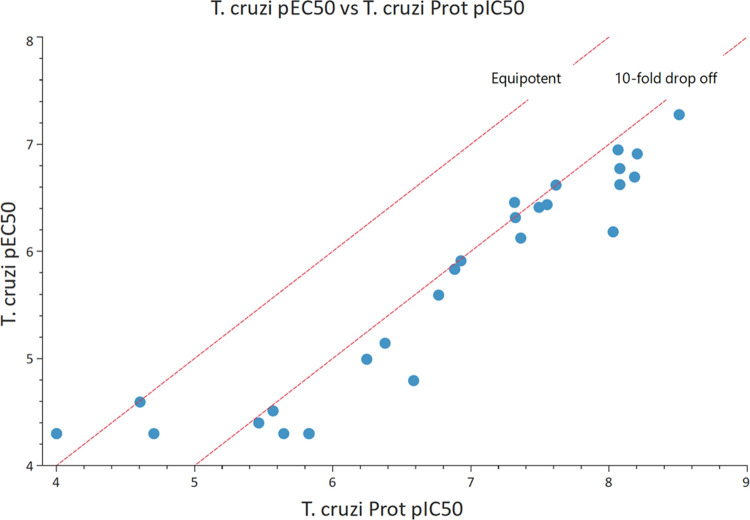
*T. cruzi* proteasome pIC_50_ vs *T. cruzi* pEC_50_ for compounds **5**–**27**, demonstrating a strong correlation, with a 10-fold drop-off in activity.

**Figure 9:**
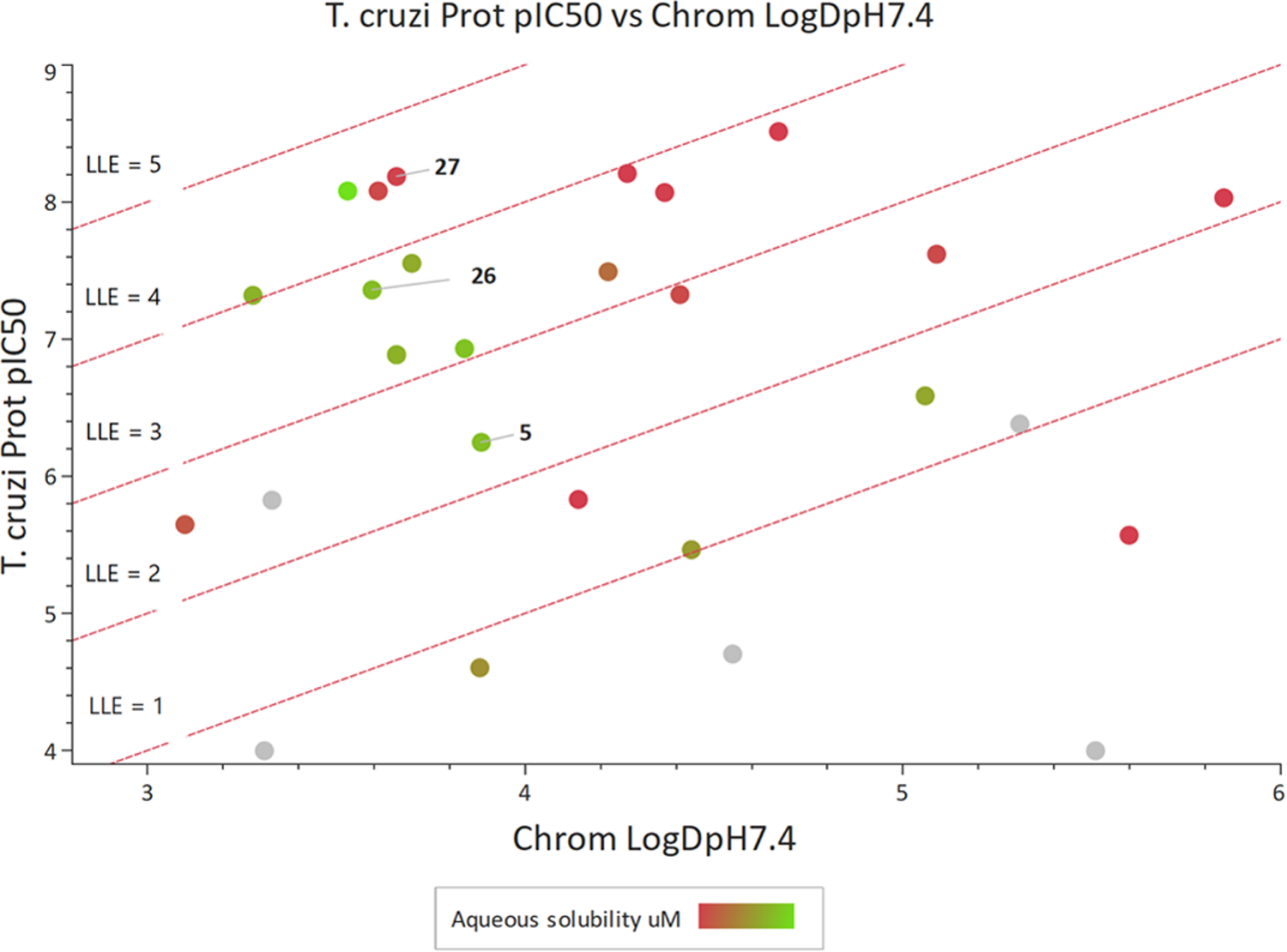
Chrom Log *D*_pH7.4_ vs *T. cruzi* Prot pIC_50_ (LLE plot) for compounds **5**–**27**, colored by solubility from low (red, <10 μM) to high (green, >200 μM), (gray = not determined).

For the synthesized compounds, a plot of proteasome inhibition against antiparasitic activity showed a clear correlation (Figure 8), with a relatively consistent 10-fold drop-off in activity against the parasite, suggesting that the antiparasitic activity was being driven by proteasome inhibition. Also, a plot of Chrom Log *D* vs *T. cruzi* proteasome inhibition (Figure 9) showed that it was possible to increase potency from the initial hit, while reducing lipophilicity. This focus on compounds that increased LLE by reducing lipophilicity was critical to identifying compounds that coupled good levels of potency with desirable solubility and metabolic stability.

### *In Vivo* Studies on **15** and **26**

**Figure 10:**
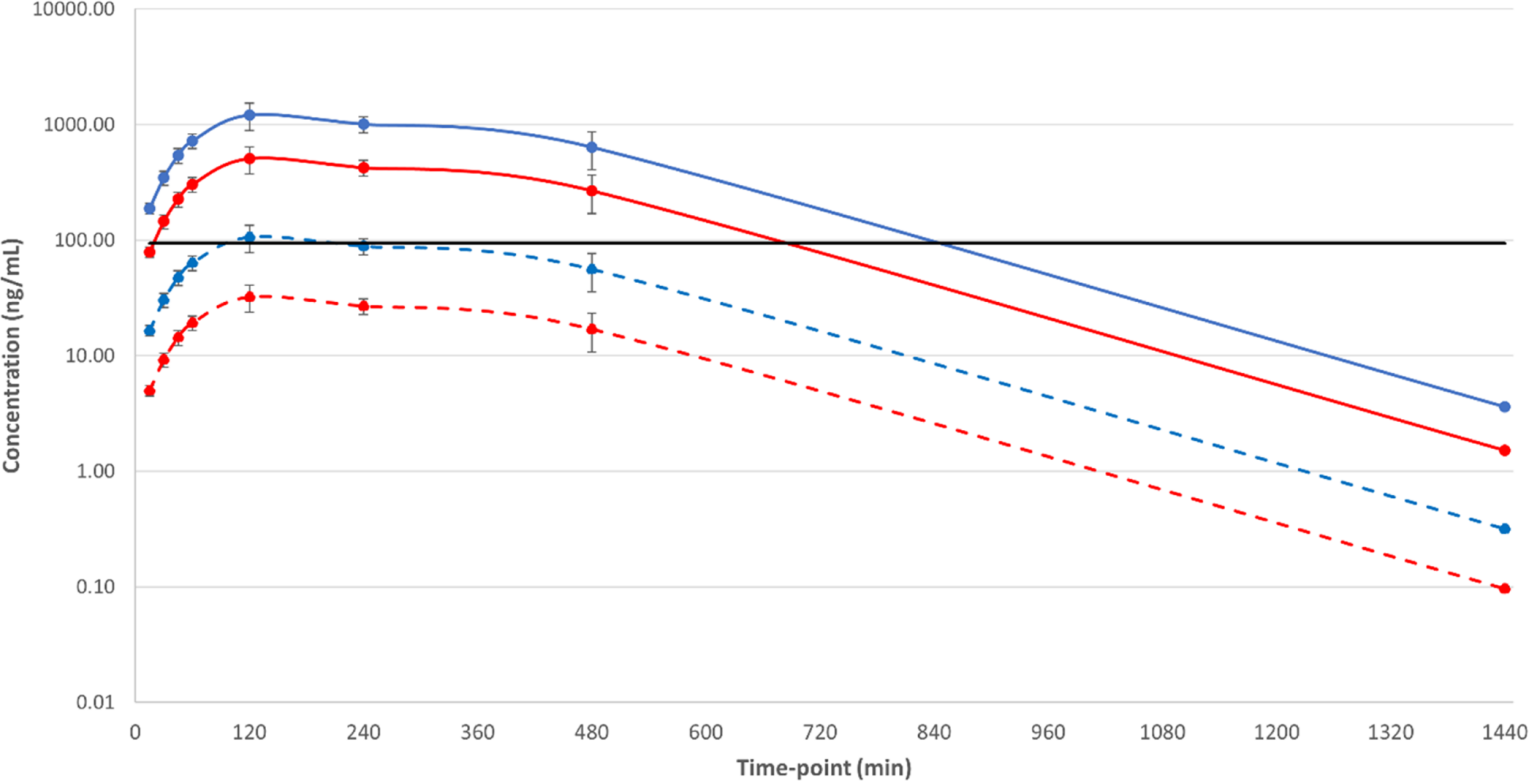
Blood exposure and predicted CNS exposure of **15** after a single 50 mg/kg p.o. dose to Balb-c mice. Solid blue line = whole blood concentration; solid red line = predicted total brain concentration; dashed blue line = free blood concentration; dashed red line = predicted free brain concentration based on brain tissue free fraction of 6.4%; solid black line = *T. cruzi* EC_99_.

From the chemistry program, compounds with suitable profiles to progress to *in vivo* studies were identified. Initially, we investigated **15** due to its high intracellular potency, low intrinsic clearance in mouse liver microsomes, and reasonable permeability. The ER of 6, coupled with high passive permeability (Papp(A–B) > 300 nm/s) was an improvement on compound **4**, although aqueous solubility in both buffer and FaSSIF media was low. When dosed orally at 50 mg/kg in mice, **15** gave total blood levels above EC_99_ for around 14 h (Figure 10), although accounting for the blood–brain ratio of 0.42 it was predicted that total brain concentration would only reach the EC_99_ for approximately 12 h, with the unbound brain concentration never reaching EC_99_.

**Figure 11:**
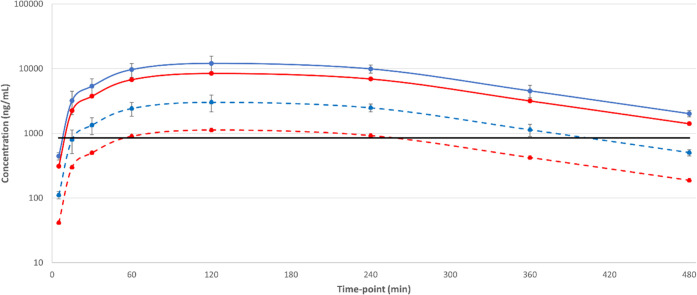
Blood exposure and predicted CNS exposure of **26** after a single 50 mg/kg p.o. dose to Balb-c mice. Solid blue line = whole blood concentration; solid red line = predicted total brain concentration; dashed blue line = free blood concentration; dashed red line = predicted free brain concentration; solid black line = *T. cruzi* EC_99_.

We therefore proceeded to examine **26**, which had similar potency to **15**. As seen for **4**, **26** performed well in our *in vitro* washout assay, with relapses at 35 and 42 days post washout compared to 31 and 35 days for BNZ (two replicates each, 16-day treatment at 25-fold EC_50_). Although intrinsic clearance in mouse liver microsomes was poorer, **26** did show much improved solubility and permeability, a lower ER of 1.8, and a brain–blood ratio of 0.70. **26** was therefore progressed into a pharmacokinetic (PK) study, dosed at 50 mg/kg, and was seen to have total blood exposure exceeding the EC_99_ for >8 h, with unbound exposure above EC_99_ for approximately 7 h (Figure 11). Based on the brain–blood ratio, and the measured brain tissue binding (13.3%), brain total concentration was likely to exceed EC_99_ for around 7 h, with brain unbound concentration above EC_99_ for around 3 h.

**Figure 12:**
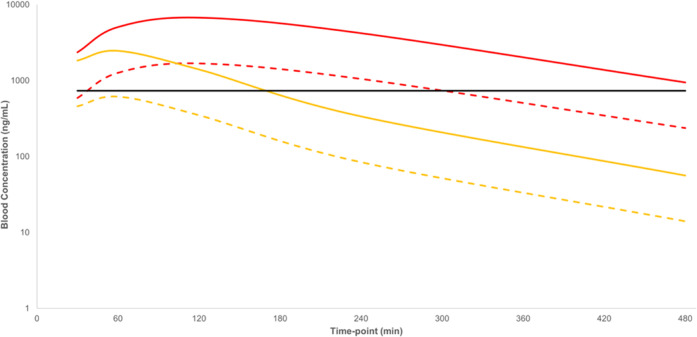
Total and free blood concentrations for compound **26** from a 20-day chronic mouse efficacy model of Chagas disease dosed at 50 mg/kg PO b.i.d., showing total/free exposure after initial doses on days 1 and 20. Solid red line = whole blood exposure, day 1; dashed red line = free blood exposure, day 1; solid yellow line = whole blood exposure, day 20; dashed yellow line = free blood exposure, day 20; solid black line = *T. cruzi* EC_99_. Dosing was initiated 135 days post-infection.

Based on this data, we considered **26** to be a suitable compound to give proof of concept in the chronic mouse model of Chagas disease. We elected to dose at 50 mg/kg b.i.d for 20 days to maximize the chances of clearing all of the parasites. Unfortunately, **26** was not curative, compared to benznidazole which cured all mice at the dose given (100 mg/kg for 20 days, u.i.d). Analysis of the blood levels of **26** taken on days 1 and 20 of the study showed that, as expected, free exposure was above EC_99_ for approximately 5 h after the first dose on day 1. However, by day 20, this had dropped to less than 2 h after the first dose (Figure 12). **26** showed no effect on mouse PXR, even at 30 μM, so autoinduction due to PXR-mediated upregulation of CYP_450_ enzymes was unlikely to be the explanation.

The ability of **4** and **26** to outperform BNZ in our *in vitro* washout assay, alongside the results reported for **2**, suggests that proteasome inhibition is a valid strategy for targeting *T. cruzi*. Unfortunately, we were unable to translate this into efficacy in a chronic mouse model of Chagas disease for **26**. The most likely explanation is that blood-free levels were not maintained above the EC_99_ for the total dosing duration. Furthermore, the brain exposure data for **26** indicated that it also may not reach all key tissues with sufficient levels of free drug. During washout assays exposure is kept high (>EC_99_) continuously for the duration of treatment, while in the *in vivo* studies, free compound exposure for **26** dropped below EC_99_ after ∼5 h post-dosing on day 1 and even sooner on day 20, potentially allowing the parasites to recover each day. This issue could potentially have been addressed using higher doses of **26** to increase exposure, but a dose escalation study was run which showed that **26** was not tolerated at higher doses (data not shown). Unfortunately, this meant that our hypothesis of utilizing CNS exposure to predict potential efficacy was not fully tested, and whole-body imaging experiments are currently ongoing to better understand the *in vivo* distributional characteristics of key compounds within the series compared to parasite localization. This will be reported in a subsequent publication.

### Synthetic Chemistry Section

**Scheme 1:**
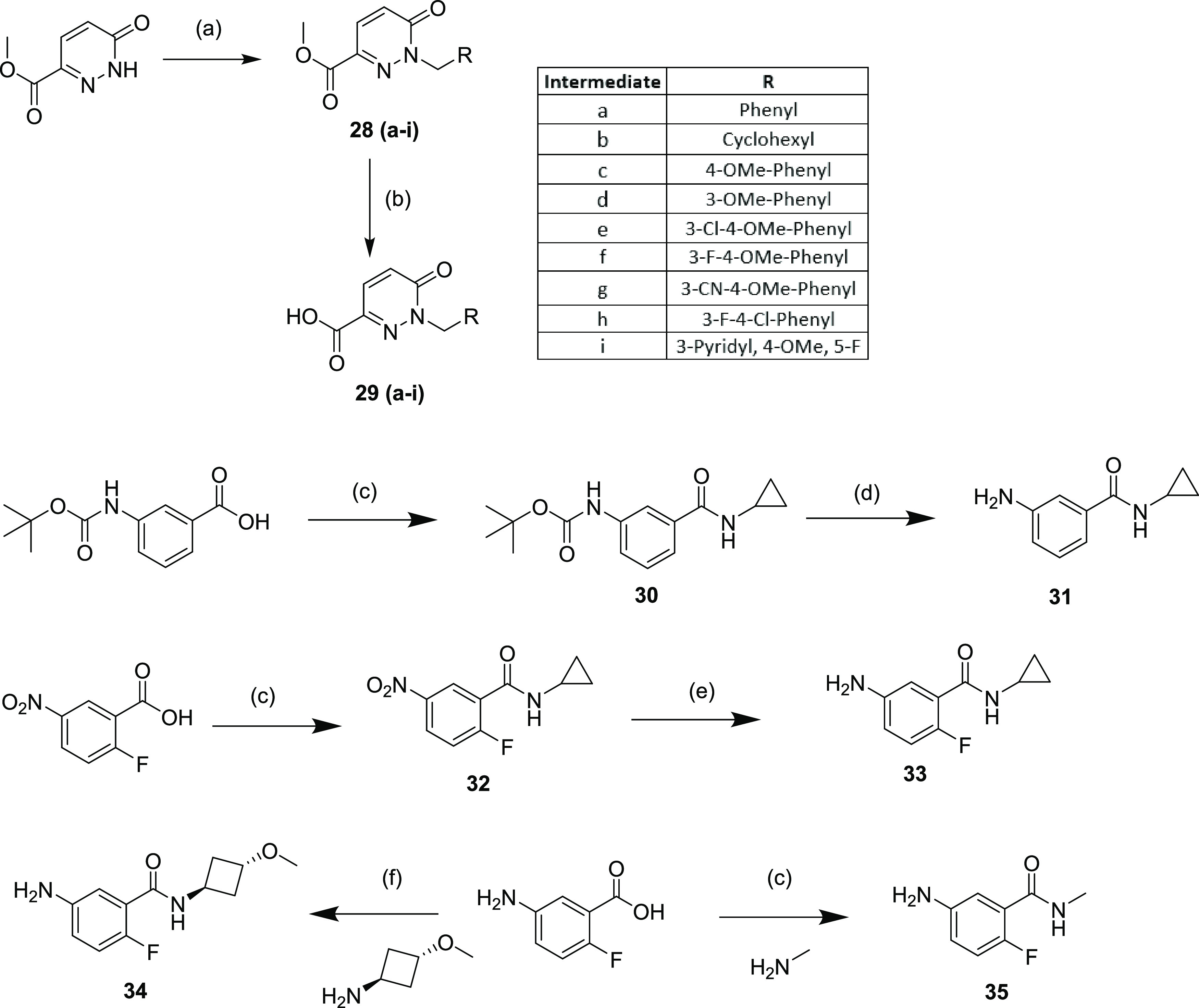
Synthesis of Intermediates **29 (a–i)**, **31**, and **33–35** Reaction and conditions: (a) R_1_Hal, K_2_CO_3_ (Cs_2_CO_3_ for **28b/c**), MeCN, RT (43–87%); (b) NaOH, EtOH, RT (K_2_CO_3_, *N*,*N*-dimethylformamide (DMF), water for **29d**) (84–99%); (c) T3P (50 wt % in EtOAc), DIPEA, relevant amine, RT (27–74%); (d) 4 M HCl, dioxane, RT, 100%; (e) Fe, NH_4_Cl, RT (77%); (f) EDC, HOBt, DIPEA, RT (74%).

To facilitate the synthesis of the described compounds, we first synthesized a set of key building blocks. Initially, alkylated pyridazinone 3-carboxylic acids were synthesized from methyl 6-oxo-1,6-dihydropyridazine-3-carboxylate, starting by alkylation of the pyridazinone nitrogen with a suitable alkyl halide to give **28** (**a**–**i**) with subsequent ester hydrolysis yielding the acids **29** (**a**–**i**) (Scheme 1). Alongside these, anilines **31**, **33**, **34**, and **35** were synthesized. Initially, BOC-protected 3-aminobenzoic acid was coupled with cyclopropylamine using T3P to give **30**, with subsequent BOC deprotection yielding aniline **31**. Alternatively, 2-fluoro-5-nitrobenzoic acid was coupled to cyclopropylamine using T3P to yield **32**, which was reduced with iron/ammonium chloride to yield aniline **33**. Finally, 5-amino-2-fluorobenzoic acid was coupled directly with *trans*-3-methoxycyclobutan-1-amine using EDC/HOBt to give **34**, or coupled with methylamine using T3P/*N*,*N*-diisopropylethylamine (DIPEA) to give **35**. These intermediates could be utilized for the synthesis of the target compounds following two different routes.

**Scheme 2:**
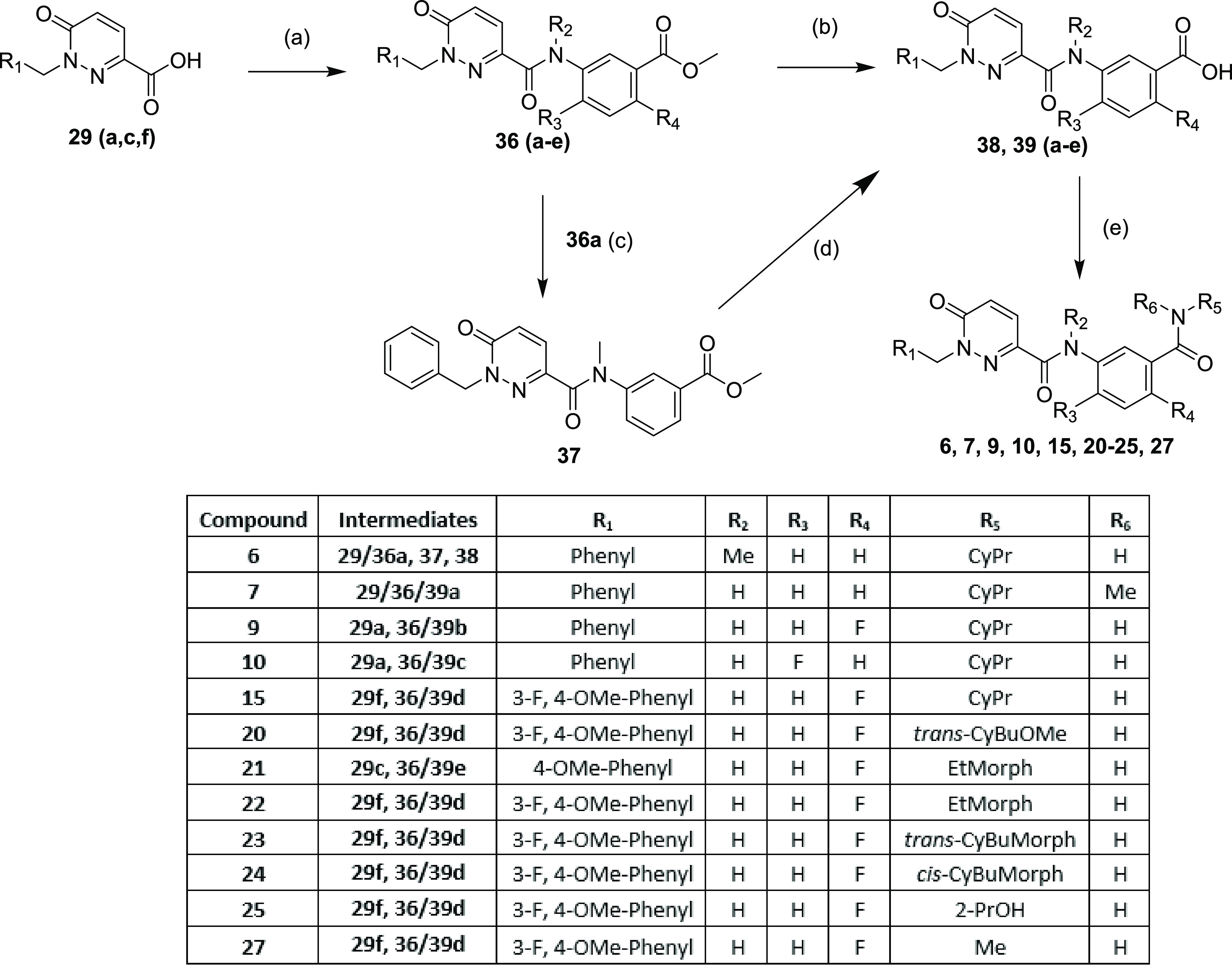
Synthesis of Compounds **6**, **7**, **9**, **10**, **15**, **20**–**25**, and **27** Reagents and conditions: (a) T3P (50 wt % in EtOAc) (or HATU), DIPEA, Relevant aniline, dichloromethane (DCM), RT (46–99%); (b) NaOH, EtOH, RT (16–99%); (c) MeI, K_2_CO_3_, DMF, RT, 87%; (d) LiOH, tetrahydrofuran (THF), 0 °C (41%); (e) T3P (or HATU), DIPEA, amine, RT (6–99%).

Following a linear route, the benzoic acids **29** (**a**, **c**, **f**) could be coupled with suitable anilines *via* T3P or HATU to give amides **36** (**a**–e) (Scheme 2). To generate the methylated amide, **36a** was treated with iodomethane and potassium carbonate to give **37**. Then, with all of these amides in hand, ester hydrolysis yielded acids **38** and **39**(**a–e**), with a second amide coupling to the relevant amine yielding compounds **6**, **7**, **9**, **10**, **15**, **20**–**25**, and **27**.

**Scheme 3:**
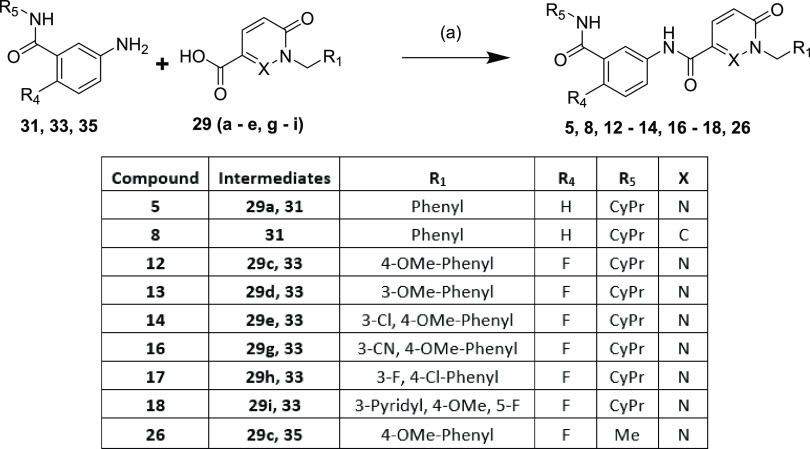
Synthesis of Compounds **5**, **8**, **12**–**14**, **16**–**18**, and **26** Reaction and conditions: (a) T3P (50 wt % in EtOAc) or HATU, DIPEA, amine, RT (6–90%)

**Scheme 4:**
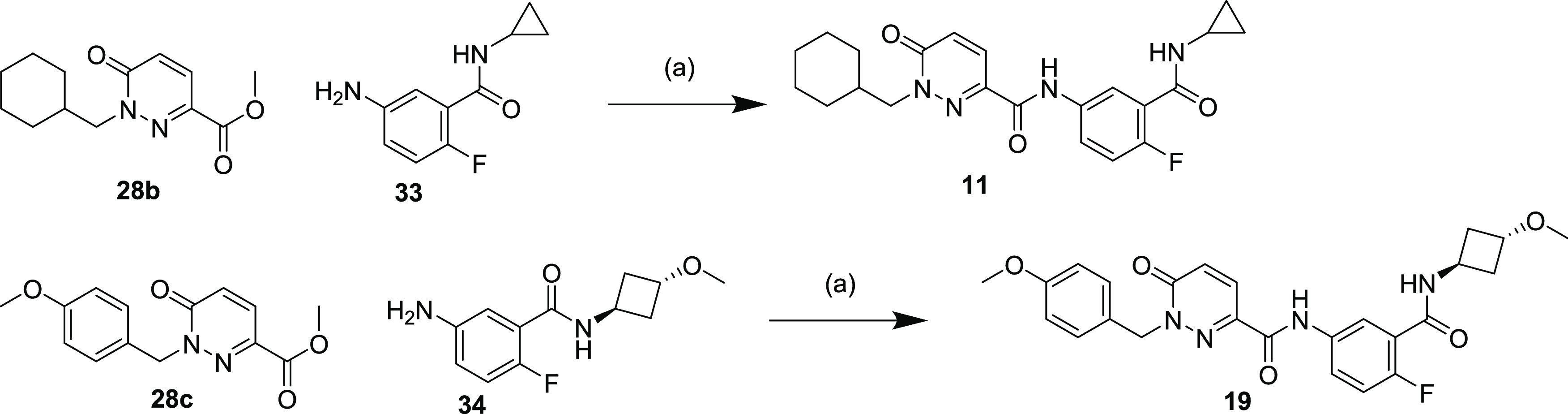
Synthesis of Compounds **11** and **19** Reaction and conditions: (a) Me_3_Al, toluene, RT (23% for **11**, 95% for **19**).

Alternatively, a more convergent route could be utilized (Scheme 3) where anilines **31**, **33**, and **35** were coupled with the relevant 1-substituted pyridazinone 3-carboxylic acid (**29a**, **c**–**e**, **g**–**i**) to yield compounds **5**, **8**, **12**–**14**, **16**–**18**, and **26**. Finally, esters **28b** and **28c** could be directly coupled to anilines **33** and **34**, respectively, using trimethylaluminum, to yield compounds **11** and **19** (Scheme 4).

## Conclusions

Based on our *in vitro* data package, alongside previously reported work,^24^ inhibition of the proteasome appears to be an attractive mechanism of action for new drugs for the treatment of Chagas disease. Our previously identified series of *Leishmania* proteasome inhibitors failed to provide compounds with activity in a chronic mouse model of Chagas disease, which we hypothesized was due to a disconnection between the distribution of compound *vs* parasite *in vivo*. We therefore undertook a hit-discovery program which led to the identification of **5** as an attractive hit compound. A cryo-EM structure of **5** bound to the *L. tarentolae* proteasome showed that, like the previous series, it bound at the β4/β5 interface close to the catalytic β5Thr100, although it also occupied an additional hydrophobic pocket. Utilizing the structural information, hit expansion of **5** led to the identification of two compounds, **15** and **26**, with suitable profiles to progress to *in vivo* studies. **26** proved to have a much more attractive *invivo* profile compared to **15** and was therefore progressed into a mouse efficacy model of chronic Chagas disease, but disappointingly was not curative. The reasons for this were likely related to blood exposure not being maintained above EC_99_ for the whole dosing interval, and limited distribution to all of the sites where the parasite resides. Although this series did not deliver efficacious compounds, proteasome inhibition remains an attractive strategy for targeting Chagas disease and other kinetoplastid-related diseases. To help understand if compounds are distributed to locations where the parasites are residing within the mouse, whole-body imaging experiments are ongoing, which will map compound distribution to where the *T. cruzi* parasites reside. If the compound is not distributed to all of the sites where the parasites reside, this could explain the lack of efficacy of **26**. These studies will help in the design of the next generation of *T. cruzi* proteasome inhibitors and will be reported in a subsequent publication.

## Experimental Section

### Chemistry

Chemicals and solvents were purchased from the Aldrich Chemical Company, Fluka, ABCR, VWR, Acros, Fluorochem, and Alfa Aesar and were used as received. Air- and moisture-sensitive reactions were carried out under an inert atmosphere of nitrogen in oven-dried glassware. Flash column chromatography was performed using pre-packed silica gel cartridges (230–400 mesh, 40–63 μm, from Redisep) using a Teledyne ISCO Combiflash Companion, or Combiflash Retrieve. ^1^H NMR and ^13^C NMR spectra were recorded on a Bruker Avance DPX 500 spectrometer (^1^H at 500.1 MHz, ^13^C at 125.8 MHz). Chemical shifts (δ) are expressed in ppm recorded using the residual solvent as the internal reference in all cases. Signal splitting patterns are described as singlet (s), doublet (d), triplet (t), quartet (q), multiplet (m), broad (br), or a combination thereof. Coupling constants (*J*) are quoted to the nearest 0.1 Hz. High-resolution electrospray measurements were performed on a Bruker Daltonics MicrOTOF mass spectrometer. Low-resolution electrospray (ES) mass spectra were recorded on an Advion Compact mass spectrometer (CMS: model ExpressIon CMS) connected to Dionex Ultimate 3000 UPLC system with diode array detector, or an Acquity UPLC (MS: Waters SQD; ELSD: Waters 2424; Waters PDA; Waters Binary solvent manager; Waters sample manager). HPLC chromatographic separations were conducted using a Waters XBridge C_18_ column (2.1 mm × 50 mm, 3.5 μm particle size) or Waters XSelect column (2.1 mm × 30 mm, 2.5 μm particle size), eluting with a gradient of 5–95% acetonitrile/water +0.1% ammonia or +0.1% formic acid, or a Waters Acquity BEH C_18_ column (3 mm × 50 mm, 1.7 μm particle size) eluting with a gradient of 5–95% acetonitrile/water +0.1% formic acid. All intermediates had a measured purity ≥90% and all assay compounds had a measured purity of ≥95% as determined using analytical LC-MS (TIC and UV). The synthesis of all intermediates, and spectral data for final compounds, is included in the Supporting Information.

#### 1-Benzyl-*N*-(3-(cyclopropylcarbamoyl)phenyl)-6-oxo-1,6-dihydropyridazine-3-carboxamide (**5**)

To **29a** (100 mg, 0.43 mmol) dissolved in DCM (2 mL) was added T3P (0.282 mL, 0.48 mmol, 50 wt % in EtOAc) and DIPEA (0.12 mL, 0.87 mmol). After 10 min, **31** (92.38 mg, 0.43 mmol) was added, and the reaction left to stir at RT overnight. The reaction mixture was partitioned between DCM/water, the organic layer was separated, and the solvent was removed *in vacuo*. The crude mixture was purified by flash chromatography (0–20% DCM in MeOH) to give **5** (60 mg, 34%) as a white solid. ^1^H NMR (500 MHz, DMSO-*d*_6_) δ 10.40 (s, 1H), 8.42 (d, *J* = 4.1 Hz, 1H), 8.16 (t, *J* = 1.8 Hz, 1H), 7.98 (d, *J* = 9.7 Hz, 2H), 7.57 (d, *J* = 7.8 Hz, 1H), 7.44 (m, 3H), 7.37 (m, 2H), 7.31 (m, 1H), 7.11 (d, *J* = 9.7 Hz, 1H), 5.40 (s, 2H), 2.87 (m, 1H), 0.71 (m, 2H), 0.59 (m, 2H). ^13^C NMR (125 MHz, DMSO-*d*_6_) δ 167.80, 160.62, 159.63, 138.98, 138.49, 136.50, 135.74, 130.93, 130.30, 129.00, 128.48, 128.21, 123.69, 123.16, 120.56, 55.87, 23.56, 6.19. One quaternary carbon peak is absent. HRMS (ES^+^): *m*/*z* [M + H]^+^ calcd for C_22_H_21_N_4_O_3_, 389.1608; found 389.1617.

#### 1-Benzyl-*N*-(3-(cyclopropylcarbamoyl)phenyl)-*N*-methyl-6-oxo-1,6-dihydropyridazine-3-carboxamide (**6**)

**6** was synthesized from **38** and cyclopropylamine by an analogous method to **5**, with the initial additions carried out at 0 °C and using sat. NaHCO_3_ rather than water for the work-up, to yield an off-white solid (92 mg, 45%). ^1^H NMR (500 MHz, DMSO-*d*_6_) δ 8.45 (d, *J* = 4.0 Hz, 1H), 7.79 (m, 1H), 7.75 (m, 1H), 7.68 (d, *J* = 9.6 Hz, 1H), 7.37 (m, 2H), 7.23 (m, 3H), 6.95 (d, *J* = 9.6 Hz, 1H), 6.79 (bs, 2H), 4.88 (bs, 2H), 3.40 (s, 3H), 2.84 (m, 1H), 0.70 (m, 2H), 0.55 (m, 2H). ^13^C NMR (125 MHz, DMSO-*d*_6_) δ 166.65, 164.30, 158.74, 144.35, 141.28, 136.19, 135.72, 132.96, 129.99, 129.94, 129.60, 128.77, 128.02, 127.94, 126.15, 125.70, 55.43, 23.54, 6.21. One quaternary carbon peak is absent. HRMS (ES+): *m/z* [M + H]^+^ calcd for C_23_H_23_N_4_O_3_, 403.1765; found, 403.1764.

#### 1-Benzyl-*N*-(3-(cyclopropyl(methyl)carbamoyl)phenyl)-6-oxo-1,6-dihydropyridazine-3-carboxamide (**7**)

**7** was synthesized from **39a** and *N*-methylcyclopropylamine by an analogous method to **5**, using sat. NaHCO_3_ rather than water for the work-up, to yield an off-white solid (0.050 g, 22%). ^1^H NMR (500 MHz, DMSO-*d*_6_) δ 10.40 (s, 1H), 7.97 (d, *J* = 9.7 Hz, 1H), 7.91 (s, 1H), 7.87 (d, *J* = 8.1 Hz, 1H), 7.43 (m, 3H), 7.37 (t, *J* = 7.4 Hz, 2H), 7.32 (m, 1H), 7.28 (m, 1H), 7.11 (d, *J* = 9.7 Hz, 1H), 5.39 (s, 2H), 2.98 (bs, 2H), 2.90 (bs, 1H), 0.55 (bs, 2H), 0.44 (bs, 2H). ^13^C NMR (125 MHz, DMSO-*d*_6_) δ 160.11, 159.63, 138.74, 138.14, 136.48, 130.99, 130.30, 129.02, 128.78, 128.46, 128.21, 123.33, 121.84, 119.93, 55.88. HRMS (ES+): *m/z* [M + H]^+^ calcd for C_23_H_23_N_4_O_3_, 403.1765; found, 403.1772.

#### 1-Benzyl-*N*-(3-(cyclopropylcarbamoyl)phenyl)-6-oxo-1,6-dihydropyridine-3-carboxamide (**8**)

**8** was synthesized from commercially available 1-benzyl-6-oxo-1,6-dihydropyridine-3-carboxylic acid and **31** by an analogous method to **5**, running at 0 °C rather than RT, to yield a pale yellow solid (40 mg, 23%). ^1^H NMR (500 MHz, DMSO-*d*_6_) δ 10.14 (s, 1H), 8.66 (d, *J* = 2.1 Hz, 1H), 8.45 (d, *J* = 3.7 Hz, 1H), 8.05 (m, 2H), 7.91 (d, *J* = 7.9 Hz, 1H), 7.53 (d, *J* = 7.6 Hz, 1H), 7.43–7.30 (m, 6H), 6.54 (d, *J* = 9.5 Hz, 1H), 5.21 (s, 2H), 2.86 (m, 1H), 0.70 (m, 2H), 0.58 (m, 2H). ^13^C NMR (125 MHz, DMSO-*d*_6_) δ 167.83, 162.90, 161.76, 142.63, 139.42, 138.71, 137.26, 135.53, 129.11, 128.94, 128.16, 123.34, 122.51, 120.16, 119.20, 113.39, 52.49, 23.56, 6.21. One quaternary carbon peak is absent. HRMS (ES+): *m/z* [M + H]^+^ calcd for C_23_H_22_N_3_O_3_, 388.1656; found, 388.1653.

#### 1-Benzyl-*N*-(3-(cyclopropylcarbamoyl)-4-fluorophenyl)-6-oxo-1,6-dihydropyridazine-3-carboxamide (**9**)

**9** was synthesized from **39b** and cyclopropylamine by an analogous method to **5** to yield an off-white solid (50 mg, 90%). ^1^H NMR (500 MHz, DMSO-*d*_6_) δ 10.48 (s, 1H), 8.42 (s, 1H), 7.96 (m, 1H), 7.42 (m, 1H), 7.37 (t, *J* = 7.4 Hz, 1H), 7.31 (m, 1H), 7.11 (d, *J* = 9.7 Hz, 1H) 5.38 (s, 2H), 2.85 (m, *J* = 3.8 Hz, 1H), 0.71 (m, 2H), 0.55 (m, 2H). 13C NMR (125 MHz, DMSO-*d*_6_) 165.11, 160.57, 159.62, 156.65, 154.69, 138.56, 136.46, 134.74, 130.64 (d, *J*_CF_ = 80.1 Hz), 129.02, 128.45, 128.23, 124.91 (d, *J*_CF_ = 16.8 Hz), 124.48 (d, *J*_CF_ = 8.3 Hz), 122.35, 116.73 (d, *J*_CF_ = 23.6 Hz), 55.90, 23.44, 6.25. HRMS (ES+): *m/z* [M + H]^+^ calcd for C_22_H_20_FN_4_O_3_, 407.1514; found, 407.1499.

#### 1-Benzyl-*N*-(5-(cyclopropylcarbamoyl)-2-fluorophenyl)-6-oxo-1,6-dihydropyridazine-3-carboxamide (**10**)

**10** was synthesized from **39c** and cyclopropylamine by an analogous method to **5** to yield a white solid (32 mg, 69%).

^1^H NMR (500 MHz, DMSO-*d*_6_) δ 10.23 (s, 1H), 8.52 (d, *J* = 4.1 Hz, 1H), 8.17 (dd, *J* = 7.4, 2.0 Hz, 1H), 7.96 (d, *J* = 9.7 Hz, 1H), 7.77 (m, 1H), 7.45–7.31 (m, 6H), 7.12 (d, *J* = 9.7 Hz, 1H), 5.36 (s, 2H), 2.86 (m, 1H), 0.70 (m, 2H), 0.58 (m, 2H). ^13^C NMR (125 MHz, DMSO-*d*_6_) δ 166.49, 160.58, 159.65, 158.52, 156.52, 138.04, 136.38, 131.32, 130.59 (d, *J* = 48.9 Hz), 128.86 (d, *J* = 43.5 Hz), 128.32, 126.64 (d, *J* = 8.4 Hz), 126.32, 125.21 (d, *J* = 12.6 Hz), 116.14 (d, *J* = 20.5 Hz), 55.79, 23.60, 6.18. One quaternary carbon peak is absent. HRMS (ES+): *m/z* [M + H]^+^ calcd for C_22_H_20_FN_4_O_3_, 407.1514; found, 407.1510.

#### 1-(Cyclohexylmethyl)-*N*-(3-(cyclopropylcarbamoyl)-4-fluorophenyl)-6-oxo-1,6-dihydropyridazine-3-carboxamide (**11**)

To trimethylaluminum (2M in toluene) (0.659 mL, 1.32 mmol) was added a mixture of **28b** (110 mg, 0.44 mmol) and **33** (102 mg, 0.53 mmol) in toluene (3.5 mL), and the resulting mixture was stirred at RT in a sealed tube overnight (16 h). The resulting yellow suspension was cooled to 0 °C, and MeOH was added dropwise to the mixture until gas evolution stopped. The crude residue was adsorbed onto silica gel (1 g), then purified by flash chromatography (0–60% EtOAc in cyclohexane) to give **11** as a pale yellow solid, (123 mg, 23%). ^1^H NMR (500 MHz, DMSO-*d*_6_) δ 10.35 (s, 1H), 8.41 (d, *J* = 3.9 Hz, 1H), 7.95 (m, 2H), 7.92 (d, *J* = 9.7 Hz, 1H), 7.30 (m, 1H), 7.07 (d, *J* = 9.7 Hz, 1H), 4.06 (d, *J* = 7.4 Hz, 2H), 2.85 (m, 1H), 2.01 (m, 1H), 1.69 (m, 2H), 1.59 (m, 3H), 1.19 (m, 3H), 1.06 (m, 2H), 0.71 (m, 2H), 0.55 (m, 2H). ^13^C NMR (125 MHz, DMSO-*d*_6_) δ 165.11, 160.73, 160.08, 156.64, 154.68, 138.16, 134.72, 130.12 (d, *J*_CF_ = 82.76 Hz), 124.87 (d, *J*_CF_ = 16.5 Hz), 124.59 (d, *J*_CF_ = 7.9 Hz), 122.48, 116.68 (d, *J*_CF_ = 23.6 Hz), 57.47, 36.66, 30.32, 26.36, 25.59, 23.44, 6.25. HRMS (ES+): *m/z* [M + H]^+^ calcd for C_22_H_26_FN_4_O_3_, 413.1983; found, 413.1988.

#### *N*-[3-(Cyclopropylcarbamoyl)-4-fluorophenyl]-1-[(4-methoxyphenyl)methyl]-6-oxo-pyridazine-3-carboxamide (**12**)

**12 w**as synthesized from **29c** and **33** by an analogous method to **5** to yield a white solid (40 mg, 46%). ^1^H NMR (500 MHz, CDCl_3_) δ 10.47 (s, 1H), 8.42 (d, *J* = 3.9 Hz, 1H), 7.95 (m, 3H), 7.41 (d, *J* = 8.7 Hz, 2H), 7.31 (m, 1H), 7.08 (d, *J* = 9.7 Hz, 1H), 6.92 (d, *J* = 8.7 Hz, 2H), 5.30 (s, 2H), 3.73 (s, 3H), 2.85 (m, 1H), 0.71 (m, 2H), 0.55 (m, 2H). ^13^C NMR (125 MHz, DMSO-*d*_6_) 165.11, 160.57, 159.47 (d, *J*_CF_ = 15.1 Hz), 156.65, 154.69, 138.36, 134.72, 130.82, 130.26, 128.39, 124.92 (d, *J*_CF_ = 16.8 Hz), 124.51 (d, *J*_CF_ = 8.1 Hz), 122.40, 116.73 (d, *J*_CF_ 23.5 Hz), 114.37, 55.45 (d, *J*_CF_ = 28.1), 23.45, 6.26. Two quaternary carbon peaks are absent. HRMS (ES+): *m/z* [M + H]^+^ calcd for C_23_H_22_FN_4_O_4_, 437.1620; found, 437.1611.

#### *N*-(3-(Cyclopropylcarbamoyl)-4-fluorophenyl)-1-(3-methoxybenzyl)-6-oxo-1,6-dihydropyridazine-3-carboxamide (**13**)

To a solution of **29d** (147 mg, 0.57 mmol), HATU (255 mg, 0.67 mmol), and DIPEA (0.270 mL, 1.545 mmol) in DMF (5 mL) was added **33** (100 mg, 0.515 mmol) and stirred for 16 h. The RM was added to ice-cold water, and the resulting solid was collected, washed with cold EtOAc, then cold 50% EtOAc in pet ether, and then pentane, and dried to give **13** (105 mg, 46%). ^1^H NMR (500 MHz, DMSO-*d*_6_) δ 12.72 (s, 1H), 8.83 (d, *J* = 4.1 Hz, 1H), 8.62 (dd, *J* = 9.2, 5.3 Hz, 1H), 7.96 (d, *J* = 9.7 Hz, 1H), 7.64 (dd, *J* = 9.6, 2.8 Hz, 1H), 7.46 (m, 1H), 7.28 (t, *J* = 7.9 Hz, 1H), 7.20 (d, *J* = 7.6 Hz, 1H), 7.17 (s, 1H), 7.12 (d, *J* = 9.7 Hz, 1H), 6.90 (dd, *J* = 8.1, 1.9 Hz, 1H), 5.29 (s, 2H), 3.74 (s, 3H), 3.01 (m, 1H), 0.76 (m, 2H), 0.67 (m, 2H). ^13^C NMR (125 MHz, DMSO-*d*_6_) δ 168.59, 159.84 (d, *J*_CF_ = 7.9 Hz), 159.55, 158.53, 156.62, 138.37, 137.69, 135.07, 130.49 (d, *J*_CF_ = 10.5 Hz), 130.20, 123.06 (d, *J*_CF_ = 6.2 Hz), 122.43 (d, *J*_CF_ = 7.3 Hz), 121.54, 119.23 (d, *J*_CF_ = 22.0 Hz), 115.55 (d, *J*_CF_ = 24.0 Hz), 115.15, 113.85, 55.70 (d, *J*_CF_ = 44.2 Hz), 23.62, 6.06. Two quaternary carbon peaks are absent. HRMS (ES+): *m/z* [M + H]^+^ calcd for C_23_H_22_FN_4_O_4_, 437.1620; found, 437.1621.

#### 1-[(3-Chloro-4-methoxy-phenyl)methyl]-*N*-[3-(cyclopropylcarbamoyl)-4-fluorophenyl]-6-oxo-pyridazine-3-carboxamide (**14**)

**14** was synthesized from **29e** and **33** by an analogous method to **5** to yield a white solid (188 mg, 29%). ^1^H NMR (500 MHz, DMSO-*d*_6_) δ 10.47 (s, 1H), 8.42 (d, *J* = 3.7 Hz, 1H), 7.95 (m, 3H), 7.57 (d, *J* = 1.9 Hz, 1H), 7.43 (dd, *J* = 8.5, 1.8 Hz, 1H), 7.32 (m, 1H), 7.14 (d, *J* = 8.5 Hz, 1H), 7.09 (d, *J* = 9.7 Hz, 1H), 5.29 (s, 2H), 3.84 (s, 3H), 2.85 (m, 1H), 0.71 (m, 2H), 0.55 (m, 2H). ^13^C NMR (125 MHz, DMSO-*d*_6_), δ 165.10, 160.50, 159.56, 154.68, 138.51, 134.71, 130.94, 130.42, 130.33, 129.48, 129.10, 124.58, 122.42, 121.27, 116.85, 116.66, 113.28, 56.61, 54.94, 23.45, 6.26. HRMS (ES+): *m/z* [M + H]^+^ calcd for C_23_H_21_ClFN_4_O_4_, 471.1230; found, 471.1239.

#### *N*-[3-(Cyclopropylcarbamoyl)-4-fluorophenyl]-1-[(3-fluoro-4-methoxy-phenyl)methyl]-6-oxo-pyridazine-3-carboxamide (**15**)

**15** was synthesized from **39d** and cyclopropylamine by an analogous method to **5** to yield a pale yellow solid (650 mg, 63%). ^1^H NMR (400 MHz, DMSO-*d*_6_) δ 10.45 (s, 1H), 8.42 (d, *J* = 3.8 Hz, 1H), 7.96 (m, 3H), 7.33 (m, 2H), 7.24 (d, *J* = 8.3 HZ, 1H), 7.16 (t, *J* = 8.7 Hz, 1H), 7.09 (d, *J* = 9.7 Hz, 1H), 5.29 (s, 2H), 3.82 (s, 2H), 2.85 (m, 1H), 0.71 (m, 2H), 0.55 (m, 2H). ^13^C NMR (125 MHz, DMSO-*d*_6_) δ 165.10, 160.52, 159.55, 156.67, 154.71, 150.66, 147.30 (d, *J*_CF_ = 10.6 Hz), 138.53, 134.70, 130.62 (d, *J*_CF_ = 76.8 Hz), 129.08 (d, *J*_CF_ = 6.8 Hz), 125.38 (d, *J*_CF_ = 3.1 Hz), 124.91 (d, *J*_CF_ = 16.9 Hz), 124.55 (d, *J*_CF_ = 7.8 Hz), 122.41, 116.75 (d, *J*_CF_ = 23.7 Hz), 116.48 (d, *J*_CF_ = 18.5 Hz), 114.28, 56.48, 55.04, 23.45, 6.25. HRMS (ES+): *m/z* [M + H]^+^ calcd for C_23_H_21_F_2_N_4_O_4_, 455.1525; found, 455.1533.

#### 1-[(3-Cyano-4-methoxy-phenyl)methyl]-*N*-[3-(cyclopropylcarbamoyl)-4-fluorophenyl]-6-oxo-pyridazine-3-carboxamide (**16**)

**16** was synthesized from **29g** and **33** by an analogous method to **5** to yield a white solid (115 mg, 78%). ^1^H NMR (500 MHz, DMSO-*d*_6_) δ 10.44 (s, 1H), 8.42 (d, *J* = 3.8 Hz, 1H), 7.96 (m, 3H), 7.85 (d, *J* = 2.1 Hz, 1H), 7.79 (dd, *J* = 8.8, 2.2 Hz, 1H), 7.32 (m, 1H), 7.26 (d, *J* = 8.8 Hz, 1H), 7.10 (d, *J* = 9.7 Hz, 1H), 5.32 (s, 2H), 3.91 (s, 3H), 2.85 (m, 1H), 0.71 (m, 2H), 0.55 (m, 2H). ^13^C NMR (125 MHz, DMSO-*d*_6_) δ 165.09, 160.99, 160.51, 159.59, 138.67, 135.90, 134.68, 134.04, 131.01, 130.38, 129.13, 124.57 (d, *J*_CF_ = 7.3 Hz), 122.43, 116.78 (d, *J*_CF_ = 20.7 Hz), 112.93, 100.66, 56.91, 54.80, 23.45, 6.26. Three quaternary carbon peaks are absent. HRMS (ES+): *m/z* [M + H]^+^ calcd for C_24_H_21_FN_5_O_4_, 462.1572; found, 462.1577.

#### 1-[(4-Chloro-3-fluorophenyl)methyl]-*N*-[3-(cyclopropylcarbamoyl)-4-fluorophenyl]-6-oxo-pyridazine-3-carboxamide (**17**)

**17** was synthesized from **29h** and **33** by an analogous method to **5** to yield a yellow solid (67 mg, 57%). ^1^H NMR (500 MHz, DMSO-*d*_6_) δ 10.45 (s, 1H), 8.42 (d, *J* = 3.9 Hz, 1H), 7.96 (m, 3H), 7.60 (t, 8.05 Hz, 1H), 7.52 (dd, *J* = 10.2, 1.8 Hz, 1H), 7.30 (m, 2H), 7.12 (d, 9.7 Hz, 1H), 5.36 (s, 2H), 2.85 (m, 1H), 0.71 (m, 2H), 0.55 (m, 2H). ^13^C NMR (125 MHz, DMSO-*d*_6_) δ 165.08, 160.49, 159.62, 158.77, 138.85, 138.80 (d, *J*_CF_ = 7.1 Hz), 134.70 (d, *J*_CF_ = 2.4), 131.17 (d, *J*_CF_ = 12.4 Hz), 130.43, 125.85 (d, *J*_CF_ = 3.4 Hz), 124.91 (d, *J*_CF_ = 17.4 Hz), 124.50 (d, *J*_CF_ = 8.3 Hz), 122.37, 117.08 (d, *J*_CF_ = 21.6 Hz), 116.76 (d, *J*_CF_ = 24.2 Hz), 55.06, 23.45, 6.26. Three quaternary carbon peaks are absent. HRMS (ES+): *m/z* [M + H]^+^ calcd for C_22_H_18_ClF_2_N_4_O_3_, 459.1030; found, 459.1019.

#### *N*-(3-(Cyclopropylcarbamoyl)-4-fluorophenyl)-1-((5-fluoro-6-methoxypyridin-3-yl)methyl)-6-oxo-1,6-dihydropyridazine-3-carboxamide (**18**)

**18** was synthesized from **29i** and **33** by an analogous method to **13** and purified by flash chromatography (50% EtOAc in pet. Ether). The resulting solid was further purified by precipitation from MeOH/THF and washing with pentane to yield a white solid (50 mg, 6%). ^1^H NMR (500 MHz, DMSO-*d*_6_) δ 12.81 (s, 1H), 8.88 (s, 1H), 8.60 (m, 1H), 8.30 (s, 1H), 8.00 (d, *J* = 10.8 Hz, 1H), 7.94 (d, *J* = 9.6 Hz, 1H), 7.66 (d, *J* = 7.3 Hz, 1H), 7.46 (t, *J* = 6.9 Hz, 1H), 7.11 (d, *J* = 9.6 Hz, 1H), 5.30 (s, 2H), 3.91 (s, 3H), 3.06 (m, 1H), 0.78 (d, *J* = 5.2 Hz, 2H), 0.68 (s, 2H). ^13^C NMR (125 MHz, DMSO-*d*_6_) δ 168.71, 159.64 (d, *J*_CF_ = 26.5 Hz), 158.54, 156.84, 152.90, 147.92, 145.93, 142.79 (d, *J*_CF_ = 4.8 Hz), 138.67, 135.12, 130.52, 125.91, 125.00 (d, *J*_CF_ = 15.9 Hz), 122.81 (d, *J*_CF_ = 6.5 Hz), 122.35 (d, *J*_CF_ = 7.3 Hz), 119.33 (d, *J*_CF_ = 21.9 Hz), 115.58 (d, *J*_CF_ = 24.2 Hz), 54.06, 52.19, 23.60, 6.03. HRMS (ES+): *m/z* [M + H]^+^ calcd for C_22_H_20_F_2_N_5_O_4_, 456.1478; found, 456.1490.

#### *N*-(4-Fluoro-3-((*trans-*3-methoxycyclobutyl)carbamoyl)phenyl)-1-(4-methoxybenzyl)-6-oxo-1,6-dihydropyridazine-3-carboxamide (**19**)

**19** was synthesized from **28c** and **34** by an analogous method to **11**, with purification by flash chromatography (0–100% EtOAc in cyclohexane) and subsequent trituration with ether yielding a white solid (250 mg, 95%). ^1^H NMR (500 MHz, DMSO-*d*_6_) δ 10.43 (s, 1H), 8.64 (d, *J* = 6.8 Hz, 1H), 7.97 (m, 2H), 7.94 (d, *J* = 9.7 Hz, 1H), 7.41 (d, *J* = 8.7 Hz, 2H), 7.32 (t, *J* = 9.8 Hz, 1H), 7.08 (d, *J* = 9.7 Hz, 1H), 6.92 (d, *J* = 8.8 Hz, 2H), 5.31 (s, 2H), 4.41 (m, 1H), 4.00 (m, 1H), 3.74 (s, 3H), 3.16 (s, 3H), 2.26 (m, 4H). ^13^C NMR (125 MHz, DMSO-*d*_6_) δ 163.47, 160.59, 159.49 (d, *J*_*CF*_ = 11.2 Hz), 154.77, 138.39, 134.74, 130.79, 130.25, 128.42, 124.97 (d, *J*_*CF*_ = 16.2 Hz), 124.56 (d, *J*_*CF*_ = 7.8 Hz), 122.49, 116.72 (d, *J*_*CF*_ = 23.7 Hz), 114.40, 72.43, 55.59, 55.39, 55.33, 41.91, 36.32. Two quaternary carbon peaks are absent. HRMS (ES+): *m/z* [M + H]^+^ calcd for C25H26FN4O5, 481.1882; found, 481.1880.

#### *N*-(4-Fluoro-3-((*trans*-3-methoxycyclobutyl)carbamoyl)phenyl)-1-(3-fluoro-4-methoxybenzyl)-6-oxo-1,6-dihydropyridazine-3-carboxamide (**20**)

**20** was synthesized from **39d** and *trans-*3-methoxycyclobutylamine by an analogous method to **5** to yield a white solid (33 mg, 55%). ^1^H NMR (500 MHz, DMSO-*d*_6_) δ 10.42 (s, 1H), 8.64 (d, *J* = 6.8 Hz), 7.96 (m, 3H), 7.33 (m, 1H), 7.24 (d, *J* = 8.5 Hz), 7.15 (t, *J* = 8.7 Hz), 7.09 (d, *J* = 9.7 Hz), 5.30 (s, 2H), 4.41 (m, 1H), 4.00 (m, 1H), 3.82 (s, 3H), 3.16 (s, 3H), 2.25 (m, 4H). ^13^C NMR (125 MHz, DMSO-*d*_6_) δ 163.47, 160.54, 159.56, 154.78, 138.56, 134.72, 130.91, 130.32, 129.14 (d, *J*_*CF*_ = 6.3 Hz), 125.36 (d, *J*_*CF*_ = 3.1 Hz), 124.58, 122.51 (d, *J*_*CF*_ = 1.8 Hz), 116.74 (d, *J*_*CF*_ = 23.6 Hz), 116.49 (d, *J*_*CF*_ = 18.6 Hz), 114.38, 72.43, 56.54, 55.39, 55.03, 41.90, 36.31. Three quaternary carbon peaks are absent. HRMS (ES+): *m/z* [M + H]^+^ calcd for C_25_H_25_F_2_N_4_O_5_, 499.1788; found, 499.1783.

#### *N*-[4-Fluoro-3-(2-morpholinoethylcarbamoyl)phenyl]-1-[(4-methoxyphenyl)methyl]-6-oxo-pyridazine-3-carboxamide (**21**)

For **21**, **39e** was synthesized from **36e** by an analogous method to **29a** and used without purification. **39e** was then coupled to 4-(2-aminoethyl)morpholine by an analogous method to **5** to yield a white solid (23 mg, 34%). ^1^H NMR (500 MHz, DMSO-*d*_6_) δ 10.49 (s, 1H), 8.25 (m, 1H), 8.08 (dd, *J* = 6.4, 2.8 Hz, 1H), 7.98 (m, 1H), 7.94 (d, *J* = 9.7 Hz, 1H), 7.41 (d, *J* = 8.7 Hz, 2H), 7.34 (t, *J* = 9.6 Hz, 1H), 7.08 (d, *J* = 9.7 Hz, 1H), 6.92 (d, *J* = 8.7 Hz, 2H), 5.30 (s, 2H), 3.73 (s, 3H), 3.59 (t, *J* = 4.5 Hz, 4H), 3.40 (q, *J* = 6.4 Hz, 2H), 2.48 (t, *J* = 6.8 Hz, 2H), 2.43 (s, 4H). ^13^C NMR (125 MHz, DMSO-*d*_6_) δ 163.54, 160.60, 159.47 (d, *J*_CF_ = 15.5 Hz), 156.90, 154.94, 138.38, 134.89, 130.82, 130.27, 128.40, 124.85 (d, *J*_CF_ = 8.3 Hz), 124.20 (d, *J*_CF_ = 15.6 Hz), 122.78, 116.83 (d, *J*_CF_ = 24.6 Hz), 114.36, 66.72, 57.41, 55.56, 55.34, 53.67, 37.03. One quaternary carbon peak absent. HRMS (ES+): *m/z* [M + H]^+^ calcd for C_26_H_29_FN_5_O_5_, 510.2147; found, 510.2158.

#### 1-[(3-Fluoro-4-methoxy-phenyl)methyl]-*N*-[4-fluoro-3-(2-morpholinoethylcarbamoyl)phenyl]-6-oxo-pyridazine-3-carboxamide (**22**)

**22** was synthesized from **39d** 4-(2-aminoethyl)morpholine by an analogous method to **5** to yield an off-white solid (600 mg, 38%). ^1^H NMR (500 MHz, DMSO-*d*_6_) δ 10.48 (s, 1H), 8.25 (m, 1H), 8.08 (dd, *J* = 6.4, 2.8 Hz, 1H), 7.98 (m, 1H), 7.95 (d, *J* = 9.7 Hz, 1H), 7.35 (m, 2H), 7.25 (d, *J* = 8.5 Hz, 1H), 7.15 (t, *J* = 8.7 Hz, 1H), 7.09 (d, *J* = 9.7 Hz, 1H), 5.29 (s, 2H), 3.82 (s, 3H), 3.59 (t, *J* = 4.5 Hz, 4H), 3.40 (q, *J* = 6.4 Hz, 2H), 2.48 (t, *J* = 6.8 Hz, 2H), 2.43 (s, 4H). ^13^C NMR (125 MHz, DMSO-*d*_6_) δ 163.53, 160.55, 159.55, 156.91, 154.96, 152.60, 147.30 (d, *J*_CF_ = 10.5 Hz), 138.55, 134.86, 130.63 (d, *J*_CF_ = 79.3 Hz), 129.09 (d, *J*_CF_ = 5.9 Hz), 125.39 (d, *J*_CF_ = 2.9 Hz), 124.89 (d, *J*_CF_ = 8.1 Hz), 124.21 (d, *J*_CF_ = 15.8 Hz), 122.81, 116.86 (d, *J*_CF_ = 23.7 Hz), 116.50 (d, *J*_CF_ = 18.8 Hz), 114.27, 66.72, 57.41, 56.48, 55.04, 53.67, 37.03. HRMS (ES+): *m/z* [M + H]^+^ calcd for C_26_H_28_F_2_N_5_O_5_, 528.2053; found, 528.2073.

#### *N*-(4-Fluoro-3-((*trans*-3-morpholinocyclobutyl)carbamoyl)phenyl)-1-(3-fluoro-4-methoxybenzyl)-6-oxo-1,6-dihydropyridazine-3-carboxamide (**23**) and *N*-(4-Fluoro-3-((*cis*-3-morpholinocyclobutyl)carbamoyl)phenyl)-1-(3-fluoro-4-methoxybenzyl)-6-oxo-1,6-dihydropyridazine-3-carboxamide (**24**)

**23** and **24** were synthesized from **39d** (Li salt) and 3-morpholinocyclobutylamine by an analogous procedure to **13**. The product was obtained as a mixture of two isomers (cis/trans), which were separated by flash chromatography (0–15% DCM in MeOH), to give the separated isomers. Each isomer was further purified using preparatory HPLC to give the corresponding products as white solids.

**23** (40 mg, 7%, trans). ^1^H NMR (500 MHz, DMSO-*d*_6_) δ 10.46 (s, 1H), 8.71 (d, *J* = 6.9 Hz, 1H), 7.96 (m, 3H), 7.34 (m, 2H), 7.24 (d, *J* = 8.5 Hz, 1H), 7.15 (t, *J* = 8.7 Hz, 1H), 7.10 (d, *J* = 9.7 Hz, 1H), 5.29 (s, 2H), 4.33 (m, 1H), 3.82 (s, 3H), 3.60 (m, 4H), 2.82 (m, 1H), 2.32–2.22 (m, 6H), 2.07 (m, 2H). ^13^C NMR (125 MHz, DMSO-*d*_6_) δ 163.46, 160.04 (d, *J*_*CF*_ = 122.77 Hz), 156.73, 154.76, 152.61, 150.66, 147.30 (d, *J*_*CF*_ = 10.6 Hz), 138.53, 134.71, 130.63 (d, *J*_*CF*_ = 76.3 Hz), 129.08 (d, *J*_*CF*_ = 7.1 Hz), 125.38 (d, *J*_*CF*_ = 3.2 Hz), 125.03 (d, *J*_*CF*_ = 16.2 Hz), 124.54 (d, *J*_*CF*_ = 7.9 Hz), 122.49, 116.75 (d, *J*_*CF*_ = 23.2 Hz), 116.49 (d, *J*_*CF*_ = 18.3 Hz), 114.28, 66.59, 56.87, 56.48, 50.54, 42.28, 32.92. HRMS (ES+): *m/z* [M + H]^+^ calcd for C_28_H_30_F_2_N_5_O_5_, 554.2210; found, 554.2225.

**24** (34.1 mg, 6%, cis). ^1^H NMR (500 MHz, DMSO-*d*_6_) δ 10.45 (s, 1H), 8.59 (d, *J* = 7.6 Hz, 1H), 7.96 (m, 3H), 7.34 (m, 2H), 7.24 (d, *J* = 8.7 Hz, 1H), 7.15 (t, 8.7 Hz, 1H), 7.09 (d, *J* = 9.7 Hz, 1H), 5.29 (s, 2H), 4.13 (m, 1H), 3.82 (s, 3H), 3.57 (t, *J* = 4.1 Hz, 4H), 2.43 (m, 3H), 2.25 (s, 4H), 1.84 (q, 8.6 Hz, 2H). ^13^C NMR (125 MHz, DMSO-*d*_6_) δ 168.72, 159.64 (d, *J*_*CF*_ = 26.5 Hz), 158.54, 156.84, 152.90, 147.88, 145.82, 142.79 (d, *J*_*CF*_ = 4.8 Hz), 138.67, 135.12, 130.52, 125.91, 125.00 (d, *J*_*CF*_ = 15.9 Hz), 122.81 (d, *J*_*CF*_ = 6.5 Hz), 122.35 (d, *J*_*CF*_ = 7.3 Hz), 119.33 (d, *J*_*CF*_ = 21.9 Hz), 115.57 (d, *J*_*CF*_ = 24.2 Hz), 54.06, 52.19, 23.60, 6.03. Some quaternary peaks are absent, aliphatic peaks may be weak or obscured by DMSO signal. HRMS (ES+): *m/z* [M + H]^+^ calcd for C_28_H_30_F_2_N_5_O_5_, 554.2210; found, 554.2225.

#### (*R*)-*N*-(4-Fluoro-3-((1-hydroxypropan-2-yl)carbamoyl)phenyl)-1-(3-fluoro-4-methoxybenzyl)-6-oxo-1,6-dihydropyridazine-3-carboxamide (**25**)

**25** was synthesized from **39d** and (*R*)-2-aminopropan-1-ol by an analogous method to **5** to yield a white solid (43 mg, 54%). ^1^H NMR (500 MHz, DMSO-*d*_6_) δ 10.47 (s, 1H), 7.99 (m, 4H), 7.33 (m, 2H), 7.25 (d, *J* = 8.5 Hz, 1H), 7.15 (t, *J* = 8.7 Hz, 1H), 7.09 (d, *J* = 9.7 Hz, 1H), 5.29 (s, 2H), 4.79 (bs, 1H), 4.00 (m, 1H), 3.82 (s, 3H), 3.47 (dd, *J* = 10.5, 5.4 Hz, 1H), 3.34 (dd, *J* = 10.5, 6.3 Hz, 2H), 1.14 (d, *J* = 6.7 Hz, 3H). ^13^C NMR (125 MHz, DMSO-*d*_6_) δ 163.29, 160.04 (d, *J*_*CF*_ = 123.6 Hz), 156.79, 154.84, 152.60, 150.66, 147.30 (d, *J*_*CF*_ = 10.5 Hz), 138.55, 134.71, 130.63 (d, *J*_*CF*_ = 78.5 Hz), 129.08 (d, *J*_*CF*_ = 6.3 Hz), 125.37 (d, *J*_*CF*_ = 3.1 Hz), 124.83 (d, *J*_*CF*_ = 16.7 Hz), 124.65 (d, *J*_*CF*_ = 7.8 Hz), 122.71, 116.73 (d, *J*_*CF*_ = 23.9 Hz), 116.49 (d, *J*_*CF*_ = 18.5 Hz), 114.27, 64.63, 56.48, 55.04, 47.76, 17.51. HRMS (ES+): *m/z* [M + H]^+^ calcd for C_23_H_23_F_2_N_4_O_5_, 473.1631; found, 473.1630.

#### *N*-(4-Fluoro-3-(methylcarbamoyl)phenyl)-1-(4-methoxybenzyl)-6-oxo-1,6-dihydropyridazine-3-carboxamide (**26**)

**26** was synthesized from **29c** and **35** by an analogous method to **13**, purifying by flash chromatography (0–2% MeOH in DCM) and subsequent trituration with MeOH to yield a pale yellow solid (5.46 g, 46%). ^1^H NMR (500 MHz, DMSO-*d*_6_) δ 10.48 (s, 1H), 8.28 (s, 1H), 8.07 (dd, *J* = 6.4, 2.7 Hz, 1H), 7.98 (m, 1H), 7.94 (d, *J* = 9.7 Hz, 1H), 7.41 (d, *J* = 8.7 Hz, 2H), 7.33 (t, *J* = 9.5 Hz, 1H), 7.08 (d, *J* = 9.7 Hz, 1H), 6.92 (d, *J* = 8.7 Hz, 2H), 5.30 (s, 2H), 3.73 (s, 3H), 2.80 (d, *J* = 4.6 Hz, 3H). ^13^C NMR (125 MHz, DMSO-*d*_6_) δ 164.13, 160.59, 159.47 (d, *J*_CF_ = 15.5 Hz), 156.83, 154.87, 138.37, 134.85 (d, *J*_CF_ = 2.3 Hz), 130.82, 130.28, 128.39, 124.74 (d, *J*_CF_ = 8.4 Hz), 124.31 (d, *J*_CF_ = 15.9 Hz), 122.73 (d, *J*_CF_ = 2.3 Hz), 116.77 (d, *J*_CF_ = 24.3 Hz), 114.36, 55.55, 55.34, 26.81. One quaternary carbon peak absent. HRMS (ES+): *m/z* [M + H]^+^ calcd for C_21_H_20_FN_4_O_4_, 411.1463; found, 411.1467.

#### 1-[(3-Fluoro-4-methoxy-phenyl)methyl]-*N*-[4-fluoro-3-(methylcarbamoyl)phenyl]-6-oxo-pyridazine-3-carboxamide (**27**)

**27** was synthesized from **39d** methylamine by an analogous method to **5** to yield a white solid (200 mg, 19%). ^1^H NMR (500 MHz, DMSO-*d*_6_) δ 10.47 (s, 1H), 8.78 (s, 1H), 8.06 (dd, *J* = 6.4, 2.8 Hz, 1H), 7.98 (m, 1H), 7.95 (d, *J* = 9.7 Hz, 1H), 7.35 (m, 1H), 7.25 (d, *J* = 8.5 Hz, 1H), 7.15 (t, *J* = 8.7 Hz, 1H), 7.09 (d, *J* = 9.7 Hz, 1H), 5.29 (s, 1H), 3.82 (s, 1H), 2.80 (d, *J* = 4.6 Hz, 1H), 2.51 (m, 1H). ^13^C NMR (125 MHz, DMSO-*d*_6_) δ 164.11, 160.04 (d, *J*_CF_ = 123.9 Hz), 156.84, 154.89, 152.60, 150.66, 147.30 (d, *J*_CF_ = 10.9 Hz), 138.54, 134.81, 130.63 (d, *J*_CF_ = 78.4 Hz), 129.08 (d, *J*_CF_ = 6.1 Hz), 125.39 (d, *J*_CF_ = 3.1 Hz), 124.78 (d, *J*_CF_ = 8.3 Hz), 124.32 (d, *J*_CF_ = 15.8 Hz), 122.76 (d, *J*_CF_ = 2.2 Hz), 116.82 (d, *J*_CF_ = 24.1 Hz), 116.50 (d, *J*_CF_ = 18.4 Hz), 114.28, 56.48, 55.04, 26.81. HRMS (ES+): *m/z* [M + H]^+^ calcd for C_21_H_19_F_2_N_4_O_4_, 429.1369; found, 429.1371.

## Data Availability

CryoEM structure of **5** PDB 8OLU. The authors will release the atomic coordinates upon article publication.

## Supporting Information Available

Synthesis of intermediates; ^1^H, ^13^C NMR traces and HPLC traces of final compounds; in vitro assays; in vivo pharmacokinetics; cryo-EM Methods; and computational methods (PDF)
Molecular formula strings (CSV)
Proteasome and antiparasitic data for compounds **4**–**27** (XLSX)
Docking model of **5** bound to *T. cruzi* homology model (PDB)
Docking model of **15** bound to *T. cruzi* homology model (PDB)

## Supplementary Material

jm3c00582_si_003.xlsx

jm3c00582_si_004.pdb

jm3c00582_si_005.pdb

## Notes

All regulated procedures on living animals in Dundee were carried out under the authority of a project license issued by the Home Office under the Animals (Scientific Procedures) Act 1986, as amended in 2012 (and in compliance with EU Directive EU/2010/63). License applications have been approved by the University’s Ethical Review Committee (ERC) before submission to the Home Office. The ERC has a general remit to develop and oversee policy on all aspects of the use of animals on university premises and is a subcommittee of the University Court, its highest governing body. All animal studies carried out by GSK were in accordance with Animals (Scientific Procedures) Act 1986 and the GSK Policy on the Care, Welfare, and Treatment of Laboratory Animals.

## Abbreviations Used

BENDITA: Benznidazole New Doses Improved Treatment and Associations
BNZ: benznidazole
ER: efflux ratio
FMO: frontier molecular orbital
MDCK: Madin Darby canine kidney
MDR1: multidrug resistance mutation
MP2: second-order Møller–Plesset perturbation theory
PIEDA: pair interaction energy decomposition analysis
T3P: propanephosphonic acid anhydride
VL: visceral leishmaniasis
