# Supplementary material for: Structure-Guided Design and Synthesis of a Pyridazinone Series of Trypanosoma cruzi Proteasome Inhibitors
Source: J Med Chem. 2023 Jul 28;66(15):10413–31. doi: 10.1021/acs.jmedchem.3c00582 (PMC10424187; doi:10.1021/acs.jmedchem.3c00582)
Supplement: Supplementary file 1 — jm3c00582_si_001.pdf [file jm3c00582_si_001.pdf]

Supporting Information

Structure Guided Design and Synthesis of a Pyridazinone Series of *Trypanosoma cruzi* Proteasome Inhibitors.

Michael G. Thomas [a], Kate McGonagle [a], Paul Rowland [b], David A. Robinson [a], Peter G. Dodd [a], Isabel Camino-Díaz [c], Lorna Campbell [a], Juan Cantizani [d], Pablo Castañeda [c], Daniel Conn [b], Peter D. Craggs [b], Darren Edwards [a], Liam Ferguson [a], Andrew Fosberry [b], Laura Frame [a], Panchali Goswami [b], Xiao Hu [a], Justyna Korczynska [b], Lorna MacLean [a], Julio Martin [d], Nicole Mutter [a], Maria Osuna-Cabello [a], Christy Paterson [a], Imanol Peña [d], Erika G. Pinto [a], Caterina Pont [a], Jennifer Riley [a], Yoko Shishikura [a], Frederick Simeons [a], Laste Stojanovski [a], John Thomas [a], Karolina Wrobel [a], Robert J. Young [e], Filip Zmuda [a], Fabio Zuccotto [a], Kevin D. Read [a], Ian H. Gilbert [a], Maria Marco [d], Timothy J. Miles [d], Pilar Manzano [d, *], Manu De Rycker [a, *]

[a] Drug Discovery Unit, University of Dundee, School of Life Sciences, Sir James Black Centre, Dow Street, Dundee, UK DD1 5EH

[b] GlaxoSmithKline, Chemistry, Medicines Research Centre, Gunnels Wood Road, Stevenage, UK SG1 2NY

[c] GlaxoSmithKline, Discovery DMPK, IVIVT, Severo Ochoa, 2 PTM, Tres Cantos, Madrid, ES 28760

[d] GlaxoSmithKline, Global Health R&D, Severo Ochoa 2, PTM, Tres Cantos, Madrid, ES 28760

[e] Blue Burgundy Ltd, Ampthill, Bedfordshire, UK, MK45 2AD

Corresponding Authors

Manu De Rycker - Drug Discovery Unit, University of Dundee, School of Life Sciences, Sir James Black Centre, Dow Street, Dundee, UK DD1 5EH; Email: M.DeRycker@dundee.ac.uk

Supporting Information

Pilar Manzano - GlaxoSmithKline, Global Health R&D, Severo Ochoa 2, PTM, Tres Cantos, Madrid, ES 28760; Email; pilar.m.manzano@gsk.com

Contents

General Experimental Information	S-3
Synthesis of intermediates	S-4
^{1}H, ^{13}C NMR and HPLC traces of final compounds	S-13
In vitro assays	S-55
In vivo pharmacokinetics	S-61
Cryo-EM Methods	S-62
Computational Methods	S-69

General Experimental Information

Chemicals and solvents were purchased from the Aldrich Chemical Company, Fluka, ABCR, VWR, Acros, Fluorochem and Alfa Aesar and were used as received. Air- and moisture-sensitive reactions were carried out under an inert atmosphere of nitrogen in oven-dried glassware. Flash column chromatography was performed using pre-packed silica gel cartridges (230-400 mesh, 40–63 µm, from Redisep) using a Teledyne ISCO Combiflash Companion, or Combiflash Retrieve. ^{1}H NMR and ^{13}C NMR spectra were recorded on a Bruker Avance DPX 500 spectrometer (^{1}H at 500.1 MHz, ^{13}C at 125.8 MHz). Chemical shifts (δ) are expressed in ppm recorded using the residual solvent as the internal reference in all cases. Signal splitting patterns are described as singlet (s), doublet (d), triplet (t), quartet (q), multiplet (m), broad (br), or a combination thereof. Coupling constants (J) are quoted to the nearest 0.1 Hz. High resolution electrospray measurements were performed on a Bruker Daltonics MicrOTOF mass spectrometer. Low resolution electrospray (ES) mass spectra were recorded on an Advion Compact mass spectrometer (CMS: model ExpressIon CMS) connected to Dionex Ultimate 3000 UPLC system with diode array detector, or an Acquity UPLC (MS: Waters SQD; ELSD: Waters 2424; Waters PDA; Waters Binary solvent manager; Waters sample manager). HPLC chromatographic separations were conducted using a Waters XBridge C_{18} column (2.1 x 50mm, 3.5 µm particle size) or Waters XSelect column (2.1 x 30mm, 2.5 µm particle size), eluting with a gradient of 5-95% acetonitrile/water +0.1% ammonia or +0.1% formic acid, or a Waters Acquity BEH C_{18} column (3 x 50mm, 1.7 µm particle size) eluting with a gradient of 5-95% acetonitrile/water +0.1% formic acid. All intermediates had a measured purity ≥ 90% and all assay compounds had a measured purity of ≥ 95% as determined using analytical LC-MS (TIC and UV). The synthesis of all intermediates, and spectral data for final compounds, is included in the Supporting Information.

Synthesis of intermediates

Methyl-1-benzyl-6-oxo-pyridazine-3-carboxylate (28a)

To a suspension of methyl 6-oxo-1,6-dihydropyridazine-3-carboxylate (5 g, 32.44 mmol) in MeCN (300 mL) was added potassium carbonate (8.96 g, 64.89 mmol) followed by bromomethylbenzene (7.21 g, 42.18 mmol) and left to stir at RT for 24 h. The mixture was concentrated under reduced pressure then partitioned between EtOAc and water. The organic layer was separated, washed with brine, concentrated under reduced pressure, then purified by flash chromatography (0 – 60% EtOAc / Heptane) to give **28a** (6.94 g, 87%) as an off-white solid. ^{1}H NMR (500 MHz, $CDCl_3$) δ 7.84 (d, J = 9.7 Hz, 1H), 7.48 (m, 2H), 7.34 (m, 3H), 6.96 (d, J = 9.7 Hz, 1H), 5.43 (s, 2H), 3.99 (s, 3H). m/z 245.1 $[M+H]^+$

Methyl 1-(cyclohexylmethyl)-6-oxo-1,6-dihydropyridazine-3-carboxylate (28b)

28b was synthesised from methyl 6-oxo-1,6-dihydropyridazine-3-carboxylate and (bromomethyl)cyclohexane by an analogous method to **28a**, using cesium carbonate rather than potassium carbonate, to yield a white solid (140 mg, 43%). ^{1}H NMR (400 MHz, DMSO- d_6) δ ppm 7.84 (d, J=9.60 Hz, 1 H) 7.01 (d, J=9.60 Hz, 1 H) 3.98 (d, J=7.33 Hz, 2 H) 3.86 (s, 3 H) 1.84 (ttd, J=11.04, 11.04, 7.41, 7.41, 3.54 Hz, 1 H) 1.52 - 1.72 (m, 5 H) 1.08 - 1.23 (m, 3 H) 0.93 - 1.07 (m, 2 H). m/z 251 $[M+H]^+$

Methyl 1-(4-methoxybenzyl)-6-oxo-1,6-dihydropyridazine-3-carboxylate (28c)

28c Was synthesised from methyl 6-oxo-1,6-dihydropyridazine-3-carboxylate and 1-(bromomethyl)-4-methoxybenzene by an analogous method to **28a**, using cesium carbonate rather than potassium carbonate to yield a white solid (760 mg, 85%). ^{1}H NMR (500 MHz, $CDCl_3$) δ 7.83 (d, J = 9.7 Hz, 1H), 7.45 (d, J = 8.7 Hz, 2H), 6.94 (d, J = 9.7 Hz, 1H), 6.88 (d, J = 8.7 Hz, 2H), 5.36 (s, 2H), 3.99 (s, 3H), 3.81 (s, 3H). m/z 275 $[M+H]^+$.

Methyl 1-[(3-chloro-4-methoxy-phenyl)methyl]-6-oxo-pyridazine-3-carboxylate (28e)

28e was synthesised from methyl 6-oxo-1,6-dihydropyridazine-3-carboxylate and 1-(bromomethyl)-3-chloro-4-methoxybenzene by an analogous method to **28a** (1.1 g, 84%). ^{1}H NMR (500 MHz, DMSO- d_6) δ 7.89 (d, J = 9.7 Hz, 1H), 7.40 (d, J = 1.6 Hz, 1H), 7.28 (d, J = 8.6 Hz, 1H), 7.13 (d, J = 8.5 Hz, 1H), 7.07 (d, J = 9.7 Hz, 1H), 5.27 (s, 2H), 3.87 (s, 3H), 3.84 (s, 3H).

Methyl 1-[(3-fluoro-4-methoxy-phenyl)methyl]-6-oxo-pyridazine-3-carboxylate (28f)

28f was synthesised from methyl 6-oxo-1,6-dihydropyridazine-3-carboxylate and 1-(bromomethyl)-3-fluoro-4-methoxybenzene by an analogous method to **28a** (2.83 g, 75%). ^{1}H NMR (400 MHz, DMSO- d_6) δ 7.87 (d, J = 9.7 Hz, 1H), 7.19 - 7.05 (m, 1H), 5.26 (s, 2H), 3.87 (s, 3H), 3.82 (s, 3H). m/z 293 $[M+H]^+$

Methyl 1-[(3-cyano-4-methoxy-phenyl)methyl]-6-oxo-pyridazine-3-carboxylate (28g)

28g was synthesised from methyl 6-oxo-1,6-dihydropyridazine-3-carboxylate and 1-(bromomethyl)-3-cyano-4-methoxybenzene by an analogous method to **28a** (106 mg, 73%). ^{1}H NMR (400 MHz, DMSO- d_6) δ 7.87 (d, J = 9.7 Hz, 1H), 7.67 (d, J = 2.2 Hz, 1H), 7.63 (dd, J = 2.2, 9.7 Hz, 1H), 7.24 (d, J = 8.7 Hz, 1H), 7.07 (d, J = 9.7 Hz, 1H), 5.30 (s, 2H), 3.90 (s, 3H), 3.87 (s, 3H). m/z 300.2 $[M+H]^+$

1-Benzyl-6-oxo-pyridazine-3-carboxylic acid (29a)

To a solution of **28a** (6.94 g, 28.40 mmol) in THF (80 mL) was added a solution of NaOH (1.79 g, 42.60 mmol) in water (80 mL) and left to stir at RT for 2 h. The mixture was concentrated under reduced pressure to remove THF, and the remaining aqueous was acidified with 1M HCl. The resulting precipitate was collected via vacuum filtration to afford **29a** (6.31,

97% yield) as a white solid. ^{1}H NMR (400 MHz, DMSO- d_6) δ 13.60 (bs, 1H), 7.86 (d, J = 9.7 Hz, 1H), 7.32 (m, 5H), 7.05 (d, J = 9.7 Hz, 1H), 5.32 (s, 2H). m/z 231 $[M+H]^+$

Methyl 1-(4-methoxybenzyl)-6-oxo-1,6-dihydropyridazine-3-carboxylate (29c)

29c was synthesised from **28c** by an analogous method to **29a** to yield a white solid (645 mg, 89%). ^{1}H NMR (500 MHz, $CDCl_3$) δ 9.03 (bs, 1H), 7.90 (d, J = 9.7 Hz, 1H), 7.41 (d, J = 8.6 Hz, 2H), 7.04 (d, J = 9.7, 1H), 6.90 (d, J = 8.7, 2H), 5.34 (s, 2H), 3.82 (s, 3H).

1-(3-Methoxybenzyl)-6-oxo-1,6-dihydropyridazine-3-carboxylic acid (29d)

29d was synthesised in two steps from methyl 6-oxo-1,6-dihydropyridazine-3-carboxylate by analogous methods to **28a** and **29a** to yield an off-white solid (26 mg, 73%). m/z 261.0 $[M+H]^+$.

1-(3-Chloro-4-methoxybenzyl)-6-oxo-1,6-dihydropyridazine-3-carboxylic acid (29e)

29e Was synthesised from **28e** by an analogous method to **29a** (145 mg, 83%). ^{1}H NMR (400 MHz, DMSO- d_6) δ 13.60 (bs, 1H), 7.85 (d, J = 9.7 Hz, 1H), 7.41 (d, J = 2.1 Hz, 1H), 7.28 (dd, J = 2.1, 8.5 Hz, 1H), 7.13 (d, J = 8.5, 1H), 7.03 (d, J = 9.7 Hz, 1H), 5.25 (s, 2H), 3.29 (s, 3H).

1-[(3-Fluoro-4-methoxy-phenyl)methyl]-6-oxo-pyridazine-3-carboxylic acid (29f)

29f was synthesised from **28f** by an analogous method to **29a** to yield a white solid (2.50 g, 93%). ^{1}H NMR (500 MHz, DMSO- d_6) δ 13.58 (bs, 1H), 7.85 (d, J = 9.7 Hz, 1H), 7.20 – 7.09 (m, 3H), 7.03 (d, J = 9.7 Hz, 1H), 5.25 (s, 2H), 3.82 (s, 3H)

1-[(3-Cyano-4-methoxy-phenyl)methyl]-6-oxo-pyridazine-3-carboxylic acid (29g)

29g was synthesised from **28g** by an analogous method to **29a** to yield a white solid (92 mg, 91%). ^{1}H NMR (400 MHz, DMSO- d_6) δ 13.58 (bs, 1H), 7.85 (d, J = 9.7 Hz, 1H), 7.68 (d, 2.1

Hz, 1H), 7.64 (dd, *J* = 2.1, 8.7, 1H), 7.24 (d, *J* = 8.8 Hz, 1H), 7.04 (d, *J* = 9.7 Hz, 1H), 5.28 (s, 2H), 3.90 (s, 3H).

1-[(4-Chloro-3-fluoro-phenyl)methyl]-6-oxo-pyridazine-3-carboxylic acid (29h)

29h was synthesised in two steps from methyl 6-oxo-1,6-dihydropyridazine-3-carboxylate by analogous methods to **28a** and **29a** to yield a white solid (111 mg, 87%). ^{1}H NMR (400 MHz, DMSO- d_6) δ 13.61 (s, 1H), 7.87 (d, *J* = 9.7 Hz, 1H), 7.57 (t, *J* = 8.1 Hz, 1H), 7.36 (dd, *J* = 1.9, 10.3 Hz, 1H), 7.16 (dd, *J* = 1.4, 8.2 Hz, 1H), 7.06 (d, *J* = 9.7 Hz, 1H), 5.33 (s, 2H)

1-((5-Fluoro-6-methoxypyridin-3-yl)methyl)-6-oxo-1,6-dihydropyridazine-3-carboxylic acid (29i)

29i was synthesised in two steps from methyl 6-oxo-1,6-dihydropyridazine-3-carboxylate by analogous methods to **28a** and **29a** to yield a yellow gum (650 mg, 11%). m/z 280.1 $[M+H]^+$

***Tert*-butyl *N*-[3-(cyclopropylcarbamoyl)phenyl]carbamate (30)**

30 was synthesised from *N*-Boc-3-amino benzoic acid and cyclopropylamine by an analogous method to **5** (1.09 g, 62%). ^{1}H NMR (500 MHz, DMSO- d_6) δ 7.79 (s, 1H), 7.51 (d, *J* = 8.5 Hz, 1H), 7.43 (d, *J* = 7.9 Hz, 1H), 7.36 (t, *J* = 7.9 Hz, 1H), 6.56 (bs, 1H), 6.24 (bs, 1H), 2.91 (m, 1H), 1.55 (s, 9H), 0.89 (m, 2H), 0.65 (m, 2H). m/z 277.2 $[M+H]^+$

3-Amino-*N*-cyclopropyl-benzamide (31)

To a solution of **30** (1.09 g, 3.93 mmol) in DCM (40 mL) was added 4M HCl in dioxane (4.9 mL, 19.65 mmol). The reaction was left to stir at RT for 72h, then concentrated under reduced pressure, dissolved in MeOH and the free base liberated using an SCX cartridge, eluting the product with 7N NH_3 in MeOH. Evaporation of the solvent yielded **31** (710 mg, quant. yield)

as a white solid. ^{1}H NMR (500 MHz, DMSO- d_6) δ 8.42 (s, 1H), 7.47 (m, 2H), 7.35 (t, *J* = 7.7 Hz, 1H), 7.16 (d, *J* = 7.1 Hz, 1H), 2.84 (m, 1H), 0.70 (m, 2H), 0.57 (m, 2H).

N-Cyclopropyl-4-fluoro-3-nitrobenzamide (32)

32 was prepared from 2-fluoro-5-nitroaniline and cyclopropylamine by an analogous method to **5** to yield an off-white solid (3.80 g, 63%). ^{1}H NMR (500 MHz, DMSO- d_6) δ 8.65 (bs, 1H), 8.38 (m, 2H), 7.59 (m, 1H), 2.86 (m, 1H), 0.73 (m, 2H), 0.59 (m, 2H). m/z 224 $[M+H]^+$

5-Amino-N-cyclopropyl-2-fluoro-benzamide (33)

To a suspension of **32** (4.20 g, 18.73 mmol) in EtOH (150 mL) was added NH_4Cl (4.0 g, 74.94 mmol) in Water (50 mL) followed by iron powder (8.37 g, 149.87 mmol) and left to stir at rt for 3 h. The mixture was diluted with EtOAc and water and filtered through Celite®. The aqueous layer was extracted with EtOAc (2×100 mL), and the combined organics washed with brine, separated, and concentrated under reduced pressure to give **33** (2.81 g, 77%) as an orange solid. ^{1}H NMR (500 MHz, $CDCl_3$) δ 7.40 (m, 1H), 6.90 (dd, *J* = 8.8, 11.5 Hz, 1H), 6.83 (bd, *J* = 10.4 Hz, 1H), 6.74 (m, 1H), 3.69 (s, 2H), 2.95 (m, 1H), 0.89 (m, 2H), 0.63 (m, 2H). m/z 195 $[M+H]^+$

5-Amino-2-fluoro-*N-(cis*-3-methoxycyclobutyl)benzamide (34)

To a suspension of 5-amino-2-fluorobenzoic acid (150 mg, 0.97 mmol), 1-hydroxybenzotriazole hydrate (192 mg, 1.26 mmol), N-(3-Dimethylaminopropyl)-N'-ethylcarbodiimide hydrochloride (241 mg, 1.26 mmol) and *trans*-3-methoxycyclobutan-1-amine hydrochloride (160 mg, 1.16 mmol) in DCM (10 mL) was added *N,N*-diisopropylethylamine (0.507 mL, 2.90 mmol) and the resulting mixture stirred at RT for 16 h. The reaction mixture was partitioned between water (10 mL) and DCM (50 mL), and the organic layer dried over Na_2SO_4, concentrated, and purified by flash column chromatography

(0 – 100% EtOAc/Cyclohexane) to yield **34** as a white solid (170 mg, 74%). ^{1}H NMR (400 MHz, DMSO- d_6) δ ppm 2.15 - 2.28 (m, 4 H) 3.14 (s, 3 H) 3.92 - 4.04 (m, 1 H) 4.36 (br d, J=6.82 Hz, 1 H) 5.10 (s, 2 H) 5.76 (s, 1 H) 6.61 (ddd, J=8.78, 4.11, 3.03 Hz, 1 H) 6.71 (dd, J=6.06, 3.03 Hz, 1 H) 6.90 (dd, J=10.23, 8.72 Hz, 1 H) 8.41 (br d, J=6.57 Hz, 1 H). m/z 239.0 $[M+H]^+$

5-Amino-2-fluoro-*N*-methylbenzamide (35)

35 was synthesised from 5-amino-2-fluorobenzoic acid and methylamine by an analogous method to **5** (359 mg, 27%). ^{1}H NMR (500 MHz, DMSO- d_6) δ 7.95 (bs, 1H), 6.91 (dd, J = 8.8, 10.5 Hz, 1H), 6.81 (dd, J = 2.9, 6.1 Hz, 1H), 6.63 (m, 1H), 5.08 (bs, 2H), 2.75 (d, J = 4.6 Hz, 3H).

Methyl 3-[(1-benzyl-6-oxo-pyridazine-3-carbonyl)amino]benzoate (36a)

36a was prepared from **29a** and methyl 3-aminobenzoate by an analogous method to **5** to yield an off-white sticky gum (2.93 g, 93%). ^{1}H NMR (500 MHz, DMSO- d_6) δ 8.85 (s, 1H), 8.13 (s, 1H), 8.07 (d, J = 9.7 Hz, 1H), 8.04 (dd, J = 8.1, 1.4 Hz, 1H), 7.87 (d, J = 7.8 Hz, 1H), 7.51 – 7.39 (m, 6H), 7.09 (d, J = 9.7 Hz, 1H), 5.44 (s, 2H), 3.98 (s, 3H). m/z 364.3 $[M+H]^+$

Methyl 5-(1-benzyl-6-oxo-1,6-dihydropyridazine-3-carboxamido)-2-fluorobenzoate (36b)

36b was synthesised from **29a** and methyl 5-amino-2-fluorobenzoate by an analogous method to **5** (150 mg, 91%). ^{1}H NMR (400 MHz, DMSO- d_6) δ 10.57 (bs, 1H), 8.37 (m, 1H), 8.11 (m, 1H), 7.97 (d, J = 9.7 Hz, 1H), 7.43-7.29 (m, 6H), 7.11 (d, J = 9.7 Hz, 1H), 5.38 (s, 2H), 3.88 (s, 3H).

Methyl 3-(1-benzyl-6-oxo-1,6-dihydropyridazine-3-carboxamido)-4-fluorobenzoate (36c)

36c was synthesised from **29a** and methyl 3-amino-4-fluorobenzoate by an analogous method to **5** (200 mg, quant. yield). ^{1}H NMR (500 MHz, DMSO- d_6) δ 10.15 (s, 1H), 8.42 (dd, J = 2.0, 7.4 Hz, 1H), 7.97 (d, J = 9.7 Hz, 1H), 7.89 (m, 1H), 7.50 (dd, J = 8.8, 10.2 Hz, 1H), 7.44 (m, 2H), 7.38 (t, J = 7.3 Hz, 2H), 7.33 (m, 1H), 7.13 (d, J = 9.7 Hz, 1H), 5.37 (s, 2H), 3.88 (s, 3H).

Methyl-2-fluoro-5-[[1-[(3-fluoro-4-methoxy-phenyl)methyl]-6-oxo-pyridazine-3-carbonyl]amino]benzoate (36d)

36d Was synthesised from **29f** and methyl 5-amino-2-fluorobenzoate by an analogous method to **5** to yield an off-white solid (4.46 g, 96%). ^{1}H NMR (400 MHz, DMSO- d_6) δ 10.52 (s, 1H), 8.36 (dd, J = 2.8, 6.5 Hz, 1H), 8.10 (d, J = 3.2 Hz, 1H), 7.96 (d, J = 9.7 Hz, 1H), 7.40 (dd, J = 9.2, 10.5 Hz, 1H), 7.35 (dd, J = 1.9, 12.3 Hz, 1H), 7.24 (d, J = 8.4 Hz, 1H), 7.15 (t, J = 8.7 Hz, 1H) 7.10 (d, J = 9.7 Hz, 1H), 5.30 (s, 2H), 3.89 (s, 3H), 3.82 (s, 3H). m/z 430.3 $[M+H]^+$

Methyl 2-fluoro-5-[[1-[(4-methoxyphenyl)methyl]-6-oxo-pyridazine-3-carbonyl]amino] benzoate (36e)

36e was synthesised from **29c** and methyl 5-amino-2-fluorobenzoate by an analogous method to **5** to yield a white solid (489 mg, quant. yield) afforded as a white solid. ^{1}H NMR (400 MHz, DMSO- d_6) δ 10.56 (s, 1H), 8.37 (dd, J = 2.8, 6.5 Hz, 1H), 8.11 (m, 1H), 7.95 (d, J = 9.7 Hz, 1H), 7.40 (m, 3H), 7.09 (d, J = 9.7 Hz, 1H), 6.92 (m, 2H), 5.31 (s, 2H), 3.89 (s, 3H), 3.73 (s, 3H). m/z 412.0 $[M+H]^+$

Methyl-3-(1-benzyl-*N*-methyl-6-oxo-1,6-dihydropyridazine-3-carboxamido)benzoate (37)

To a solution of **36a** (0.630 g, 1.73 mmol) in DMF (6 mL) stirred under nitrogen at 0°C was added potassium carbonate (0.719 g, 5.20 mmol) followed by iodomethane (0.163 mL, 2.60 mmol). The reaction mixture was stirred at RT for 32 h, further iodomethane (0.108 mL, 1.73

mmol) added at 0°C and stirring continued at RT for 16 h. Water (40 mL) / EtOAc (75 mL) were added, the organic layer separated, and the aqueous layer extracted with EtOAc (2×100 mL). The combined organic layers were washed with brine (100 mL), dried over Na_2SO_4, and concentrated under reduced pressure. Crude material was purified by flash chromatography (40% EtOAc / Pet. Ether) to afford **37** (0.568 g, 87%). m/z 378 $[M+H]^+$

(1-Benzyl-*N*-methyl-6-oxo-1,6-dihydropyridazine-3-carboxamido)benzoic acid (38)

38 was synthesised from **37** by an analogous method to **29a** using LiOH at 0 °C, rather than NaOH, to yield a yellow semisolid (230 mg, 41%) m/z 364 $[M+H]^+$

3-(1-Benzyl-6-oxo-1,6-dihydropyridazine-3-carboxamido)benzoic acid (39a)

39a was synthesised by an analogous method to **29a** to yield an off-white solid (2623 mg, 93%). ^{1}H NMR (500 MHz, DMSO- d_6) δ 13.00 (s, 1H), 10.47 (s, 1H), 8.41 (s, 1H), 8.08 (d, *J* = 7.9 Hz, 1H), 7.98 (d, *J* = 9.7 Hz, 1H), 7.73 (d, *J* = 7.8 Hz, 1H), 7.52 (t, 7.9 Hz, 1H), 7.43 (d, *J* = 7.3 Hz, 2H), 7.37 (t, *J* = 7.4 Hz, 2H), 7.31 (t, *J* = 7.3 Hz, 1H), 7.11 (d, *J* = 9.7 Hz, 1H), 5.40 (s, 2H). m/z 350.1 $[M+H]^+$

5-(1-Benzyl-6-oxo-1,6-dihydropyridazine-3-carboxamido)-2-fluorobenzoic acid (39b)

39b was synthesised from **36b** by an analogous method to **29a** to yield a pink solid (133 mg, 92%). ^{1}H NMR (400 MHz, DMSO- d_6) δ 13.37 (bs, 1H), 10.54 (s, 1H), 8.33 (dd, *J* = 6.6, 2.8 Hz, 1H), 8.07 (m, 1H), 7.97 (d, *J* = 9.7 Hz, 1H), 7.43 - 7.29 (m, 6H), 7.11 (d, *J* = 9.7 Hz, 1H), 5.38 (s, 2H).

3-(1-Benzyl-6-oxo-1,6-dihydropyridazine-3-carboxamido)-4-fluorobenzoic acid (39c)

39c was synthesised from **36c** by an analogous method to **29a** to yield an off-white solid (84 mg, 54%). ^{1}H NMR (500 MHz, DMSO- d_6) δ 10.18 (s, 1H), 8.36 (dd, *J* = 1.8, 7.6 Hz, 1H),

7.96 (d, J = 9.7 Hz, 1H), 7.86 (m, 1H), 7.46 (m, 3H), 7.35 (m, 4H), 7.12 (d, J = 9.7 Hz, 1H), 5.37 (s, 2H).

2-Fluoro-5-[[1-[(3-fluoro-4-methoxy-phenyl)methyl]-6-oxo-pyridazine-3-carbonyl]amino]benzoic acid (39d)

39d Was synthesised from **36d** by an analogous method to **29a** to yield an off-white solid (4.30 g, 99%). ^{1}H NMR (400 MHz, DMSO- d_6) δ 13.31 (bs, 1H), 10.48 (s, 1H), 8.32 (dd, J = 2.8, 6.6 Hz, 1H), 8.07 (m, 1H), 7.96 (d, J = 9.7 Hz, 1H), 7.35 (m, 1H), 7.25 (d, J = 7.8 Hz, 1H), 7.15 (t, 8.7 Hz, 1H), 7.09 (d, J = 9.7 Hz, 1H), 5.30 (s, 2H), 3.82 (s, 3H). m/z 416.2 $[M+H]^+$

^{1}H, ^{13}C NMR and HPLC traces of final compounds

Compound 5

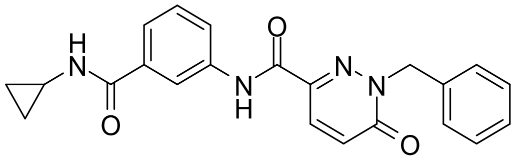

^{1}H NMR

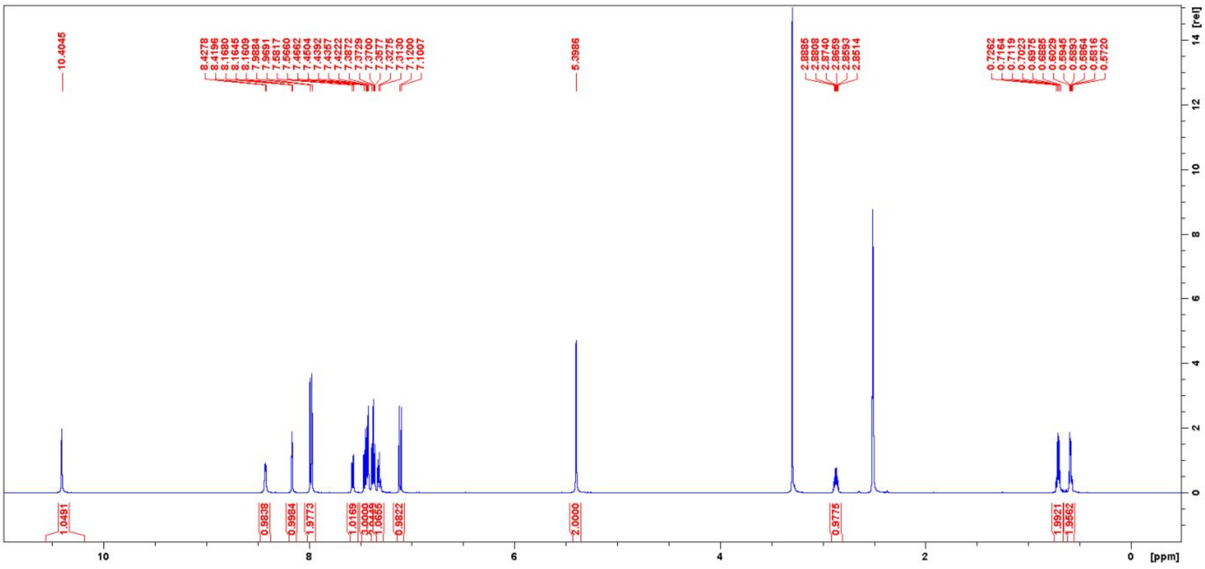

^{13}C NMR

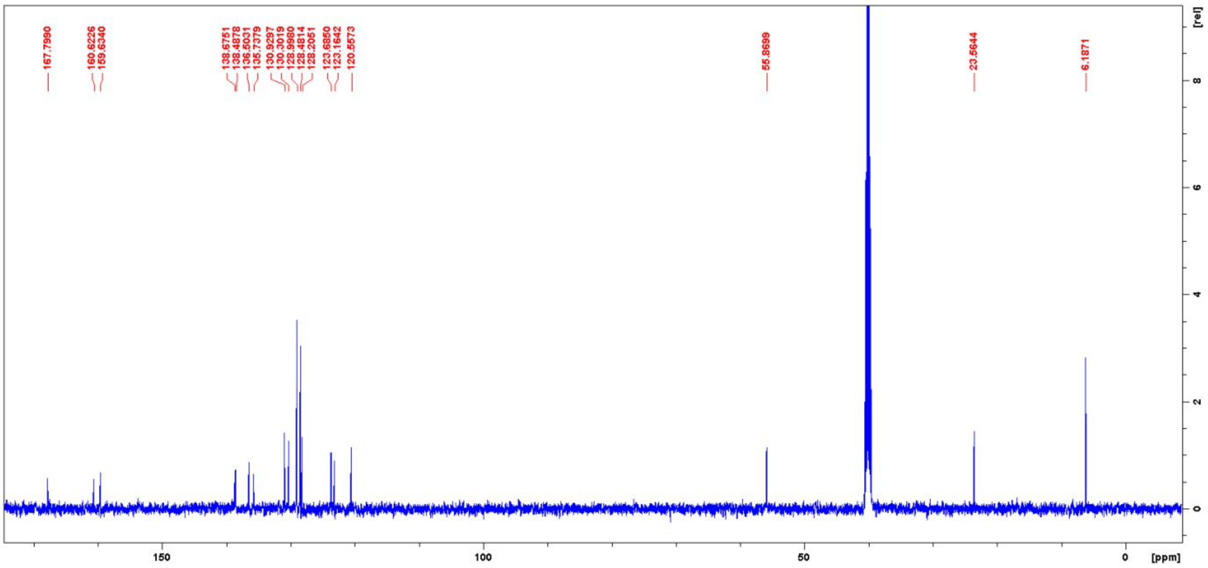

HPLC

UV 254 nm

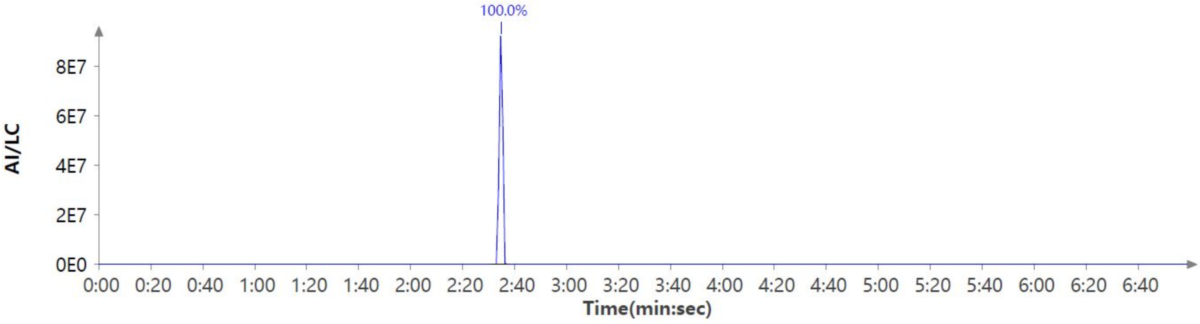

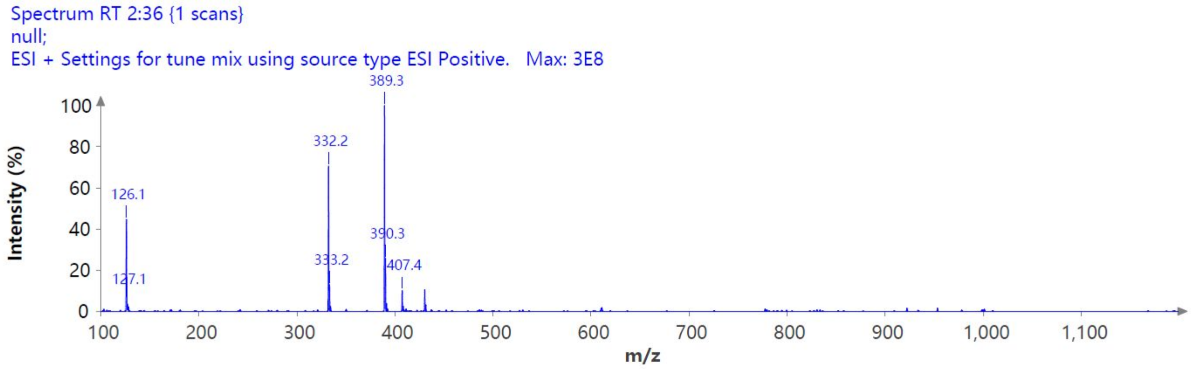

Compound 6

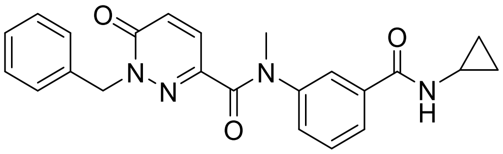

^{1}H NMR

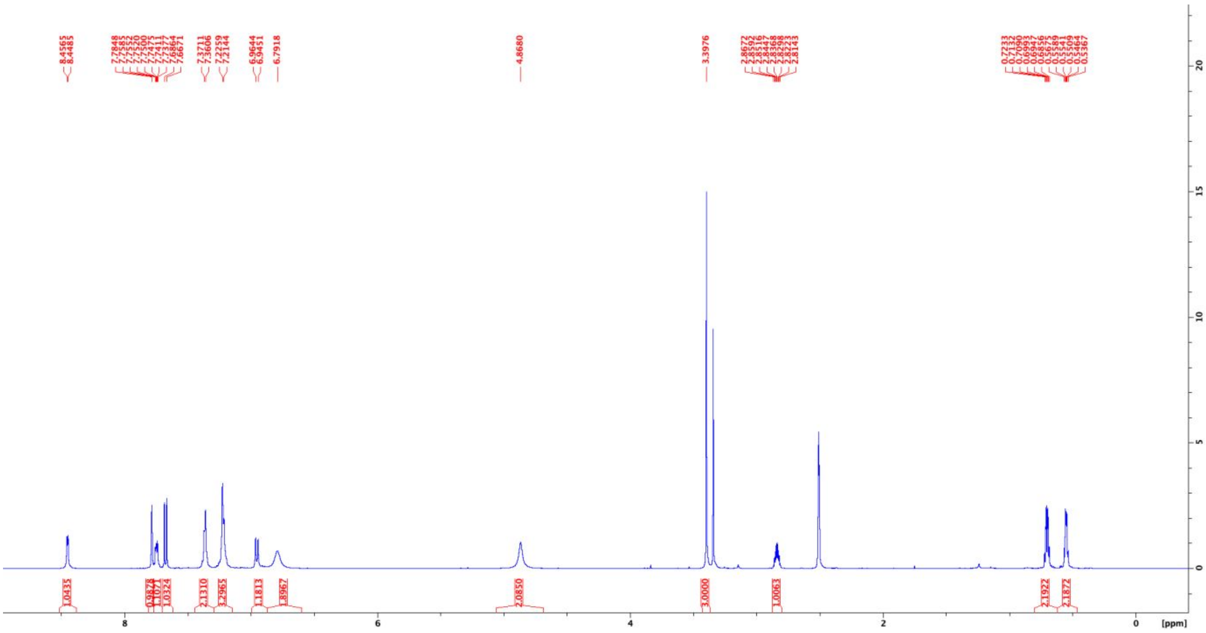

^{13}C NMR

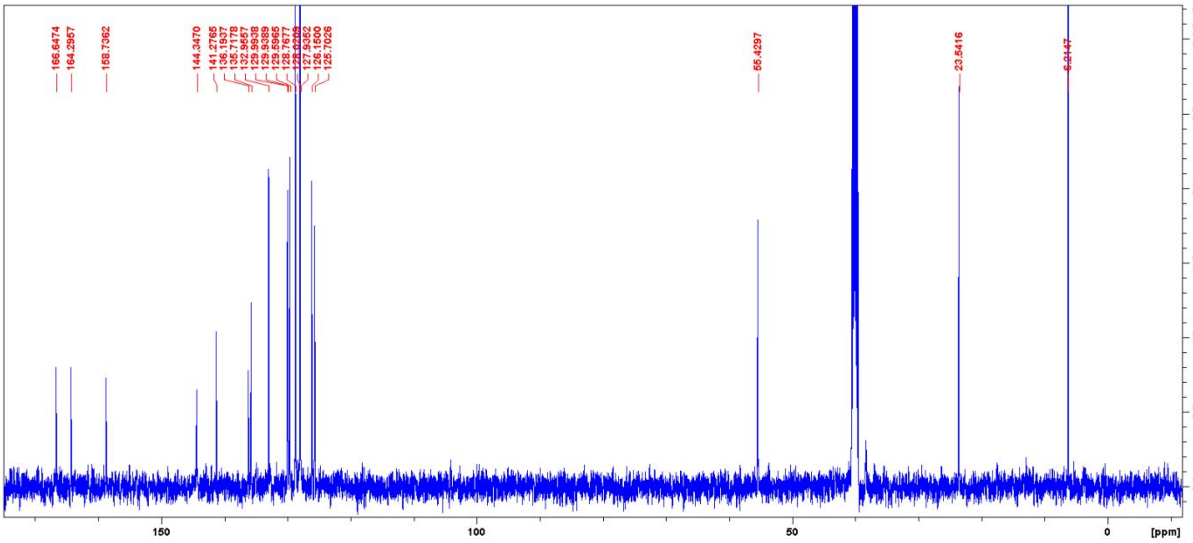

HPLC

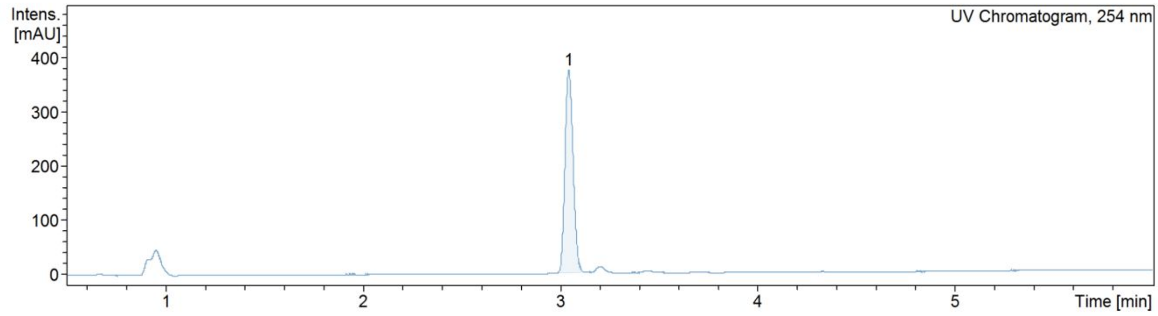

#	RT [min]	Area Frac. %	Chromatogram
1	3.0	100.00	UV Chromatogram, 254 nm
2	3.1	95.03	BPC 74.0000-1601.0000 +, Masses excluded
3	3.2	4.97	BPC 74.0000-1601.0000 +, Masses excluded

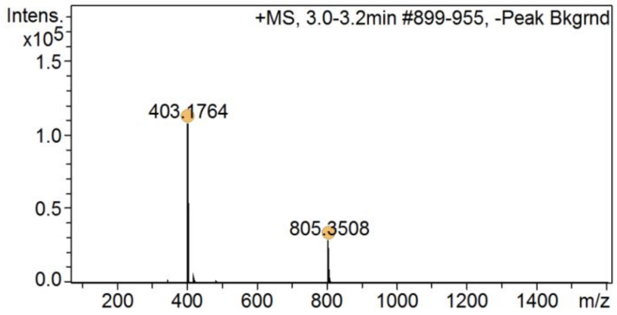

Compound 7

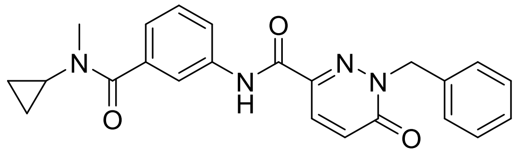

^{1}H NMR

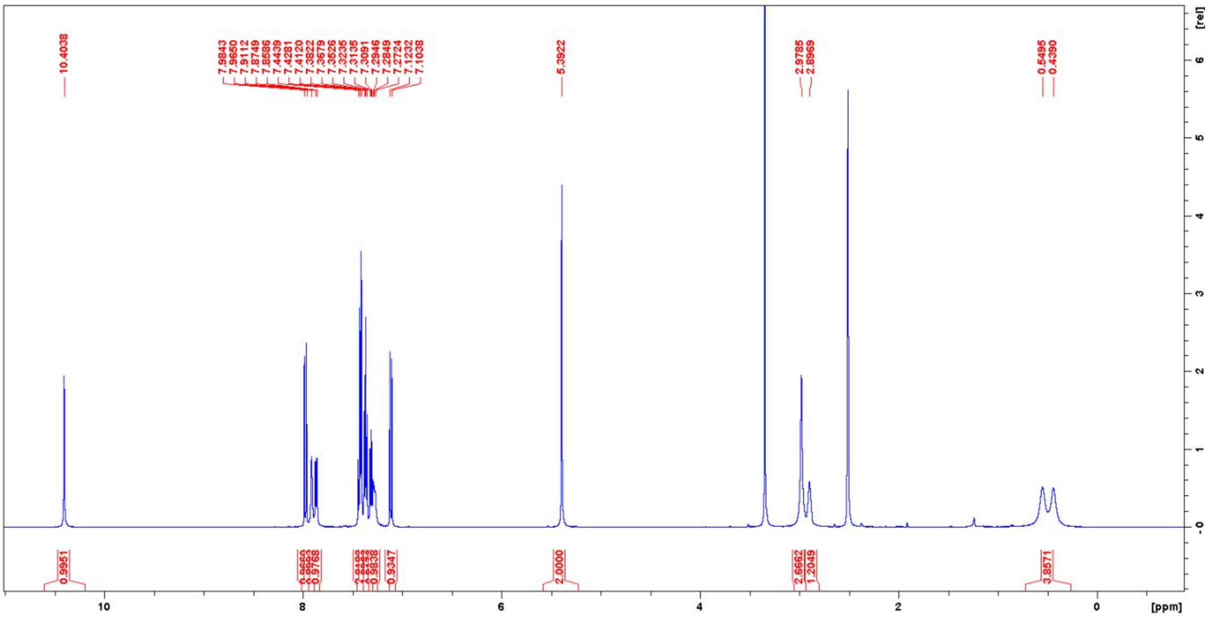

^{13}C NMR

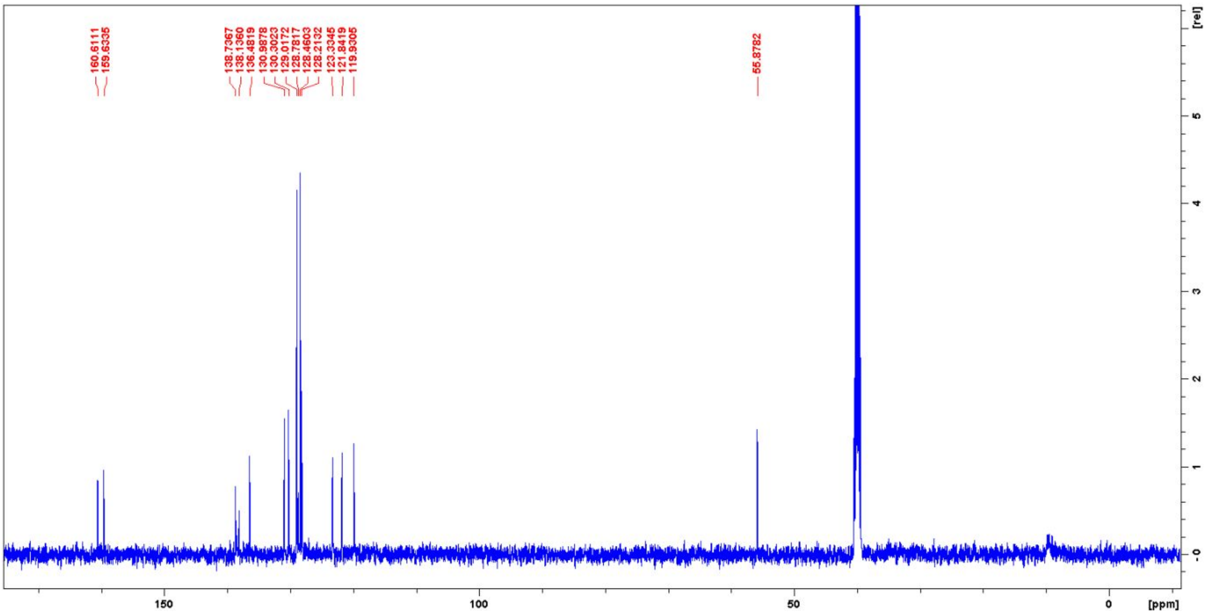

HPLC

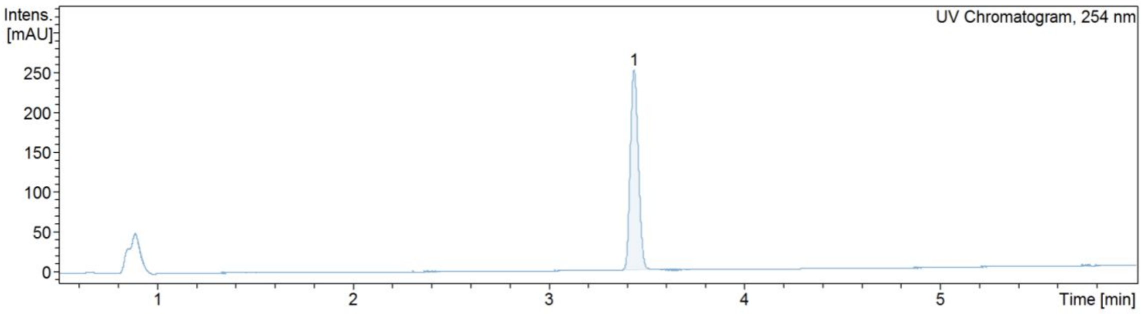

#	RT [min]	Area Frac. %	Chromatogram
1	3.4	100.00	UV Chromatogram, 254 nm
2	3.5	100.00	BPC 74.0000-1600.0000 +, Masses excluded

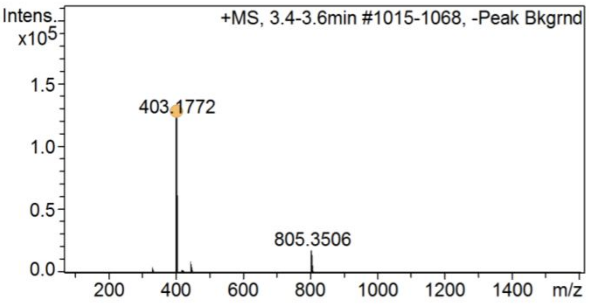

Compound 8

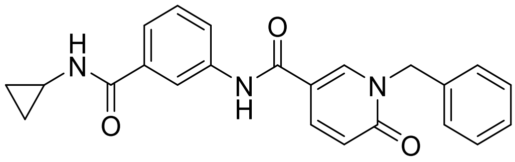

^{1}H NMR

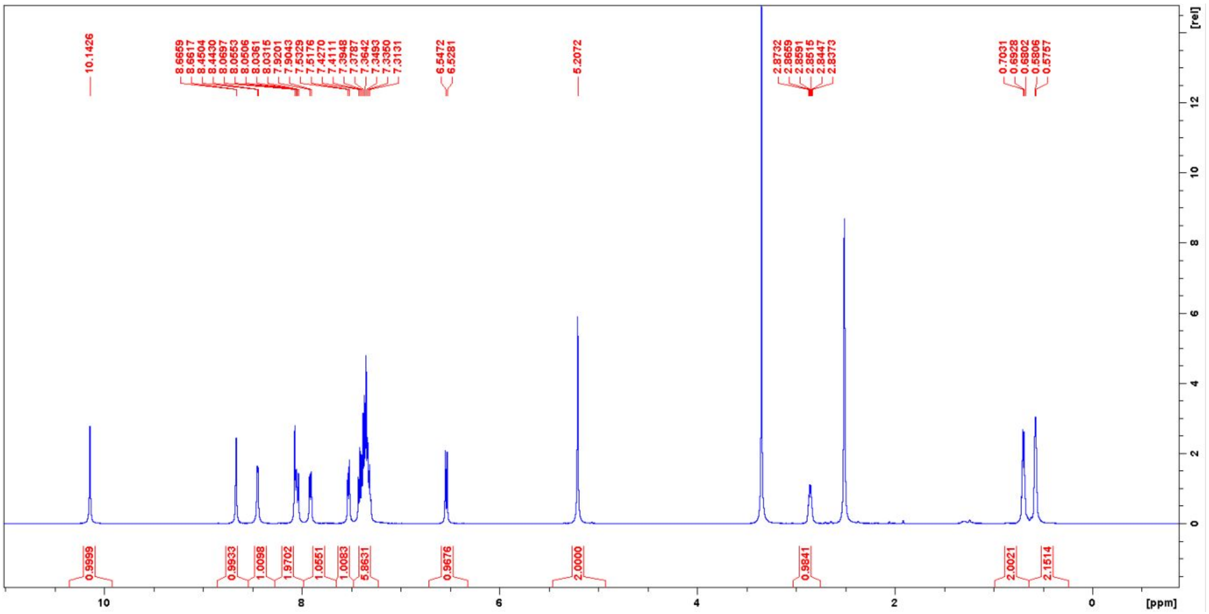

^{13}C NMR

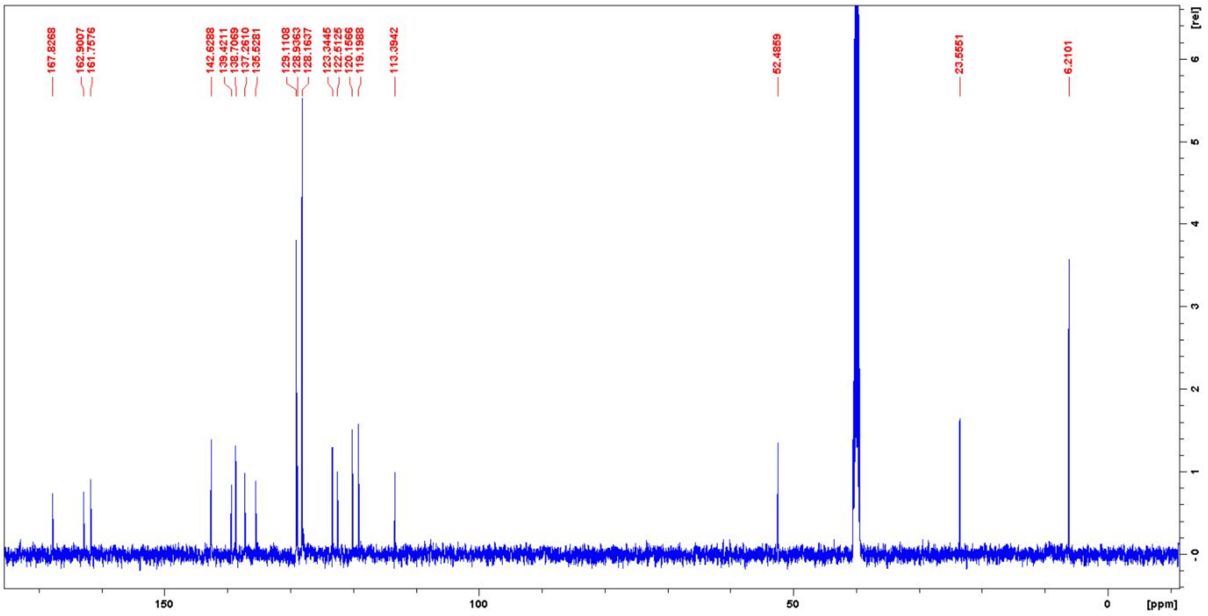

HPLC

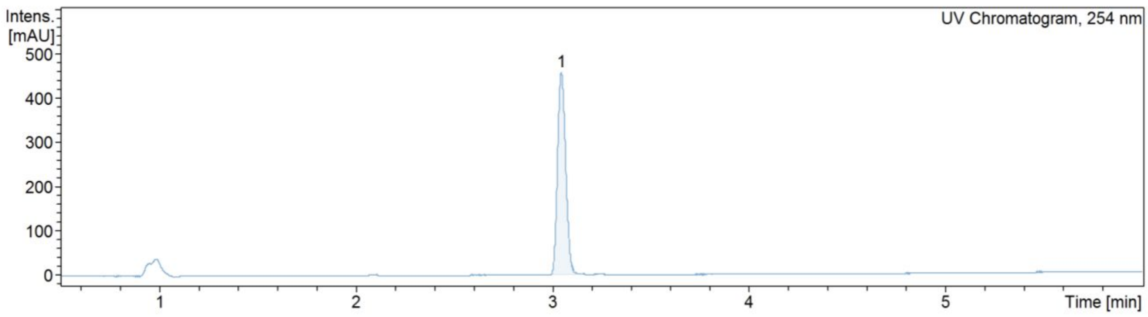

#	RT [min]	Area Frac. %	Chromatogram
1	3.0	100.00	UV Chromatogram, 254 nm
2	3.1	100.00	BPC 74.0000-1601.0000 +, Masses excluded

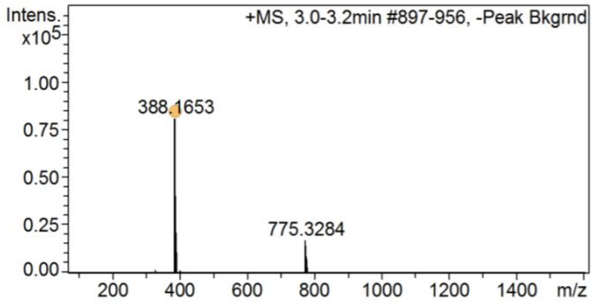

Compound 9

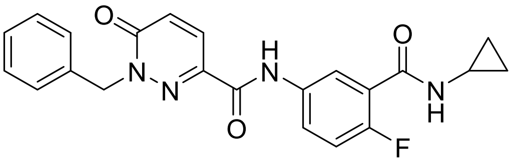

^{1}H NMR

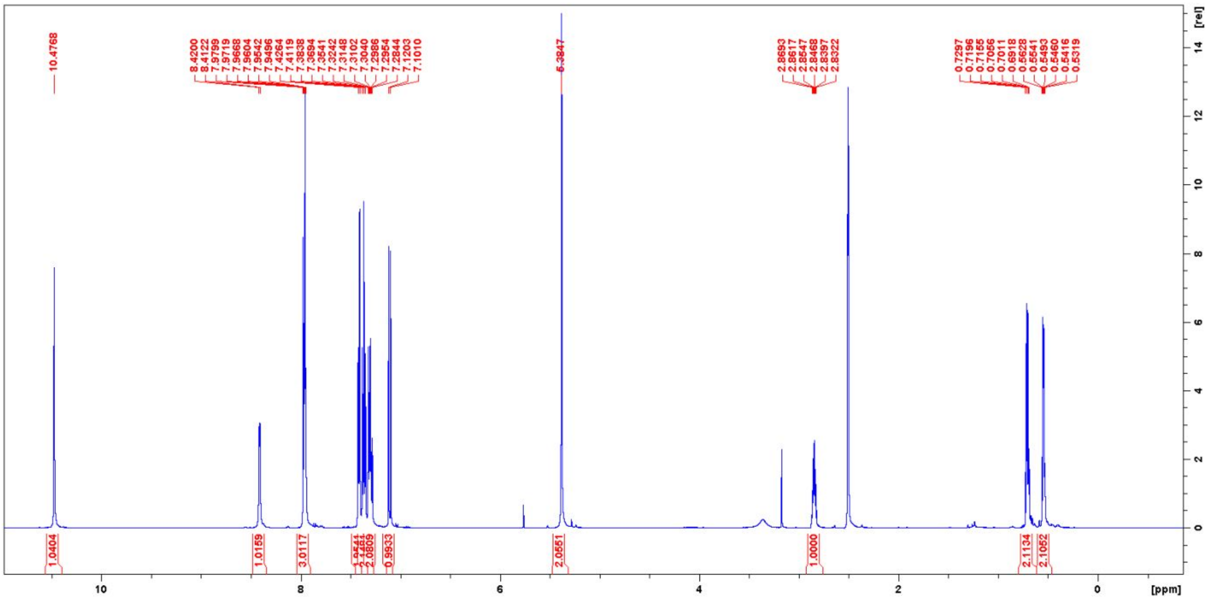
10.4768
8.4200
8.4122
7.9799
7.9719
7.9668
7.9604
7.9542
7.9496
7.4264
7.4119
7.3838
7.3694
7.3541
7.3242
7.3148
7.3102
7.3040
7.2986
7.2864
7.2844
7.1203
7.1010
5.3847
2.8693
2.8617
2.8547
2.8468
2.8397
2.8322
0.7297
0.7196
0.7155
0.7056
0.7011
0.6918
0.5628
0.5541
0.5493
0.5460
0.5416
0.5319
1.0404
1.0159
3.0117
2.0809
0.9933
2.0551
1.0000
2.1134
2.1062
[ppm]
[rel]

^{13}C NMR

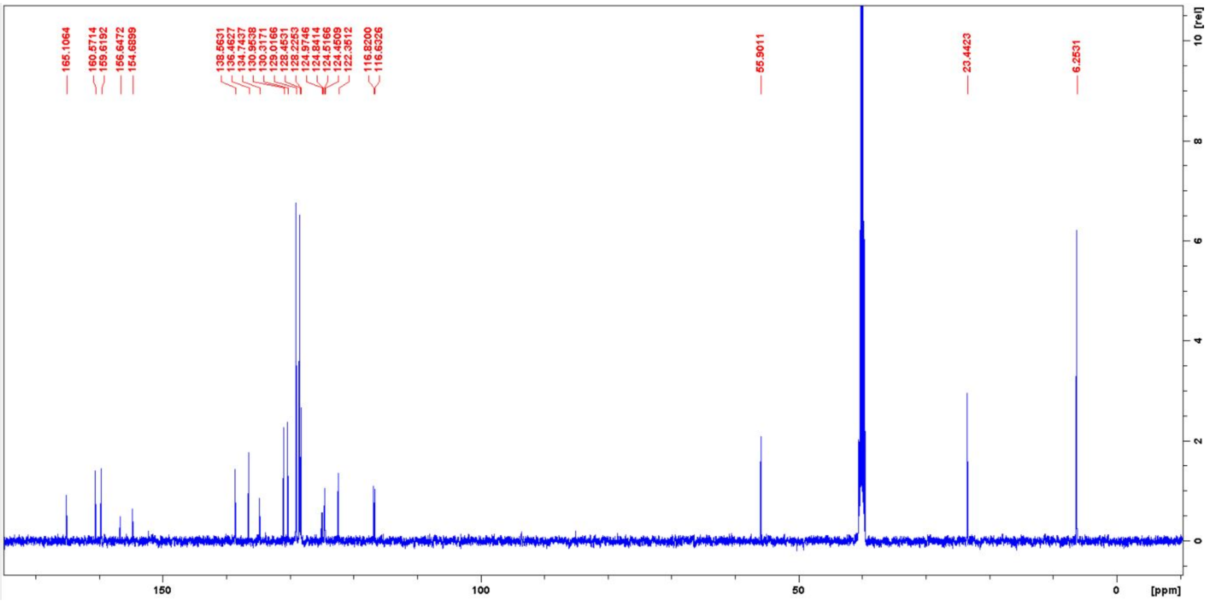
165.1064
160.5714
159.6192
156.6472
154.6899
138.5631
136.4627
134.7437
130.9638
130.3171
129.0166
128.4531
128.2253
124.9746
124.8414
124.5166
124.4509
122.3512
116.8200
116.6326
55.9011
23.4423
6.2531
[ppm]
[rel]

HPLC

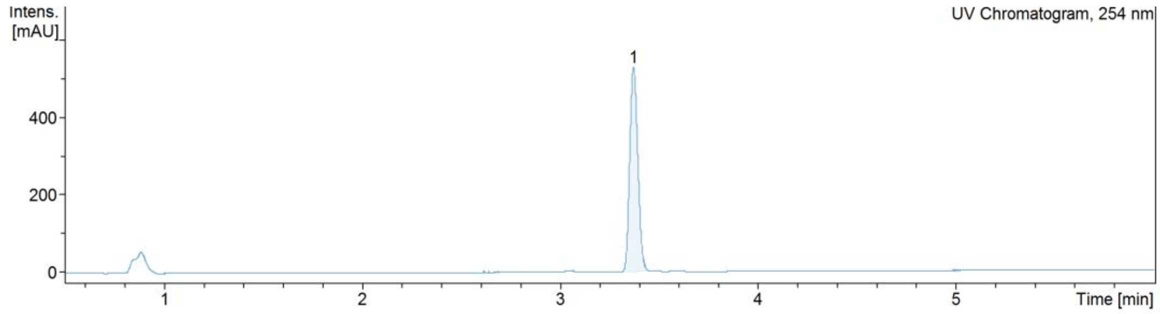

#	RT [min]	Area Frac. %	Chromatogram
1	3.4	100.00	UV Chromatogram, 254 nm
2	3.4	100.00	BPC 74.0000-1601.0000 +, Masses excluded

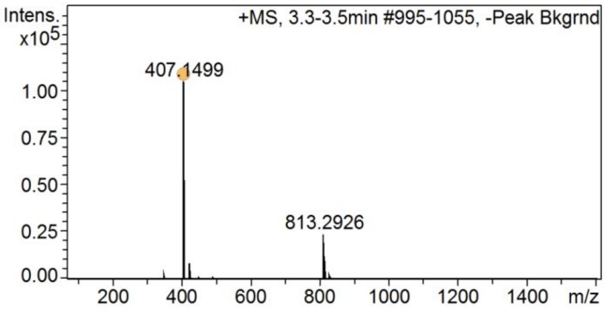

Compound 10

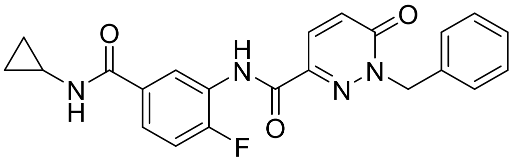

^{1}H NMR

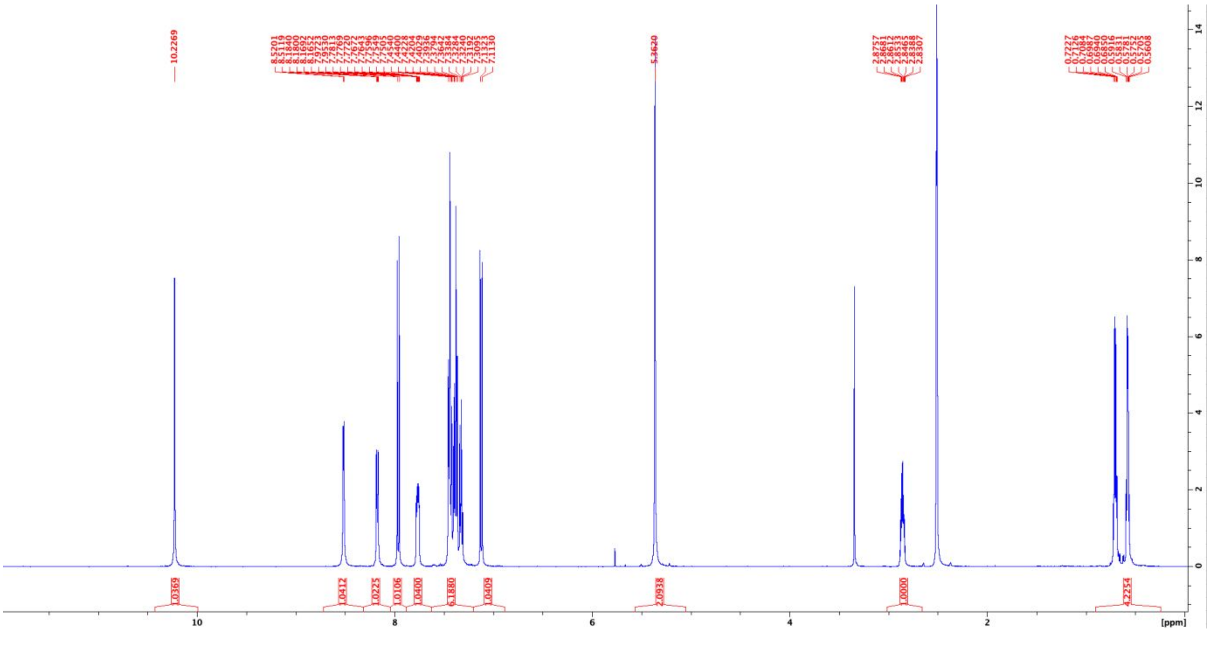

^{13}C NMR

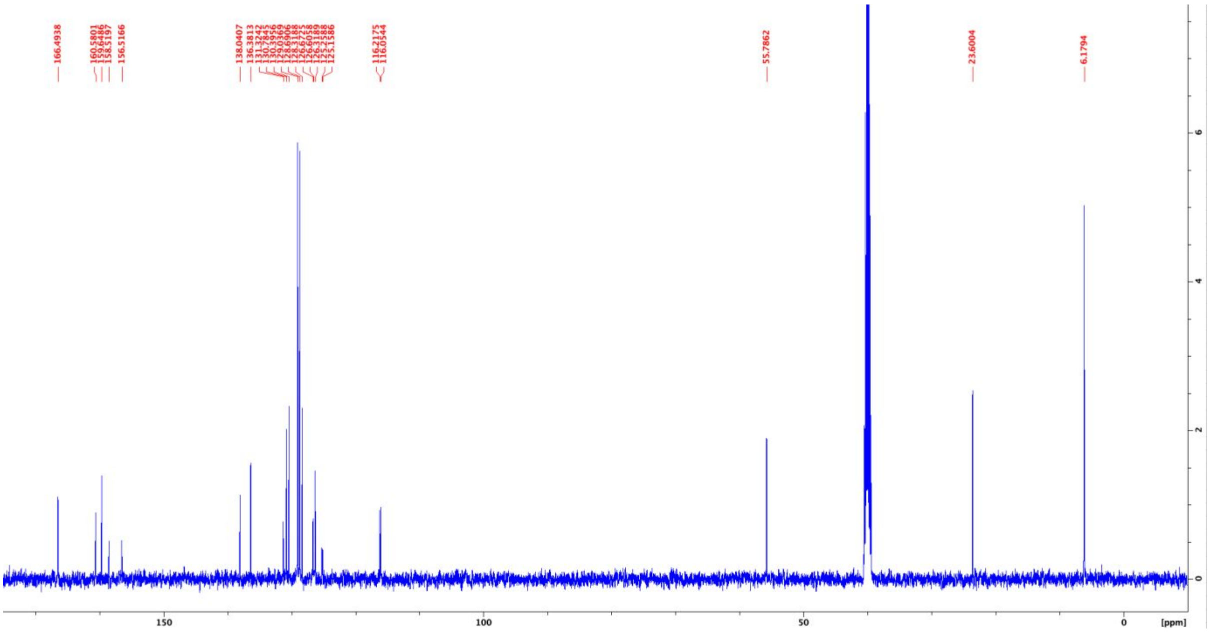

HPLC

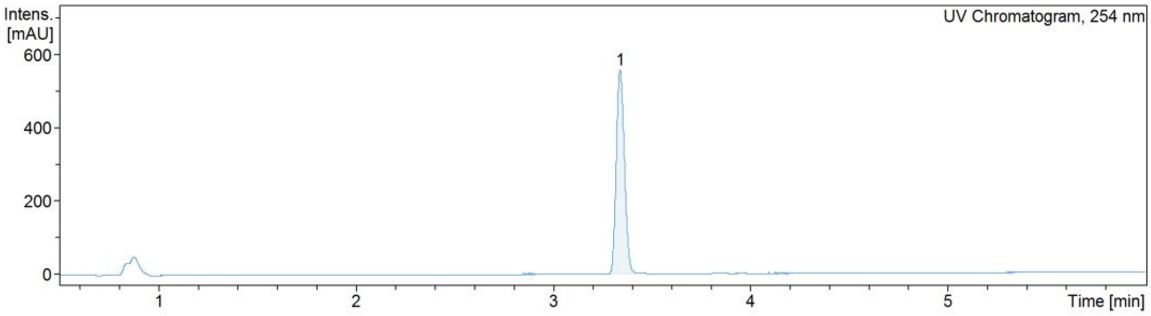

#	RT [min]	Area Frac. %	Chromatogram
1	3.3	100.00	UV Chromatogram, 254 nm
2	3.4	100.00	BPC 74.0000-1601.0000 +, Masses excluded

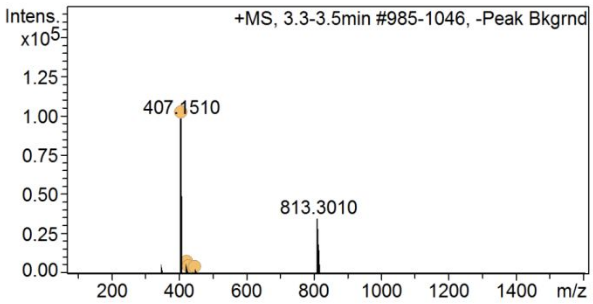

Compound 11

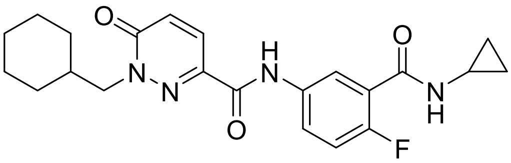

^{1}H NMR

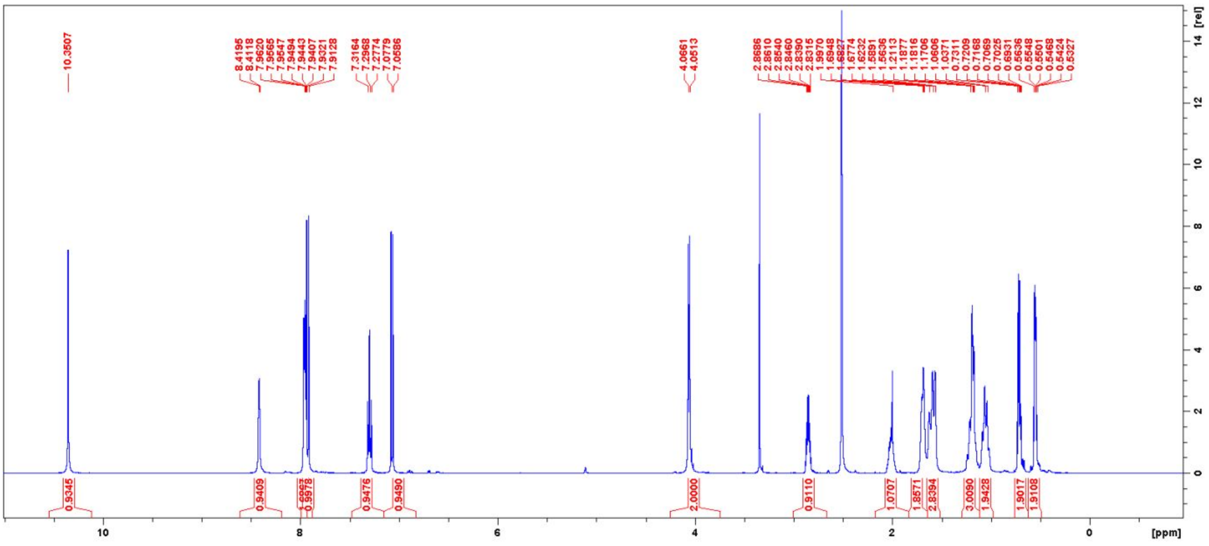

^{13}C NMR

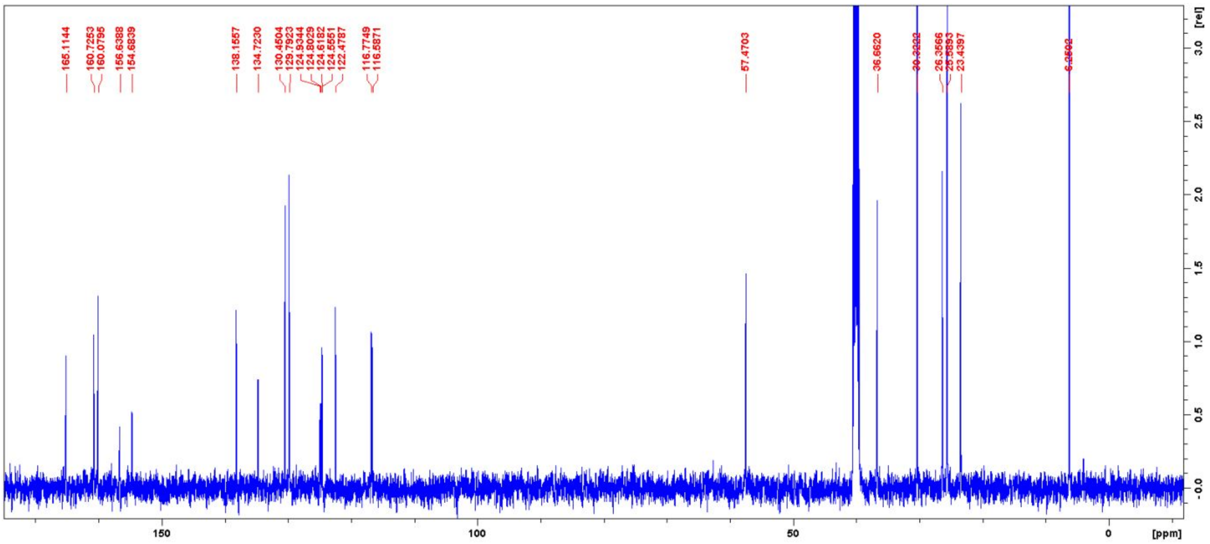

HPLC

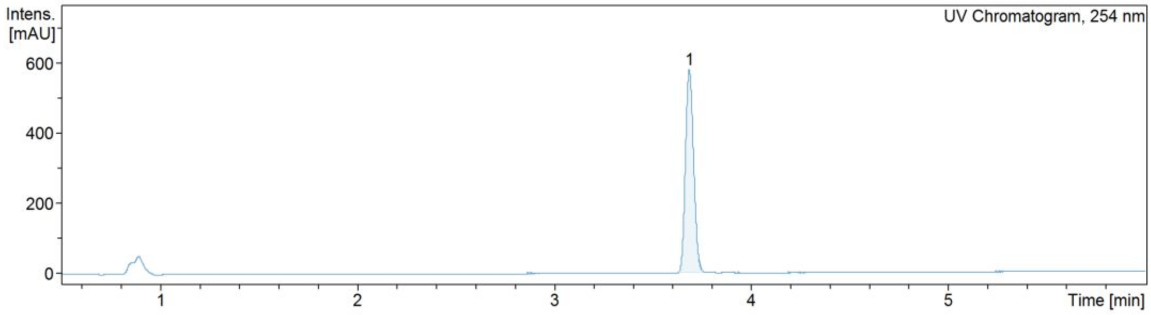

#	RT [min]	Area Frac. %	Chromatogram
1	3.7	100.00	UV Chromatogram, 254 nm
2	3.7	100.00	BPC 75.0000-1601.0000 +, Masses excluded

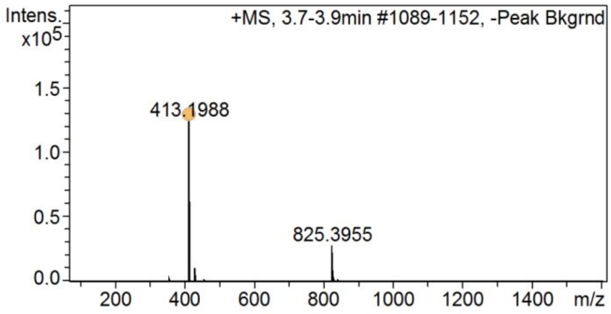

Compound 12

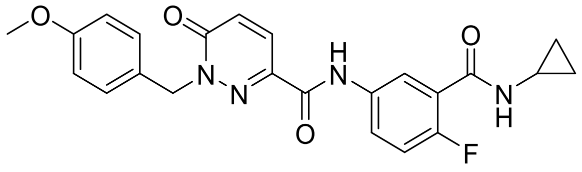

^{1}H NMR

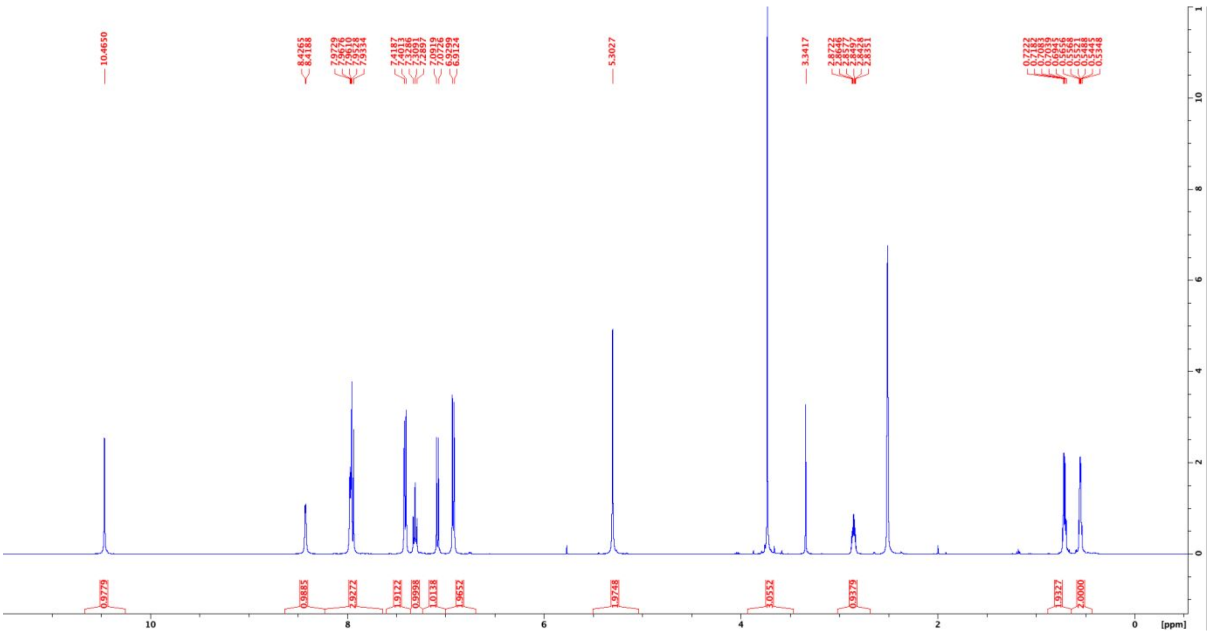

^{13}C NMR

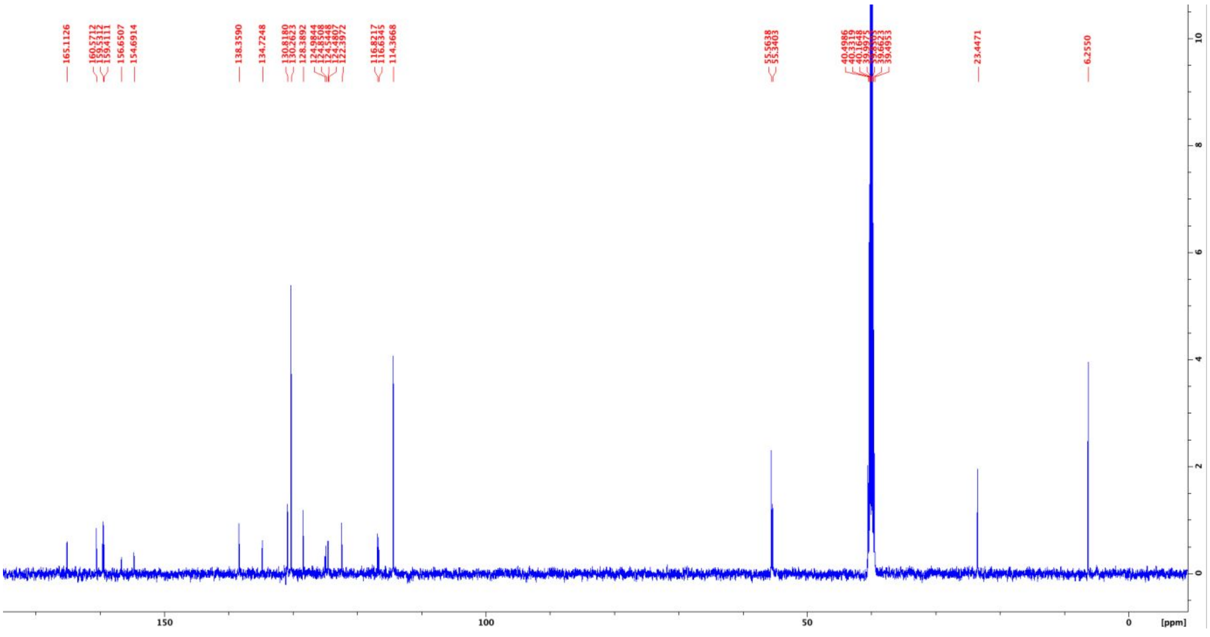

HPLC

MS SQD Waters, LC UPLC Waters, ELSD Waters 2424, Acquity UPLC BEH C18 1.7u 3x50mm

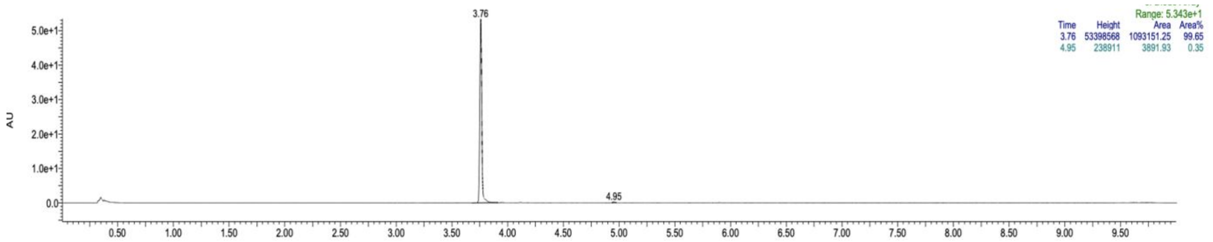

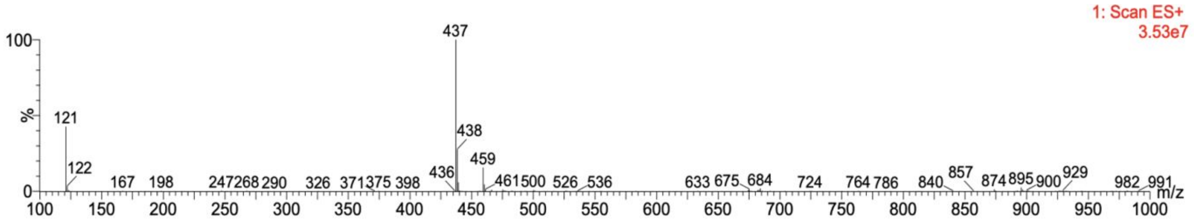

Compound 13

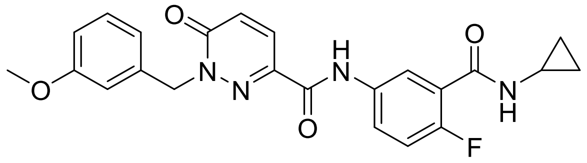

^{1}H NMR

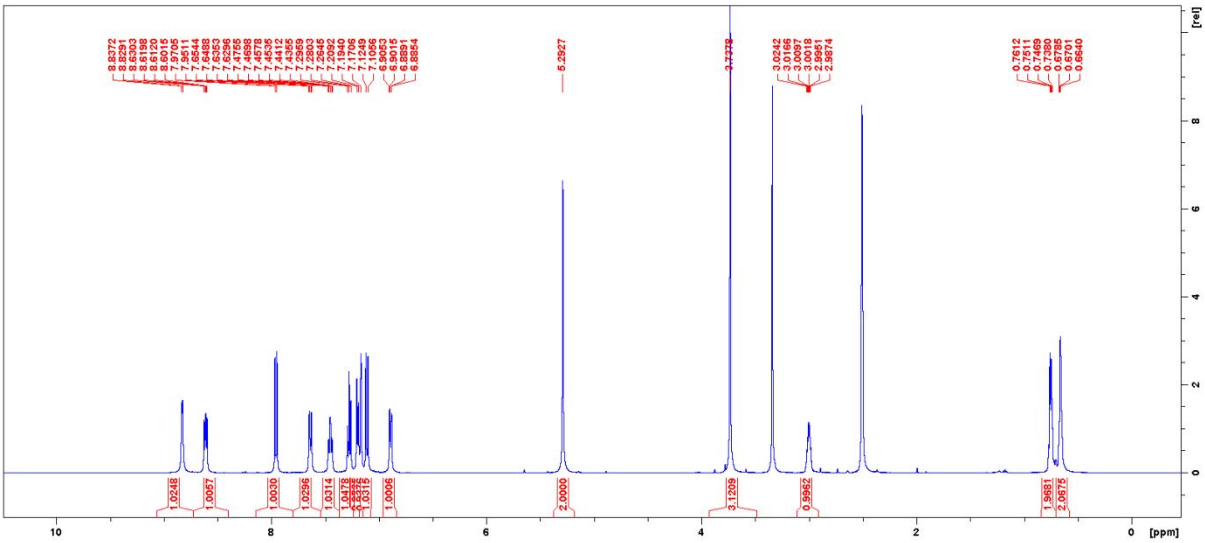

^{13}C NMR

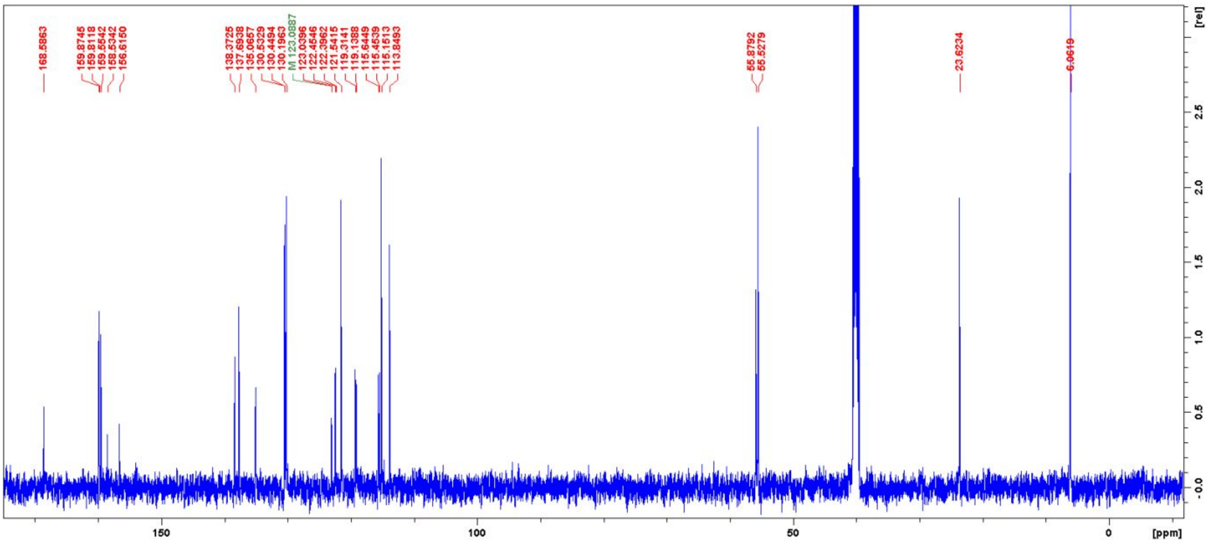

HPLC

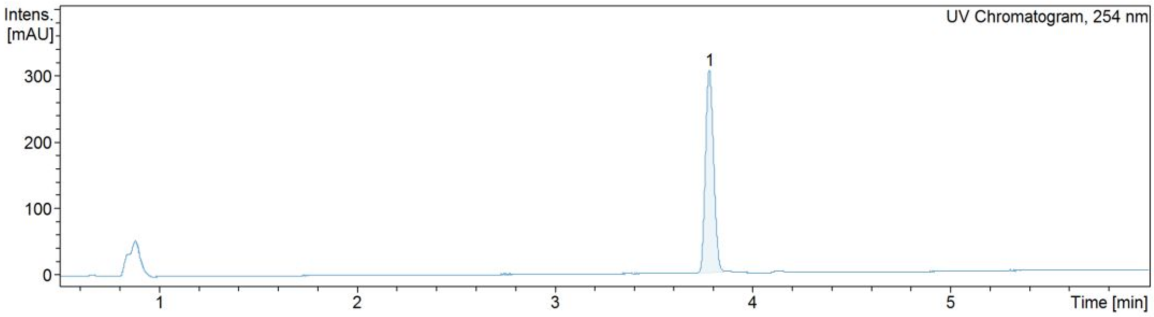

#	RT [min]	Area Frac. %	Chromatogram
1	3.8	100.00	UV Chromatogram, 254 nm
2	3.8	100.00	BPC 75.0000-1601.0000 +, Masses excluded

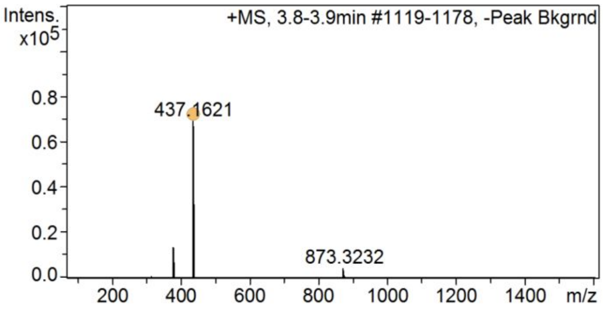

Compound 14

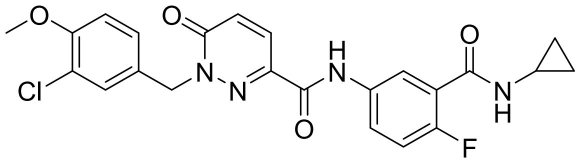

^{1}H NMR

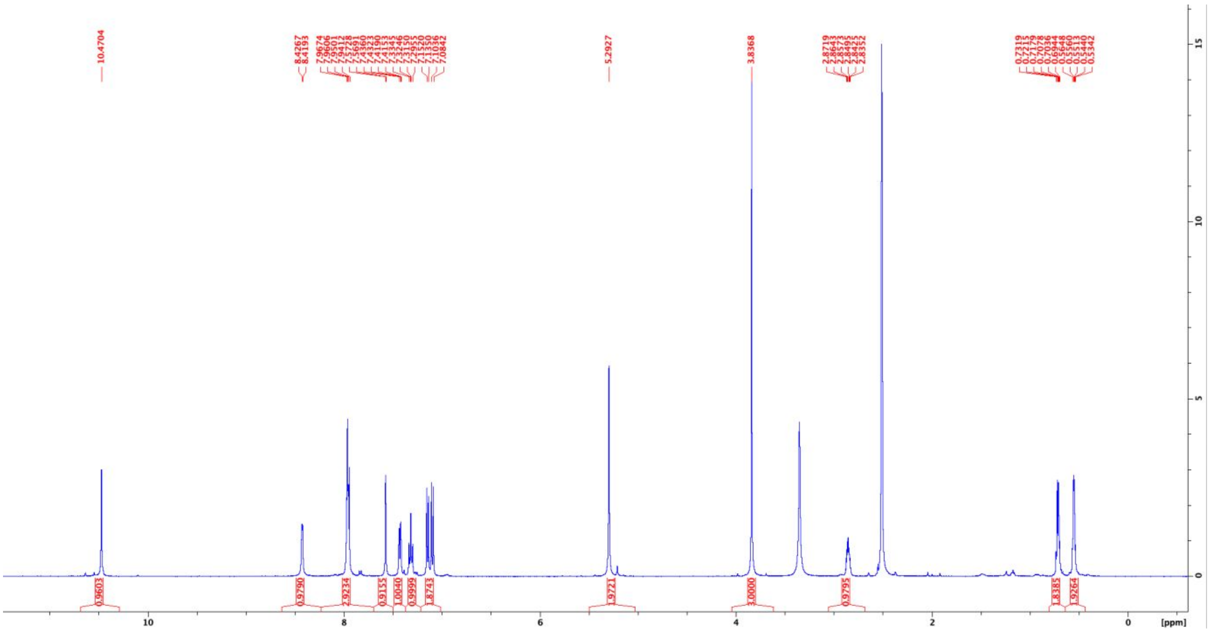

^{13}C NMR

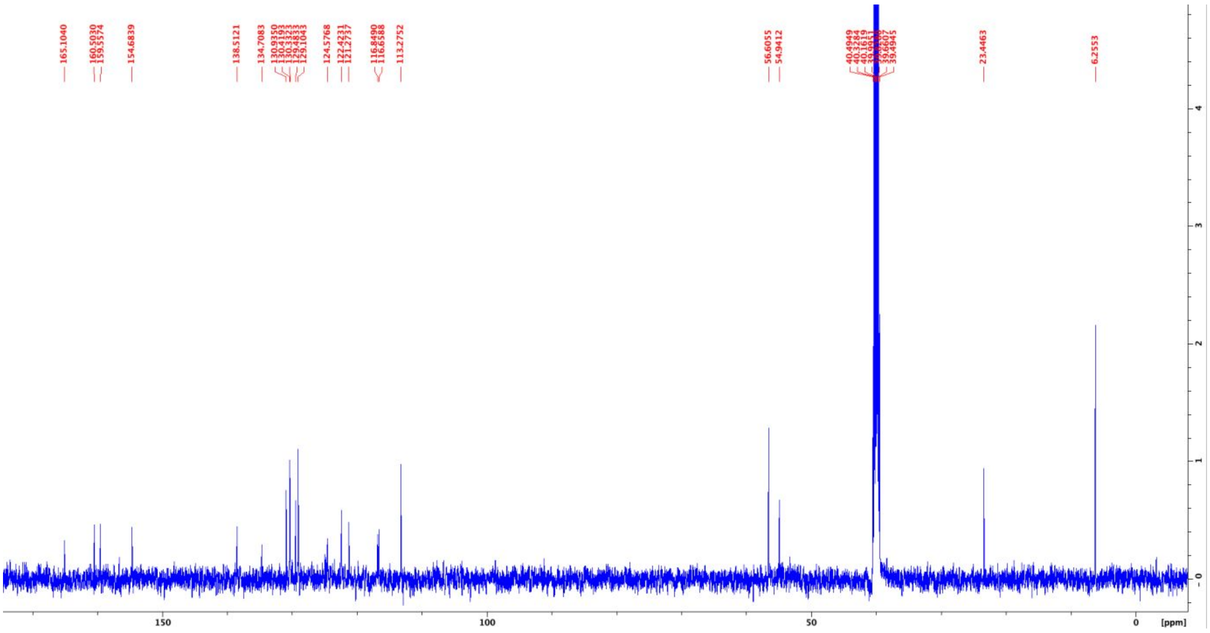

HPLC

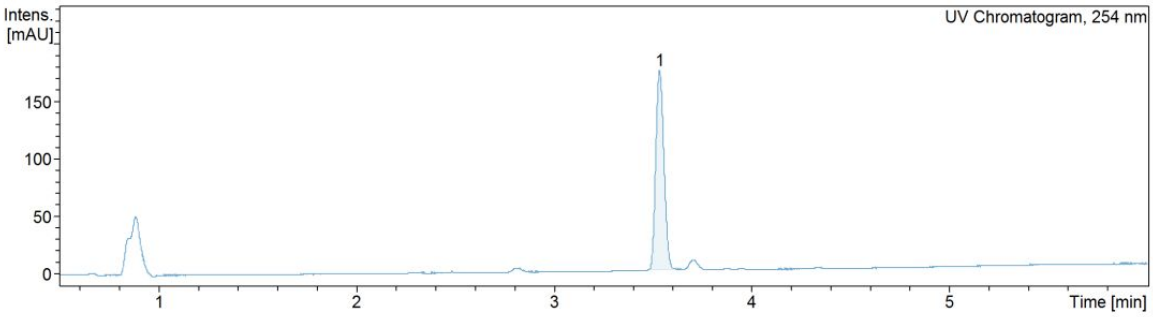

#	RT [min]	Area Frac. %	Chromatogram
1	3.5	100.00	UV Chromatogram, 254 nm
2	3.6	100.00	BPC 74.0000-1600.0000 +, Masses excluded

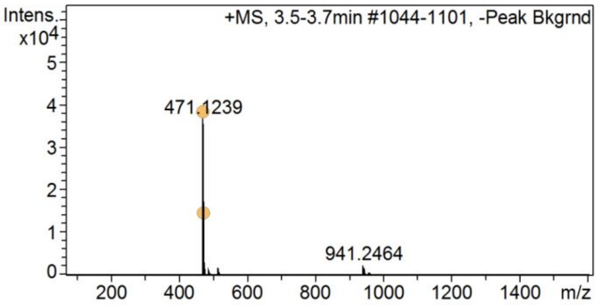

Compound 15

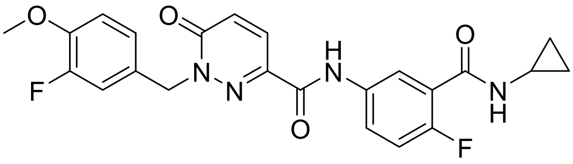

^{1}H NMR

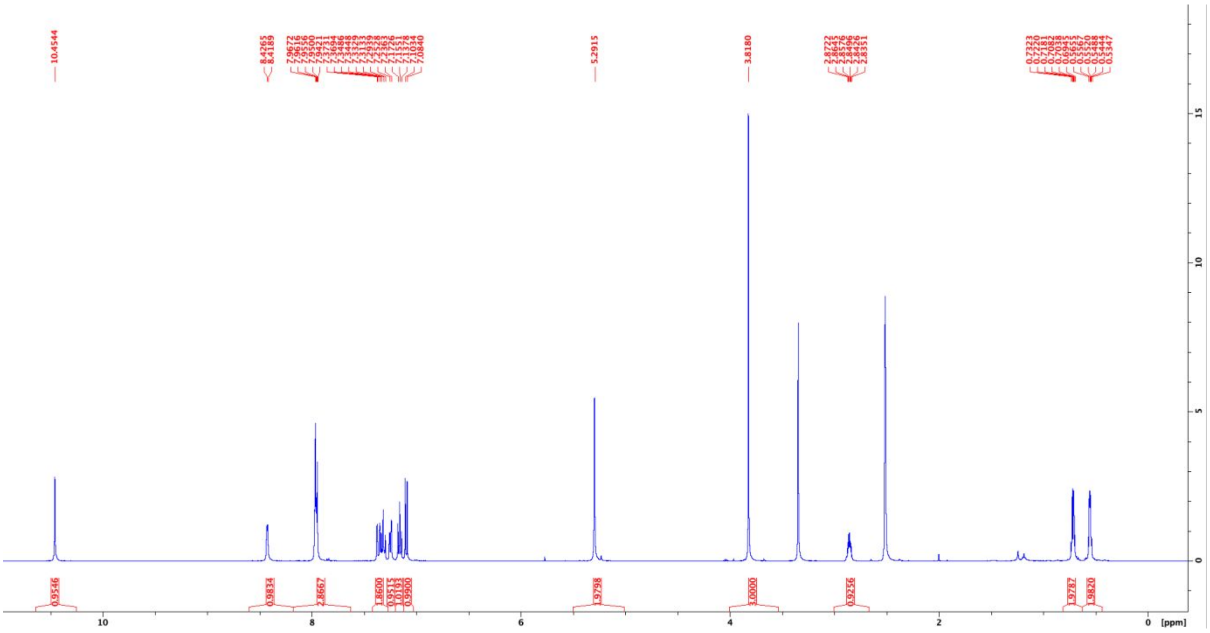
10.4544
8.4265
8.4189
5.2915
3.8180
0.9546
0.9834
2.8667
1.8600
1.9798
3.0000
0.9256
1.9787
1.9820
[ppm]

^{13}C NMR

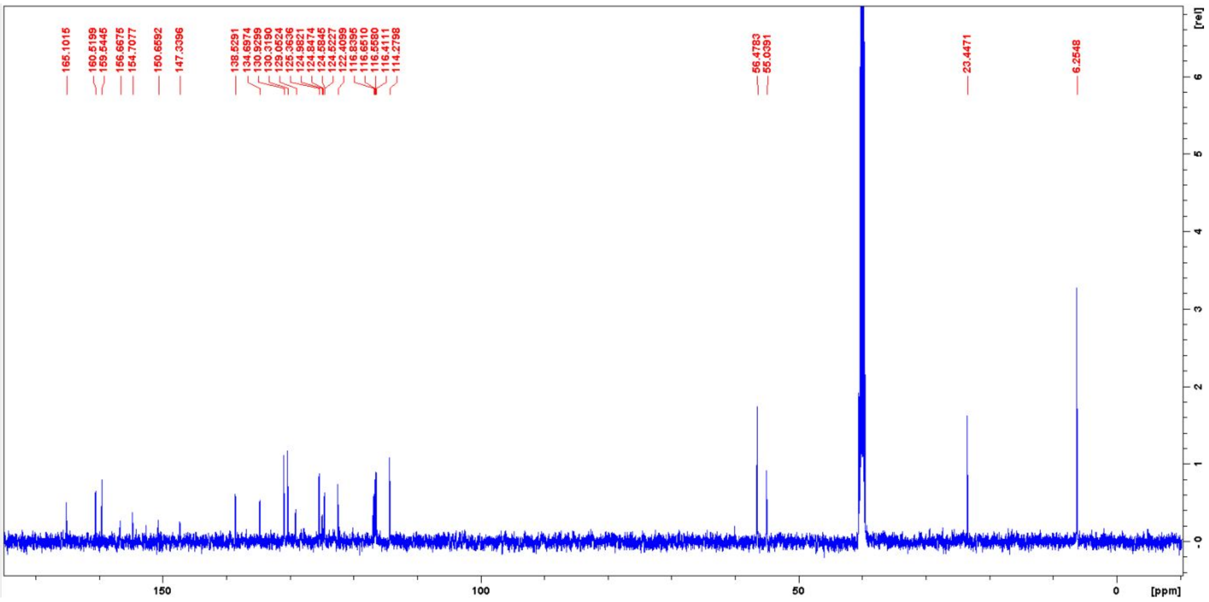
165.1015
160.5199
159.5445
156.6675
154.7077
150.6592
147.3396
138.5291
134.6974
130.9299
130.3190
129.0524
125.3636
124.9821
124.8474
124.5845
124.5227
122.4099
116.8395
116.6510
116.5580
116.4111
114.2798
56.4783
55.0391
23.4471
6.2548
[rel]
[ppm]

HPLC

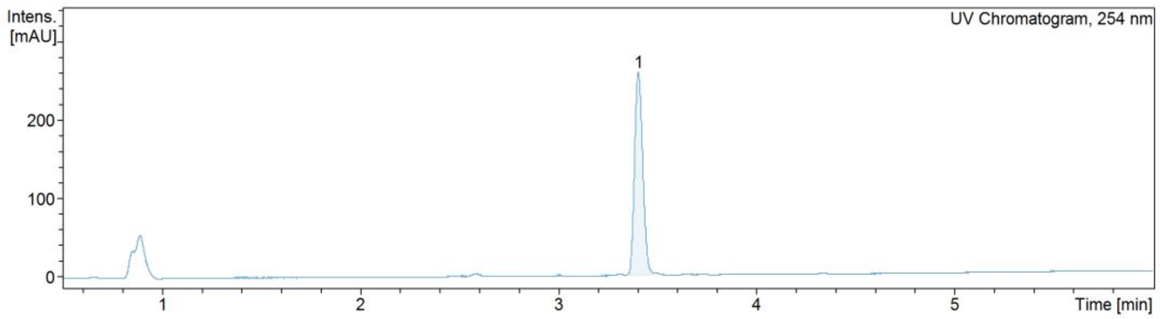

#	RT [min]	Area Frac. %	Chromatogram
1	3.4	100.00	UV Chromatogram, 254 nm
2	3.4	100.00	BPC 74.0000-1601.0000 +, Masses excluded

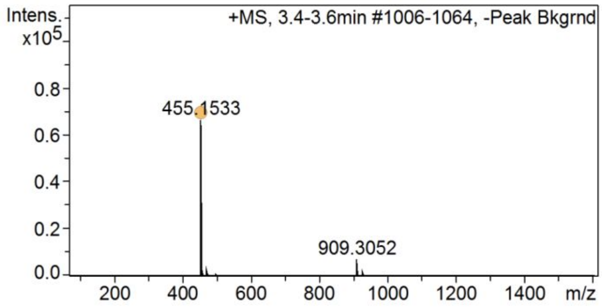

Compound 16

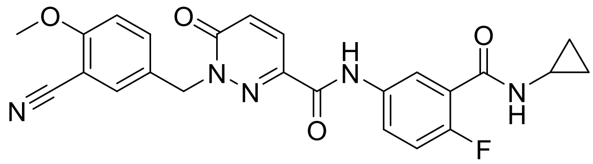

^{1}H NMR

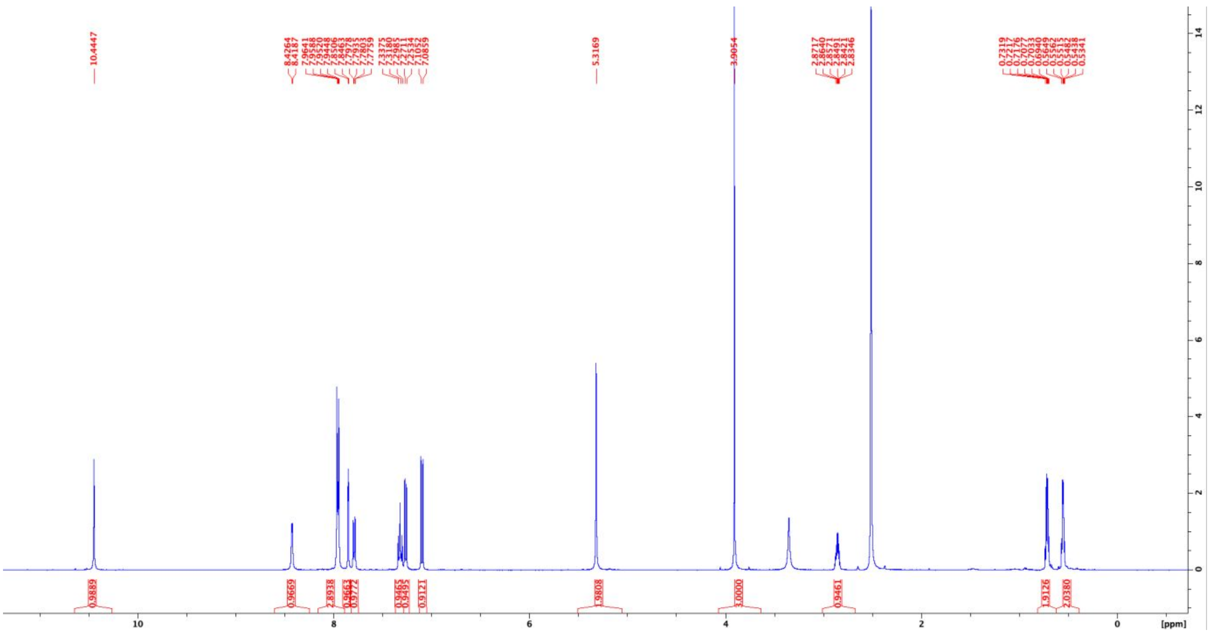

^{13}C NMR

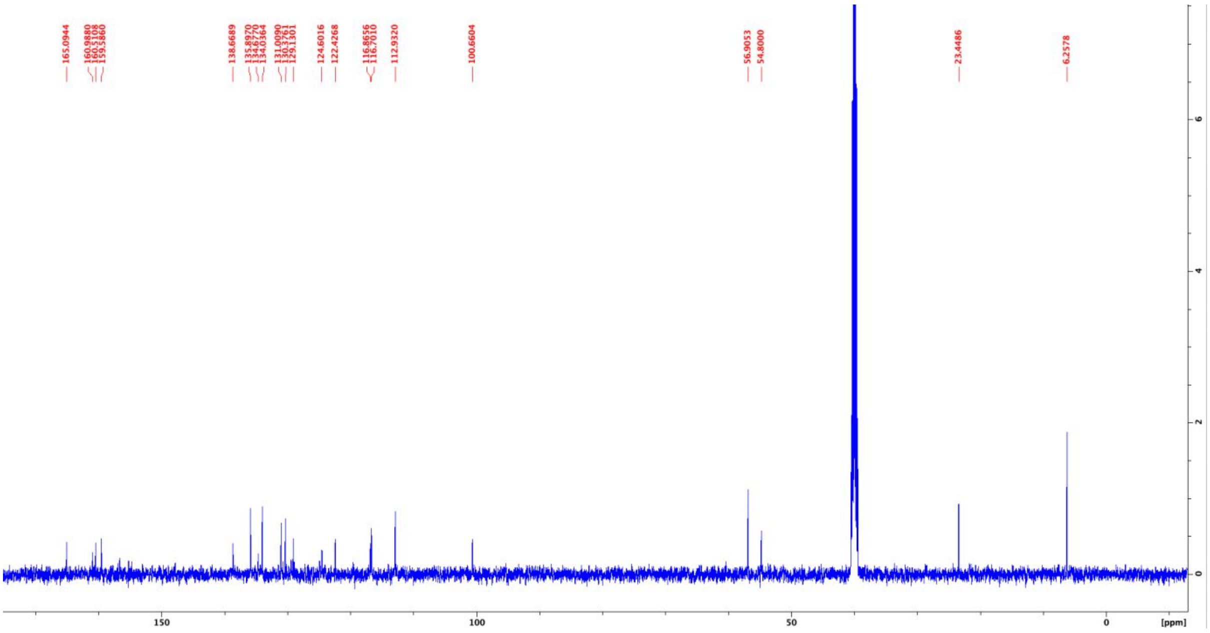

HPLC

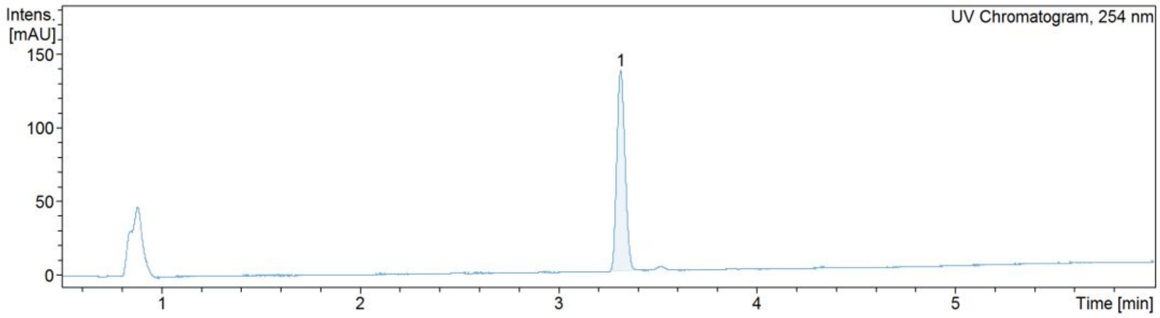

#	RT [min]	Area Frac. %	Chromatogram
1	3.3	100.00	UV Chromatogram, 254 nm
2	3.3	100.00	BPC 75.0000-1601.0000 +, Masses excluded

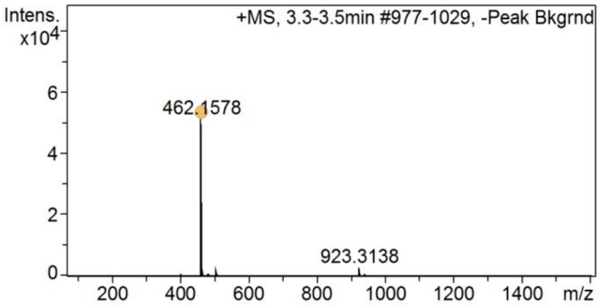

Compound 17

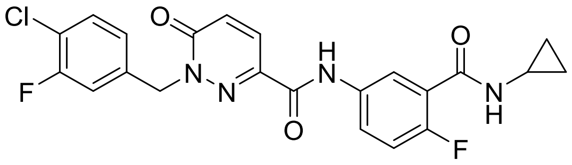

^{1}H NMR

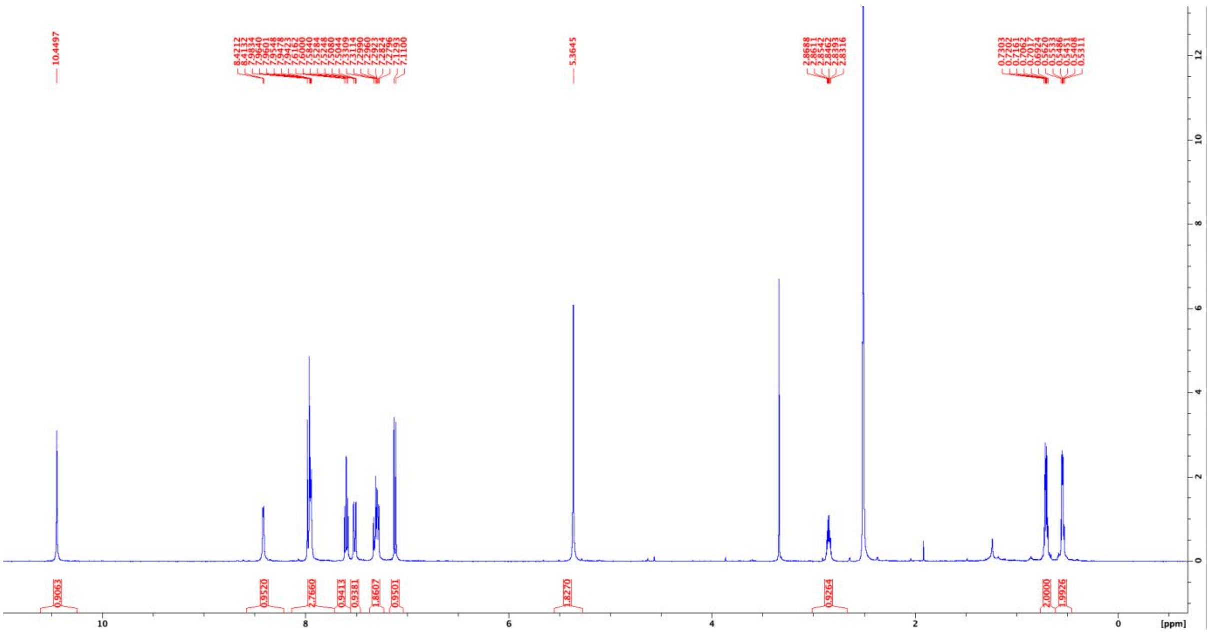

^{13}C NMR

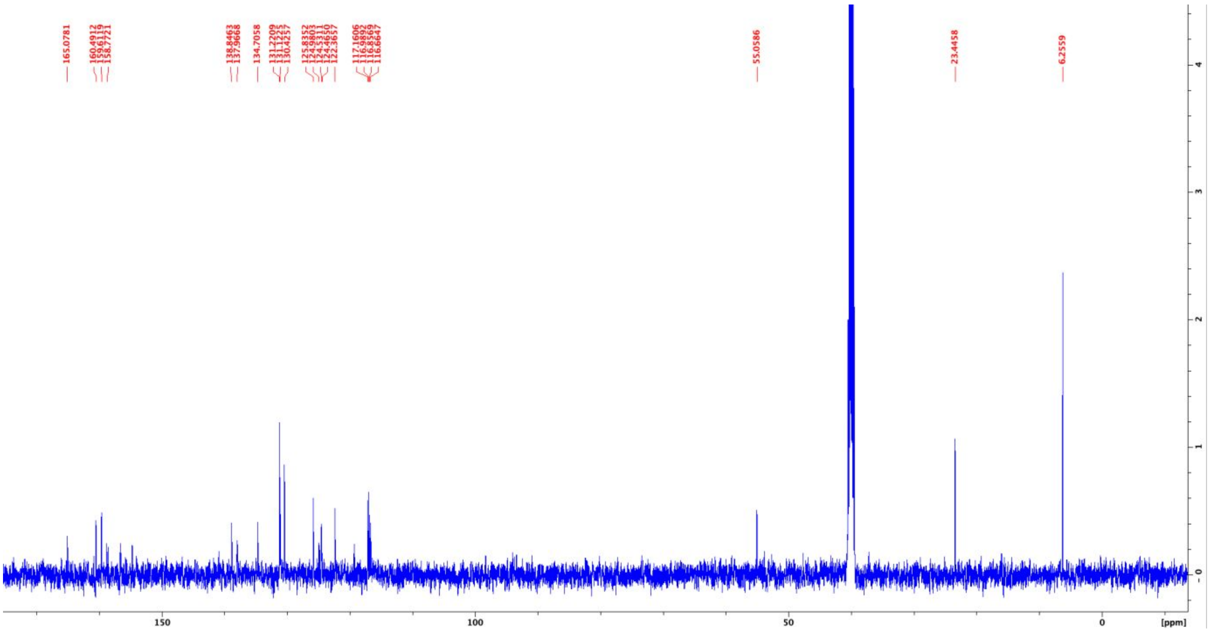

HPLC

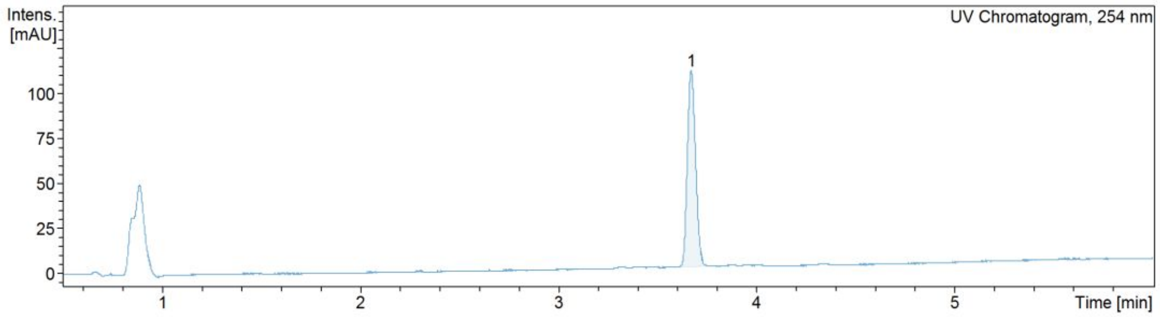

#	RT [min]	Area Frac. %	Chromatogram
1	3.7	100.00	UV Chromatogram, 254 nm
2	3.7	100.00	BPC 75.0000-1601.0000 +, Masses excluded

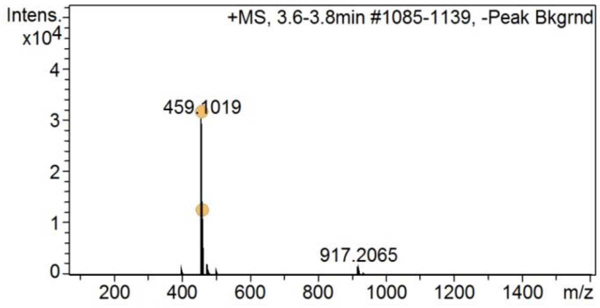

Compound 18

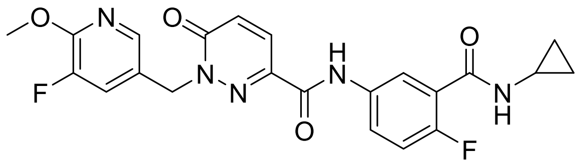

^{1}H NMR

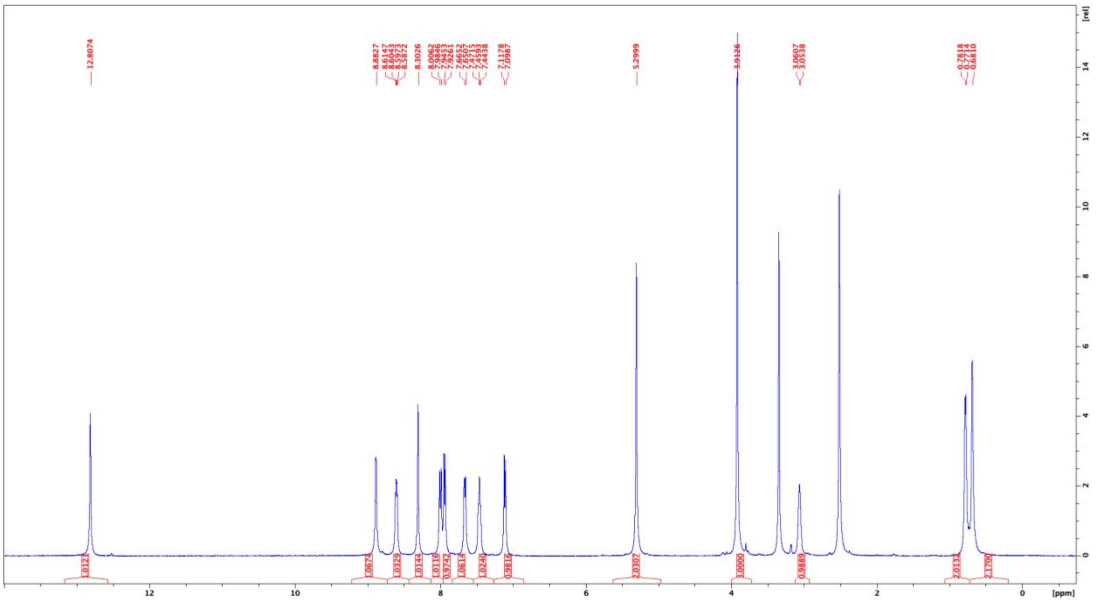
12.8074
8.8827
8.6147
8.6043
8.5973
8.5872
8.3026
8.0062
7.9846
7.9453
7.9261
7.6652
7.6507
7.4715
7.4593
7.4438
7.1178
7.0987
5.2999
3.9126
3.0607
3.0538
0.7818
0.7714
0.6810
[rel]
[ppm]

^{13}C NMR

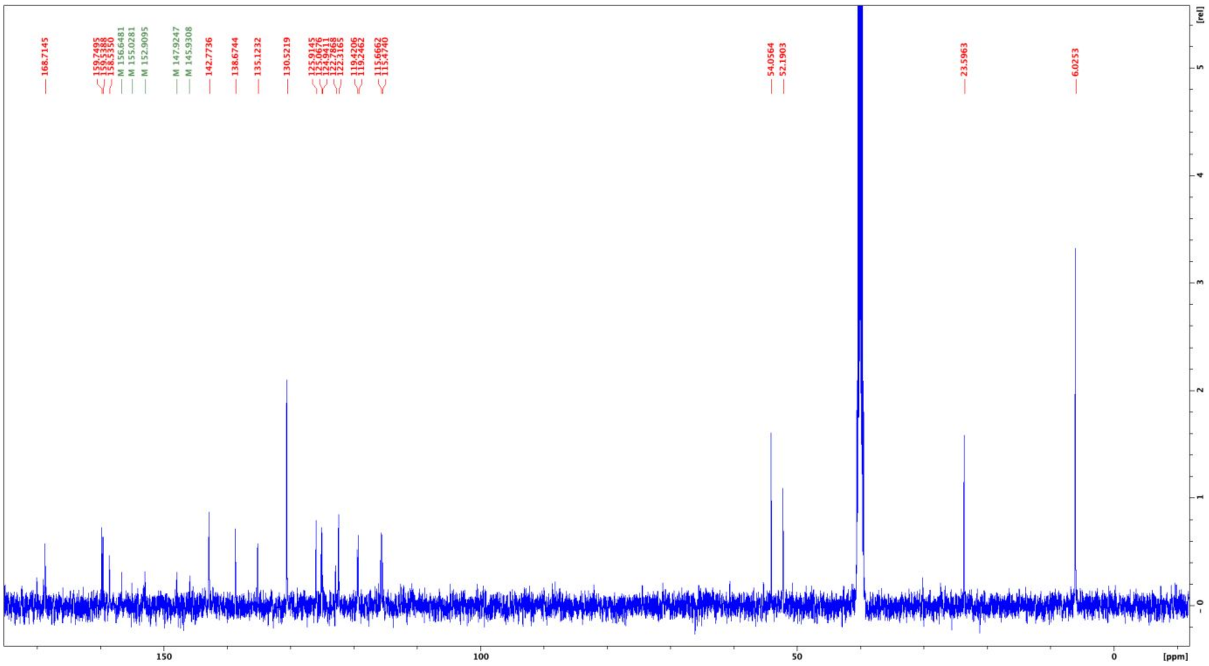
168.7145
159.7495
159.5388
158.5350
M 156.6481
M 155.0281
M 152.9095
M 147.9247
M 145.9308
142.7736
138.6744
135.1232
130.5219
125.9145
125.0676
124.9411
122.7868
122.3165
119.4206
119.2462
115.6662
115.4740
54.0564
52.1903
23.5963
6.0253
[rel]
[ppm]

HPLC

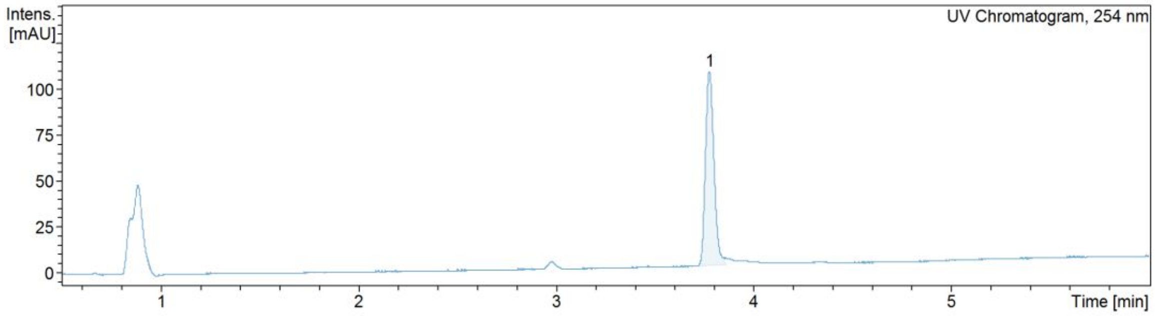

#	RT [min]	Area Frac. %	Chromatogram
1	3.8	100.00	UV Chromatogram, 254 nm
2	3.8	100.00	BPC 74.0000-1600.0000 +, Masses excluded

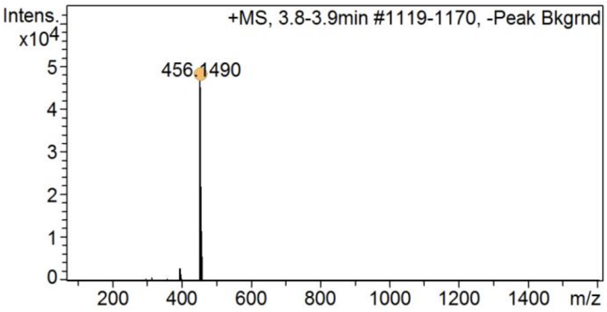

Compound 19

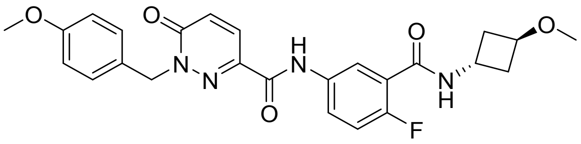

^{1}H NMR

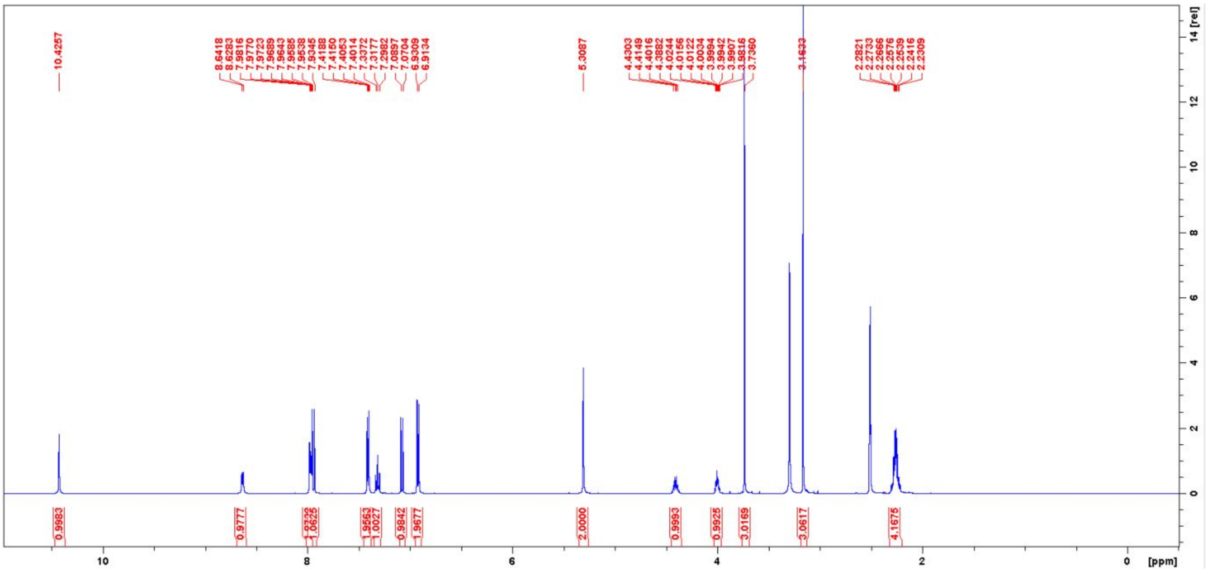

^{13}C NMR

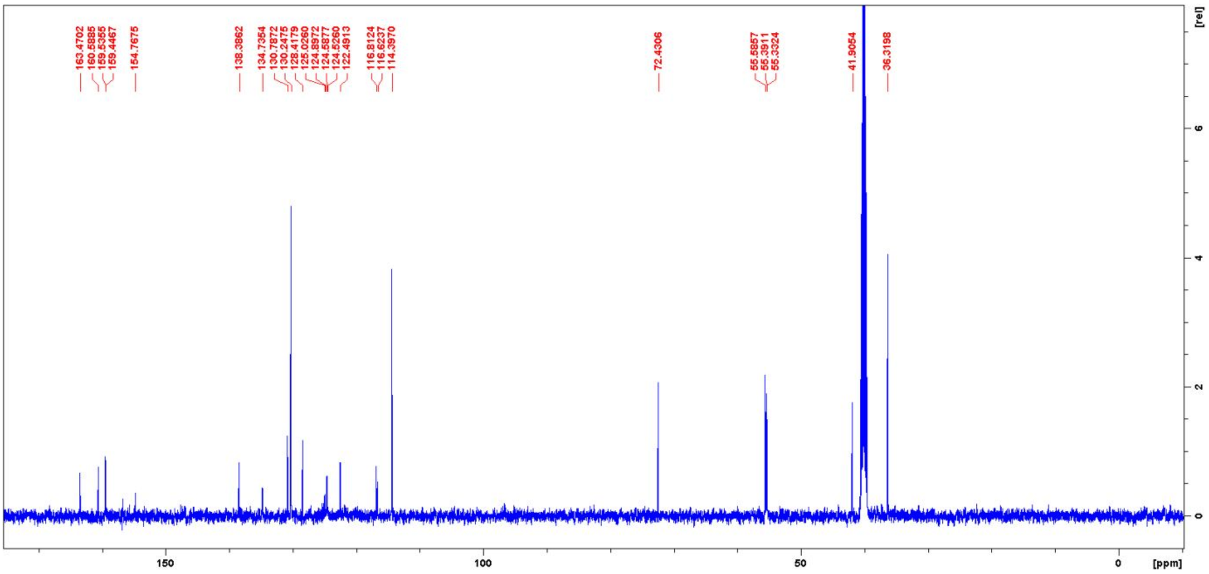

HPLC

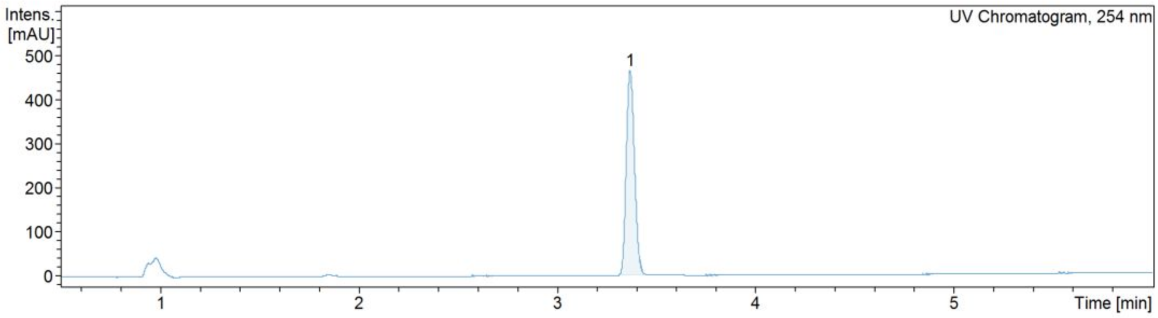

#	RT [min]	Area Frac. %	Chromatogram
1	3.4	100.00	UV Chromatogram, 254 nm
2	3.4	100.00	BPC 74.0000-1601.0000 +, Masses excluded

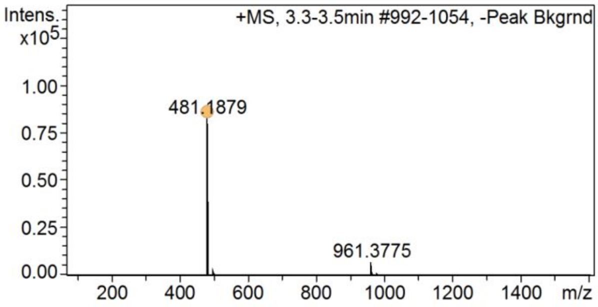

Compound 20

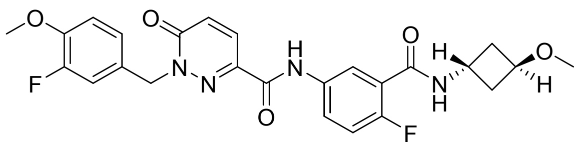

^{1}H NMR

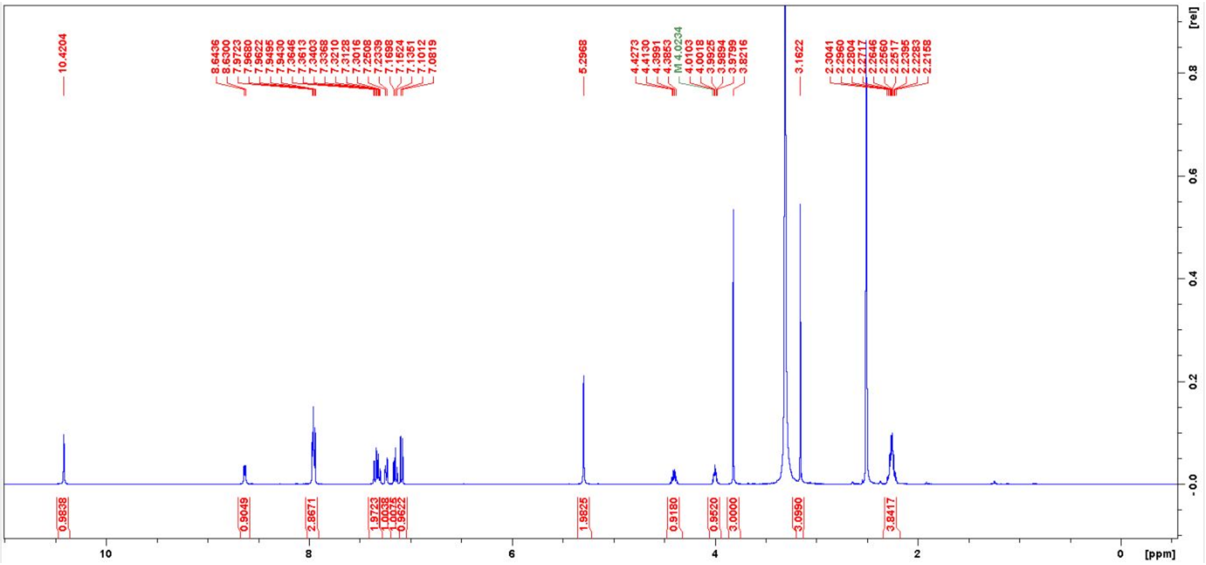

^{13}C NMR

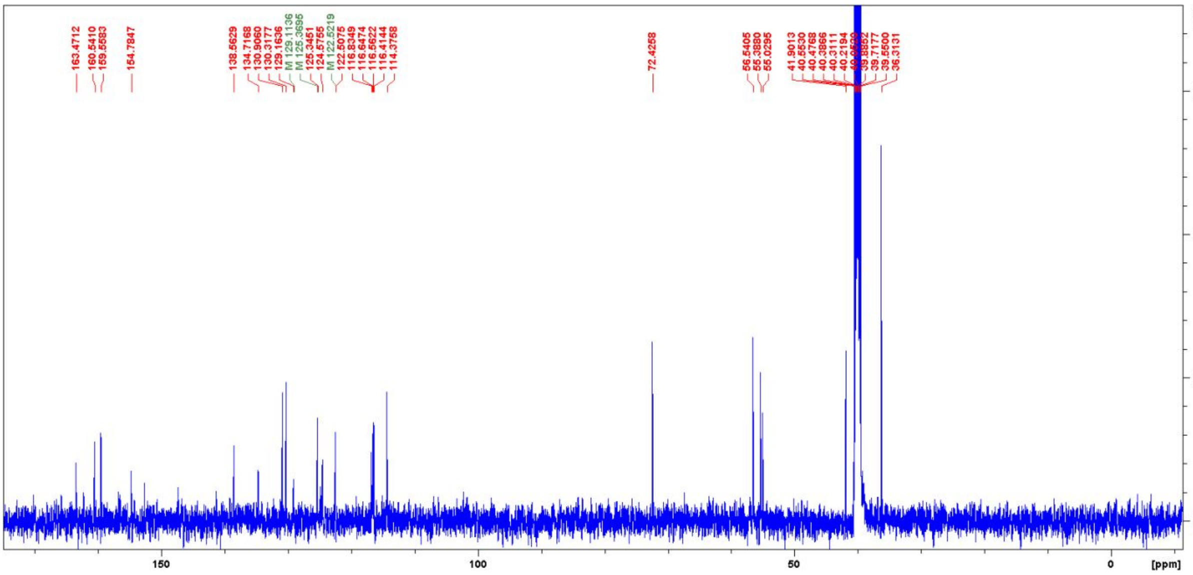

HPLC

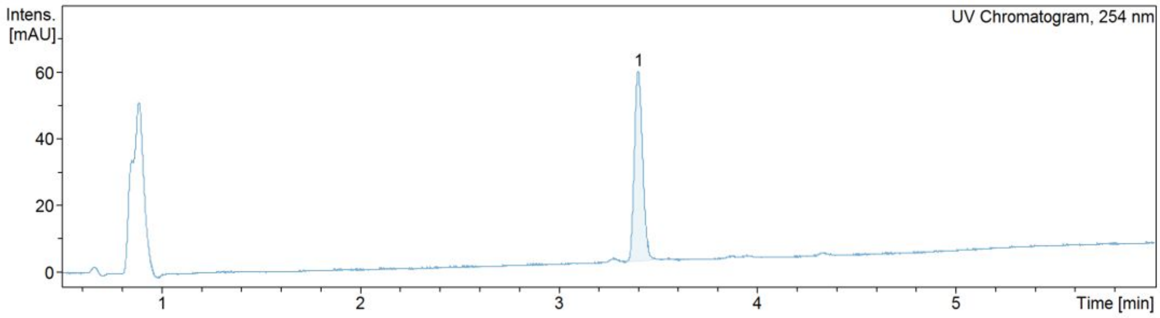

#	RT [min]	Area Frac. %	Chromatogram
1	3.4	100.00	UV Chromatogram, 254 nm
2	3.4	100.00	BPC 74.0000-1601.0000 +, Masses excluded

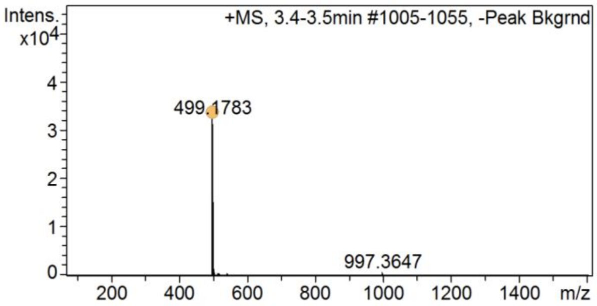

Compound 21

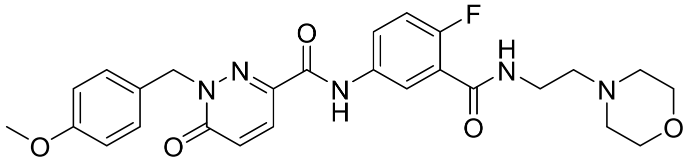

^{1}H NMR

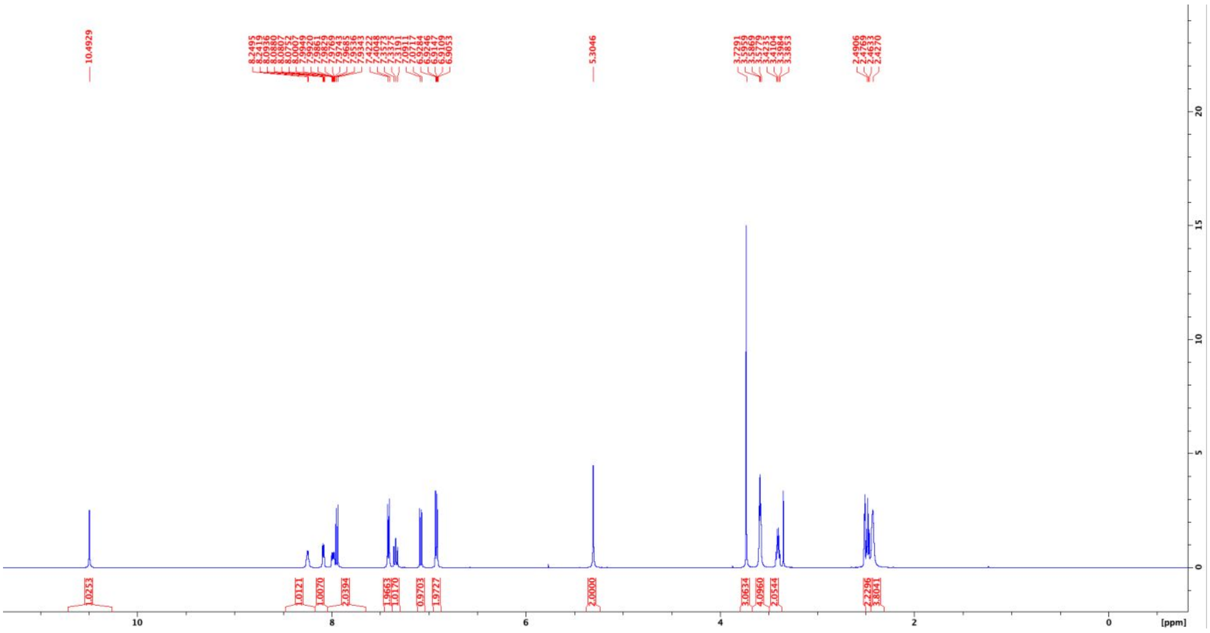

^{13}C NMR

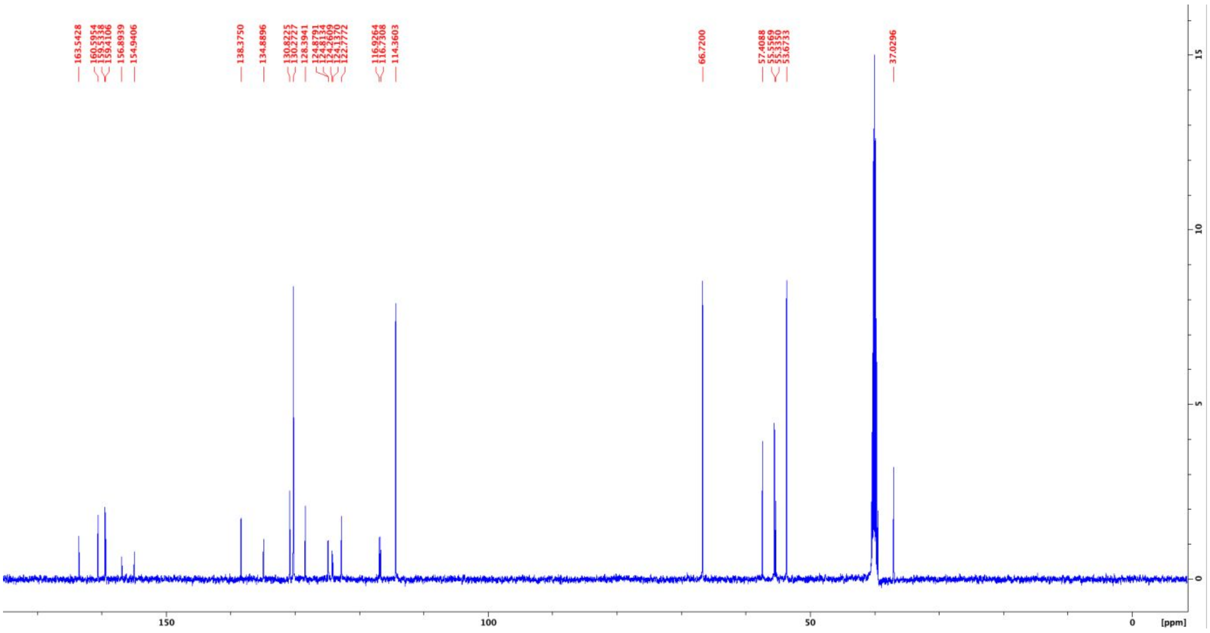

HPLC

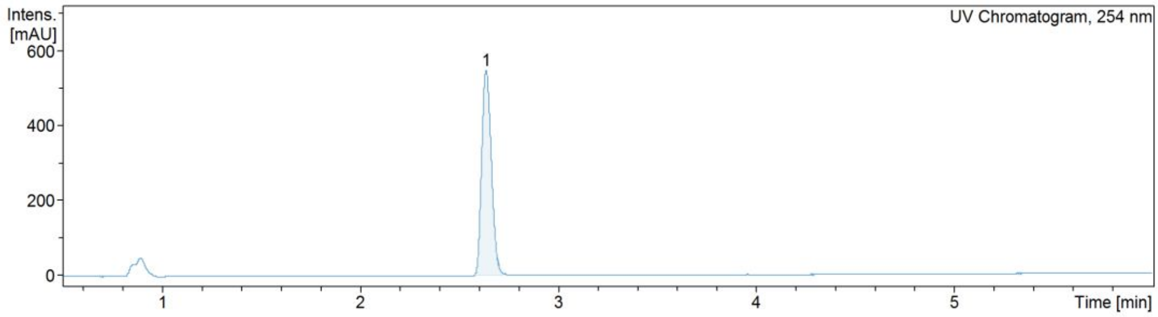

#	RT [min]	Area Frac. %	Chromatogram
1	2.6	100.00	UV Chromatogram, 254 nm
2	2.7	100.00	BPC 75.0000-1601.0000 +, Masses excluded

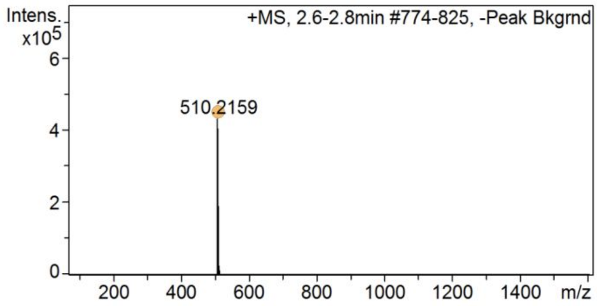

Compound 22

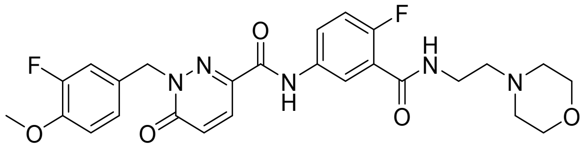

^{1}H NMR

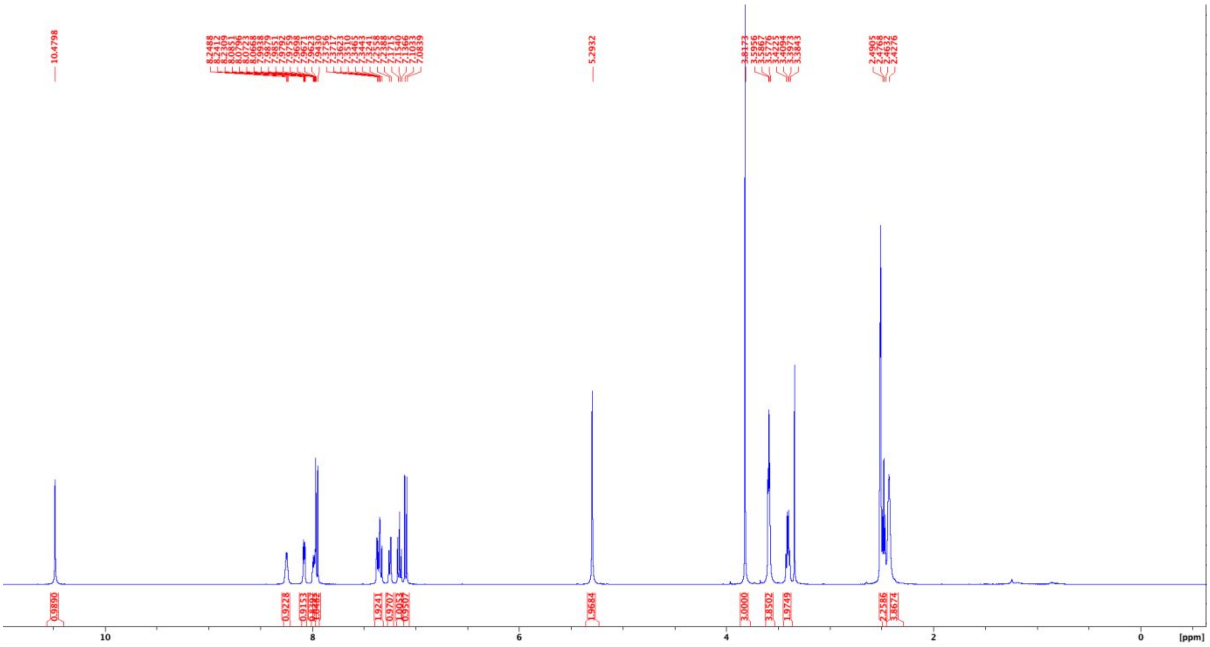
10.4798
5.2932
0.9890
0.9228
1.9241
1.9684
3.0000
3.8502
1.9749
2.2586
3.8674
10
8
6
4
2
0
[ppm]

^{13}C NMR

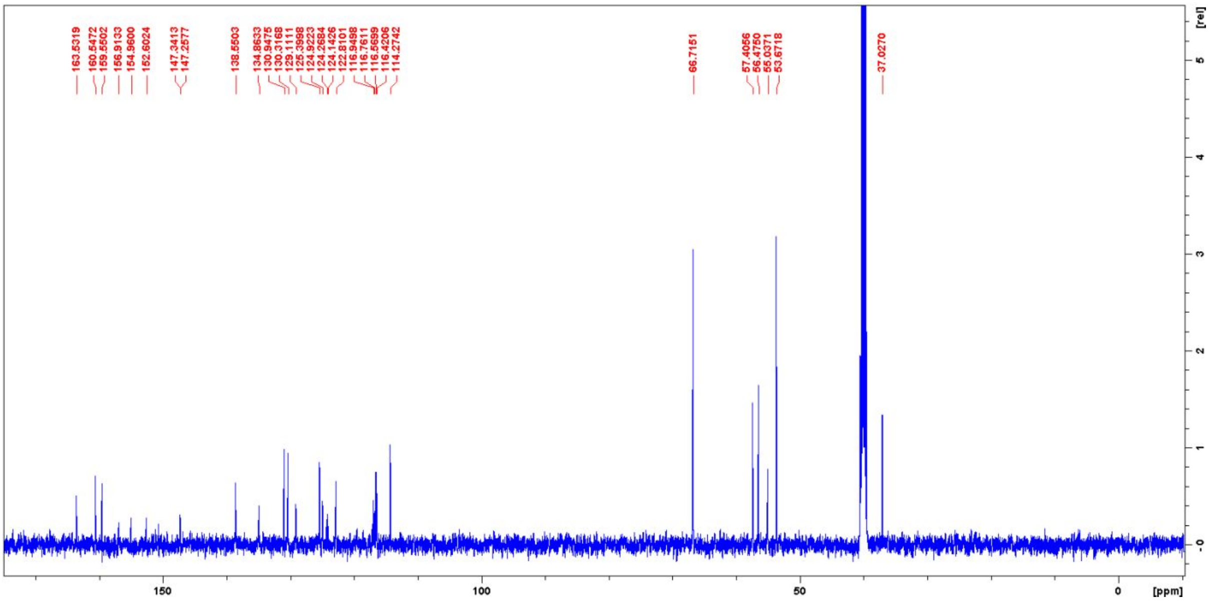
163.5319
160.5472
159.5502
156.9133
154.9600
152.6024
147.3413
147.2577
138.5503
134.8633
130.9475
130.3168
129.1111
125.3998
124.9223
124.2684
124.1426
122.8101
116.9498
116.7611
116.5699
116.4206
114.2742
66.7151
57.4056
56.4750
55.0371
53.6718
37.0270
150
100
50
0
[ppm]
[rel]

HPLC

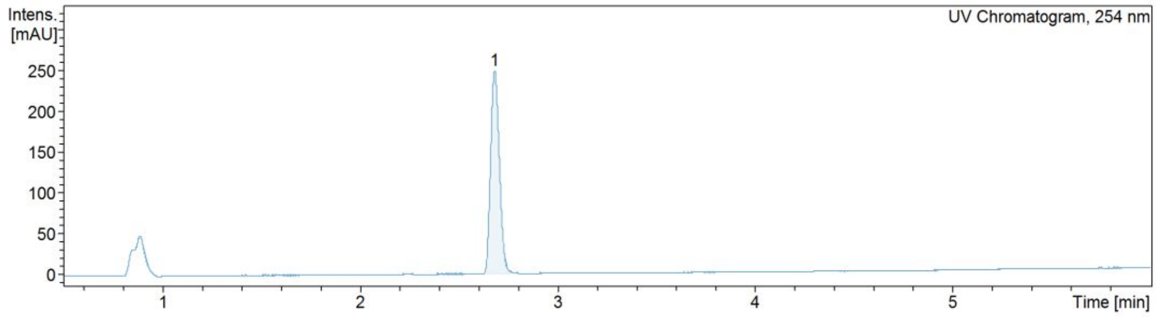

#	RT [min]	Area Frac. %	Chromatogram
1	2.7	100.00	UV Chromatogram, 254 nm
2	2.7	100.00	BPC 74.0000-1600.0000 +, Masses excluded

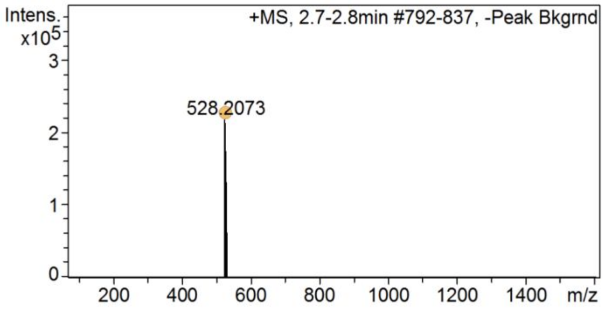

Compound 23

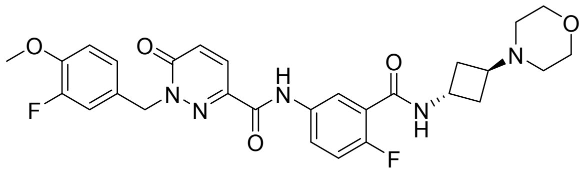

^{1}H NMR

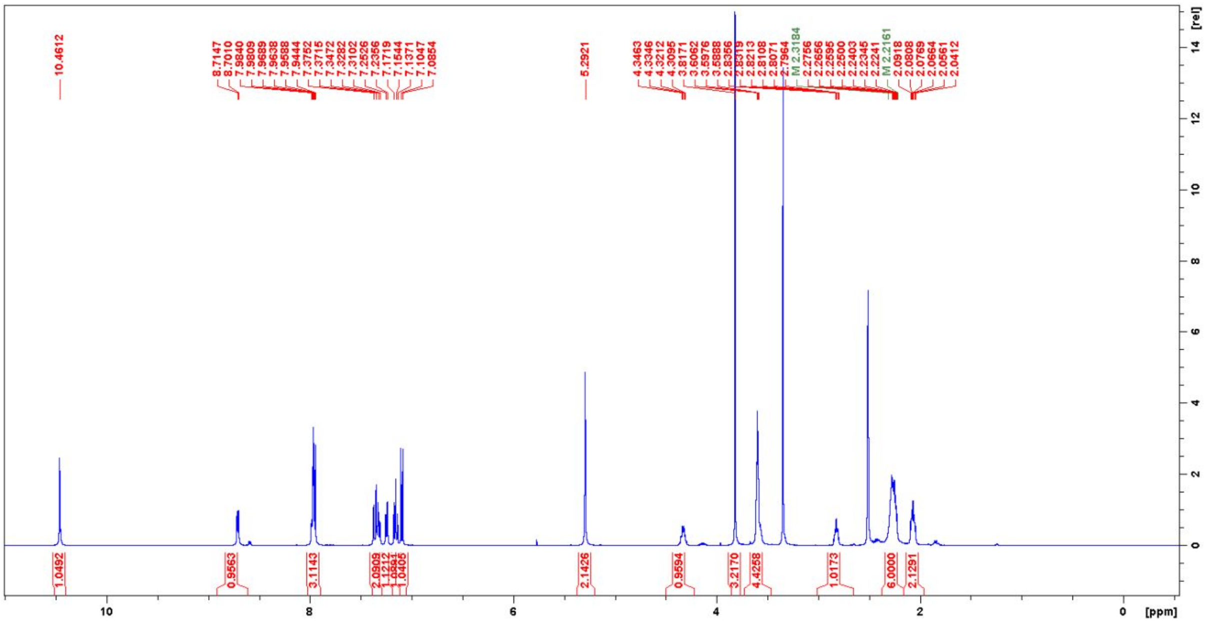

^{13}C NMR

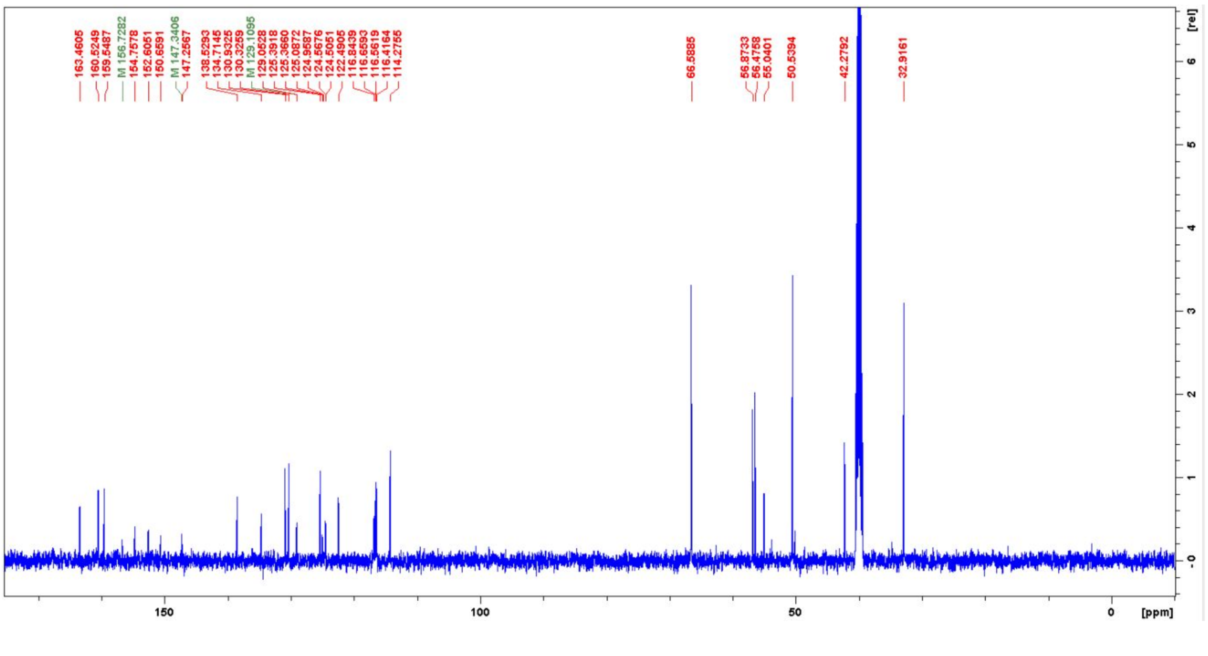

HPLC

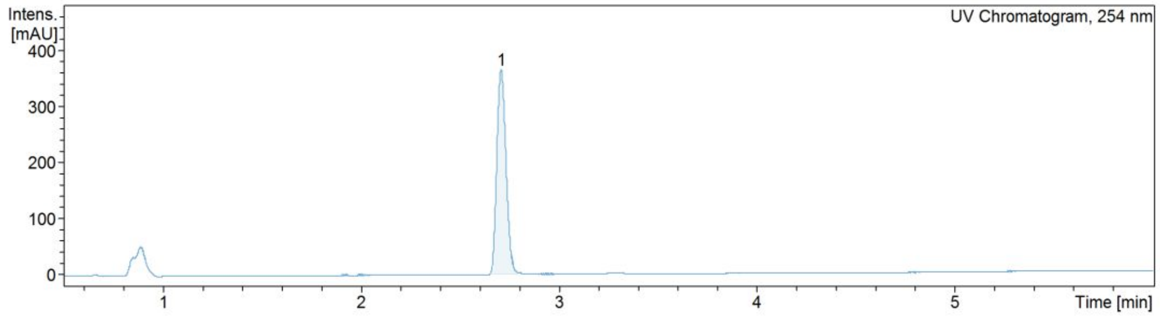

#	RT [min]	Area Frac. %	Chromatogram
1	2.7	100.00	UV Chromatogram, 254 nm
2	2.7	100.00	BPC 74.0000-1601.0000 +, Masses excluded

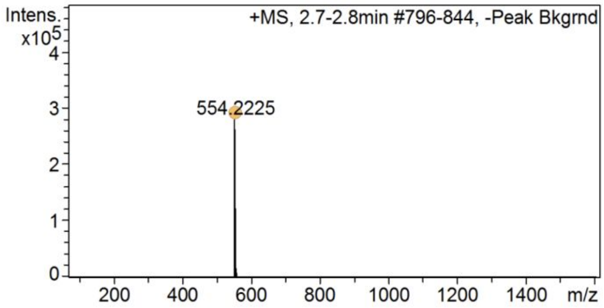

Compound 24

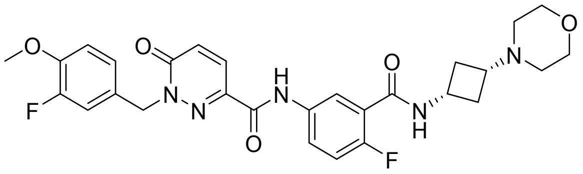

^{1}H NMR

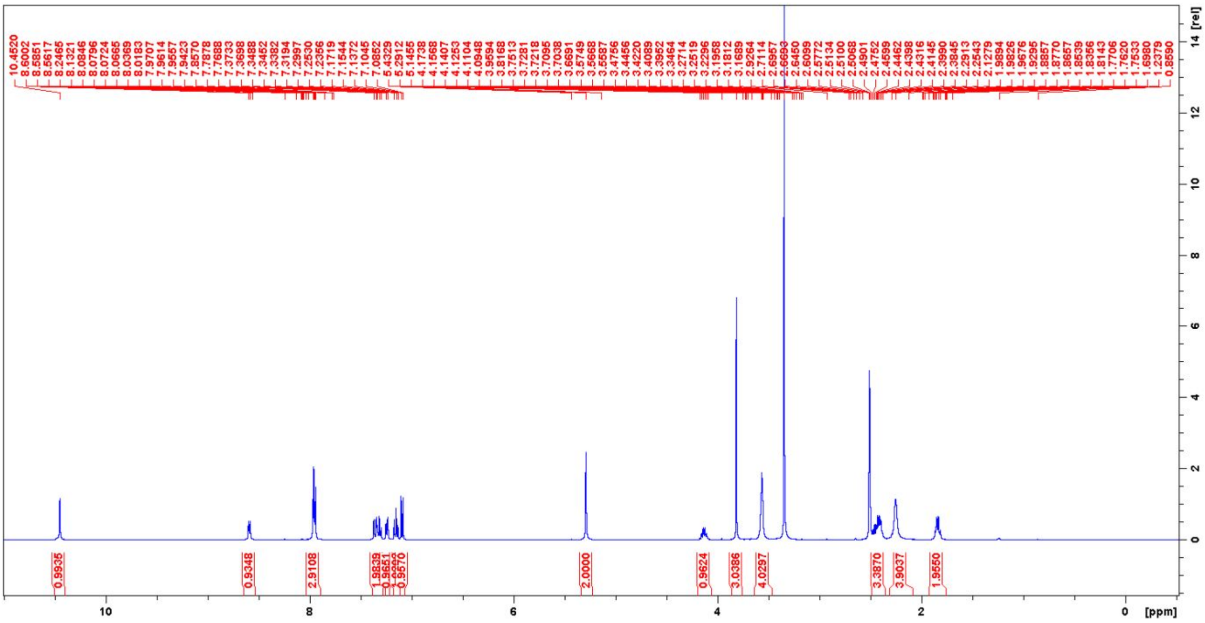
[rel]
[ppm]

^{13}C NMR

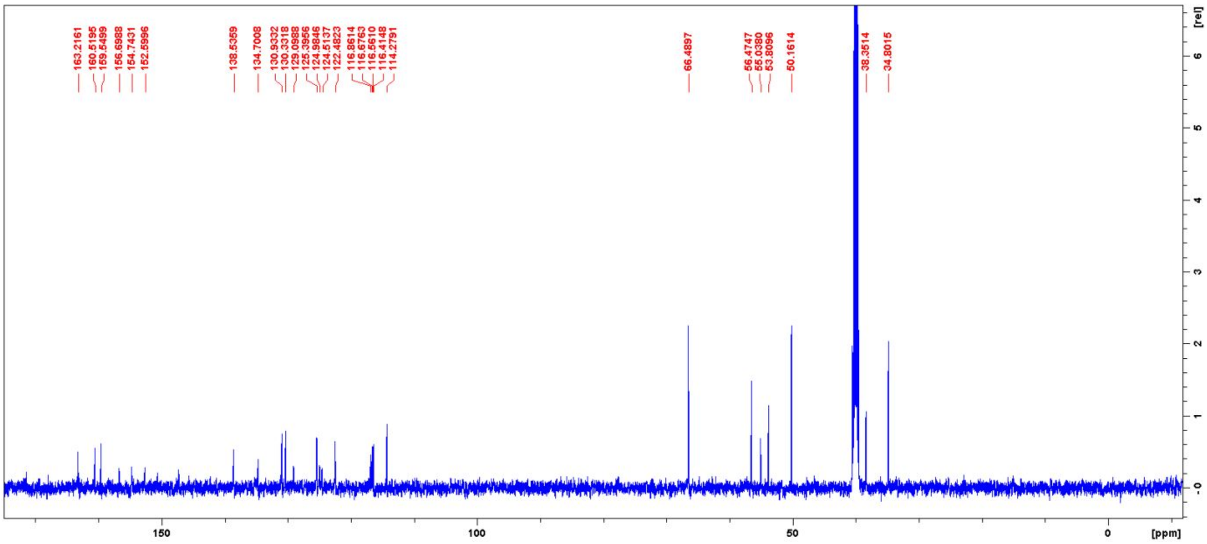
163.2161
160.5195
159.5499
156.6988
154.7431
152.5996
138.5359
134.7008
130.9332
130.3318
129.0988
125.3956
124.9846
124.5137
122.4823
116.8614
116.6763
116.5610
116.4148
114.2791
66.4897
56.4747
55.0380
53.8096
50.1614
38.3514
34.8015
[rel]
[ppm]

HPLC

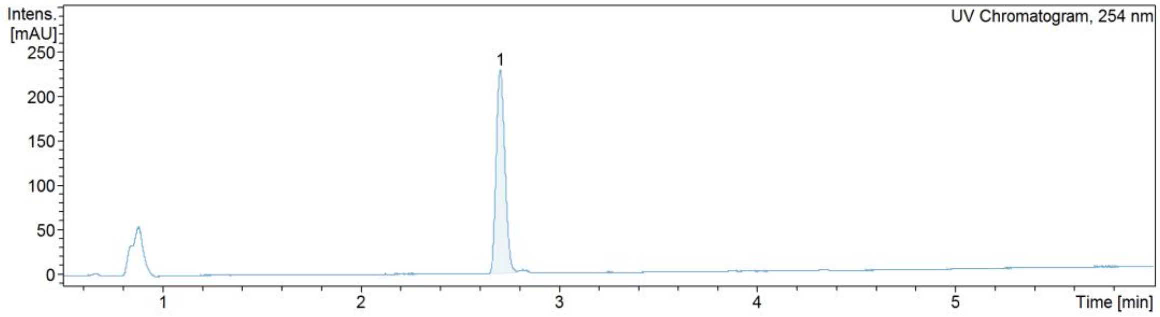

#	RT [min]	Area Frac. %	Chromatogram
1	2.7	100.00	UV Chromatogram, 254 nm
2	2.7	100.00	BPC 74.0000-1601.0000 +, Masses excluded

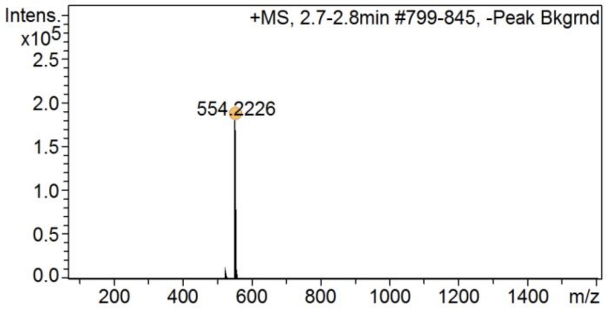

Compound 25

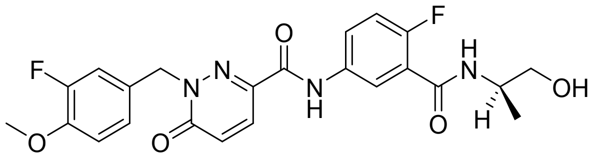

^{1}H NMR

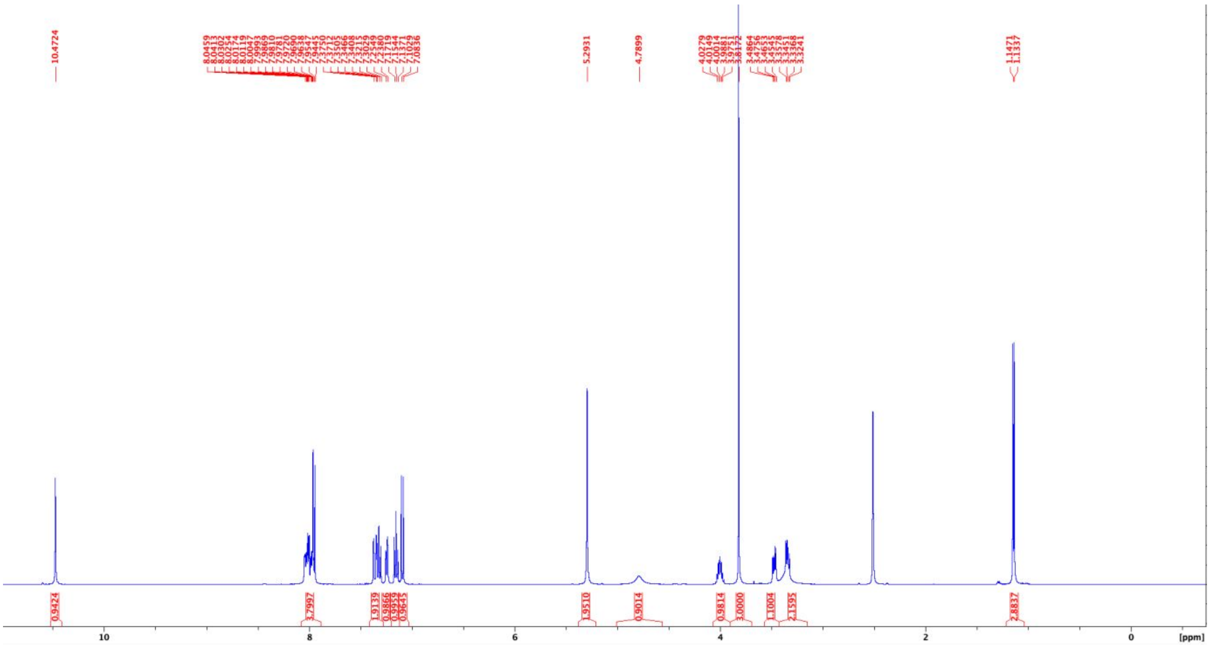
10.4724
8.0459
8.0413
8.0302
8.0254
8.0174
8.0119
8.0047
7.9993
7.9869
7.9810
7.9781
7.9720
7.9690
7.9638
7.9547
7.9445
7.3750
7.3712
7.3505
7.3466
7.3408
7.3215
7.3029
7.2549
7.2380
7.1719
7.1544
7.1371
7.1029
7.0836
5.2931
4.7899
4.0279
4.0149
4.0014
3.9881
3.9751
3.8172
3.4864
3.4756
3.4653
3.4545
3.3578
3.3451
3.3368
3.3241
1.1471
1.1337
0.9424
3.7997
1.9139
0.9866
0.9645
1.9510
0.9014
0.9814
3.0000
1.1004
2.1595
2.8837
10
8
6
4
2
0
[ppm]

^{13}C NMR

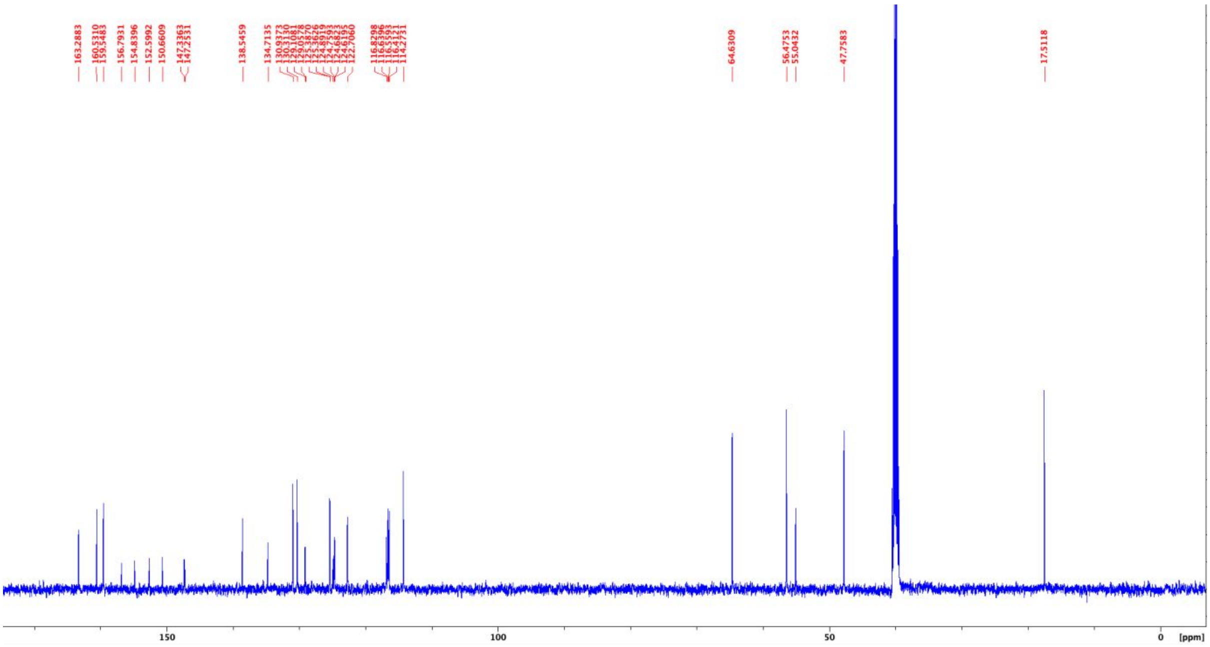
163.2883
160.5310
159.5483
156.7931
154.8396
152.5992
150.6609
147.3363
147.2531
138.5459
134.7135
130.9373
130.3130
129.1081
129.0578
125.3870
125.3626
124.8919
124.7593
124.6823
124.6195
122.7060
116.8298
116.6396
116.5593
116.4121
114.2731
64.6309
56.4753
55.0432
47.7583
17.5118
150
100
50
0
[ppm]

HPLC

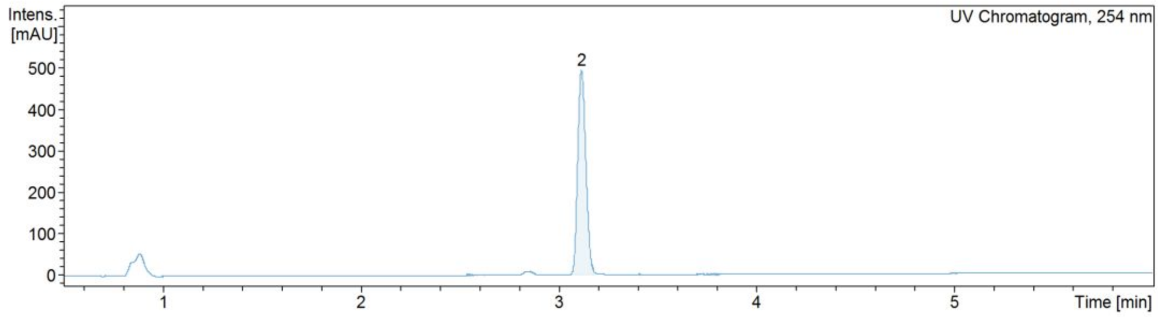

#	RT [min]	Area Frac. %	Chromatogram
1	2.9	6.87	BPC 74.0000-1601.0000 +, Masses excluded
2	3.1	100.00	UV Chromatogram, 254 nm
3	3.1	93.13	BPC 74.0000-1601.0000 +, Masses excluded

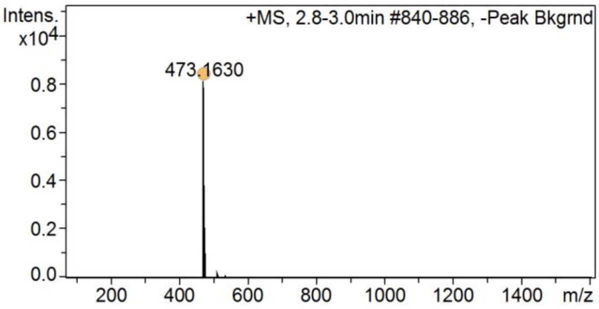

Compound 26

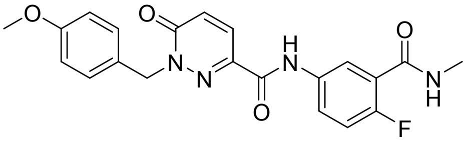

^{1}H NMR

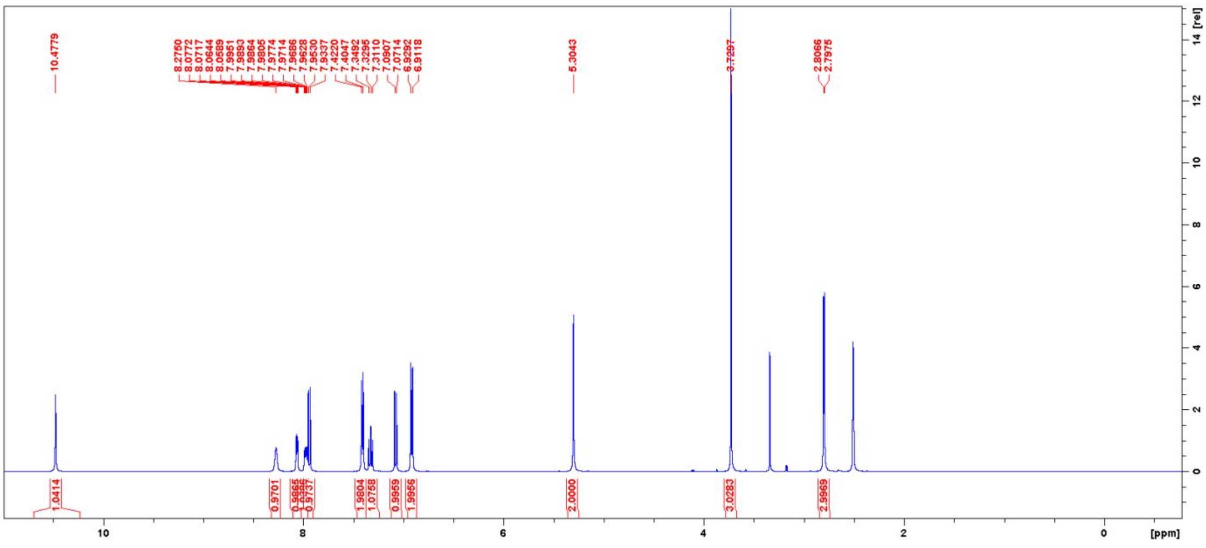

^{13}C NMR

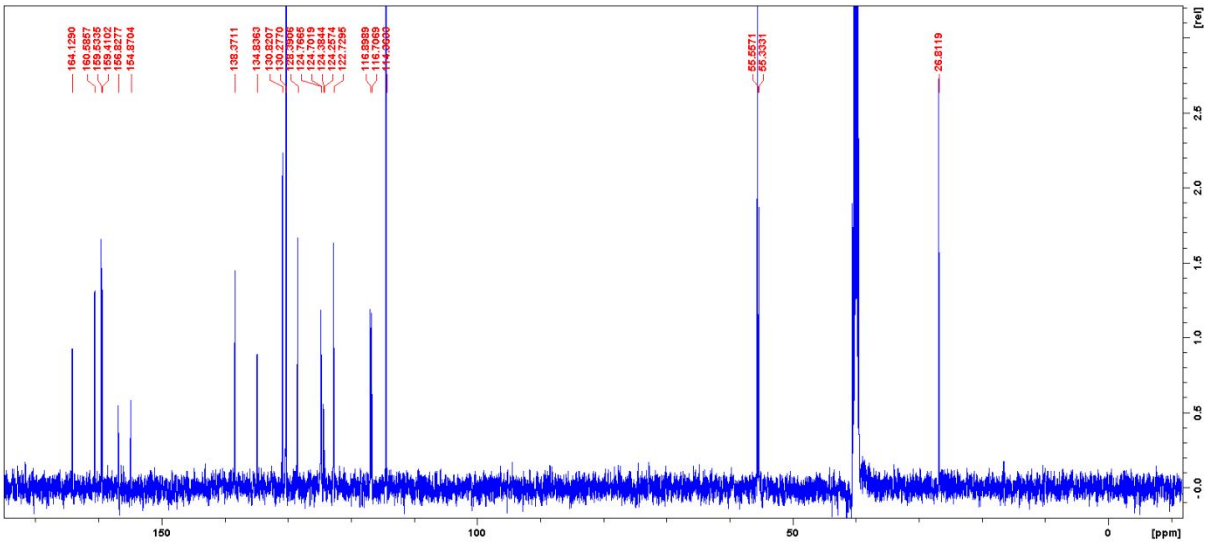

HPLC

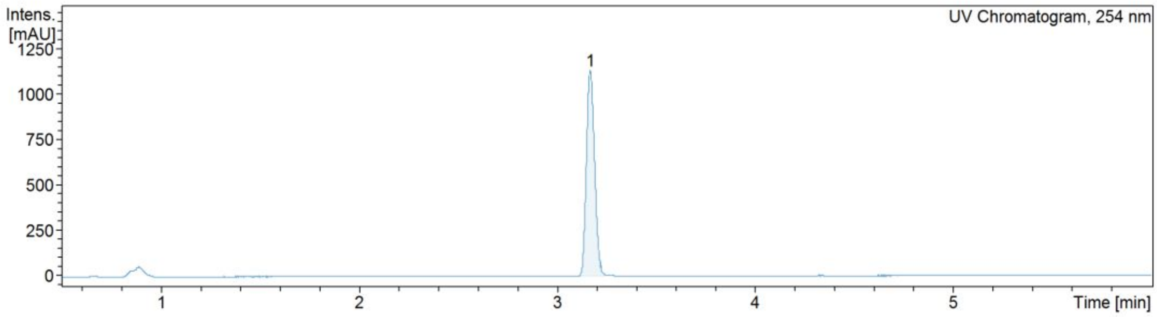

#	RT [min]	Area Frac. %	Chromatogram
1	3.2	100.00	UV Chromatogram, 254 nm
2	3.2	100.00	BPC 75.0000-1601.0000 +, Masses excluded

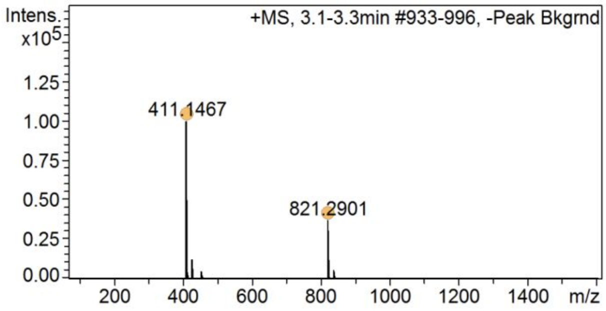

Compound 27

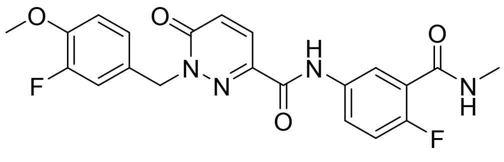

^{1}H NMR

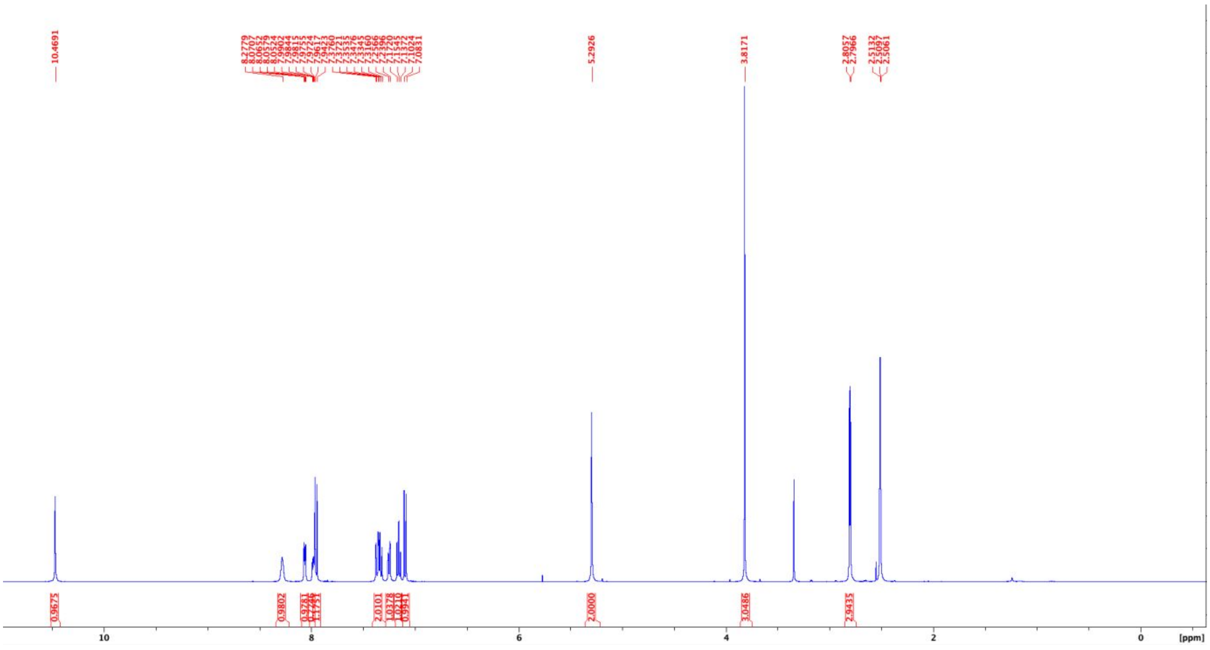

^{13}C NMR

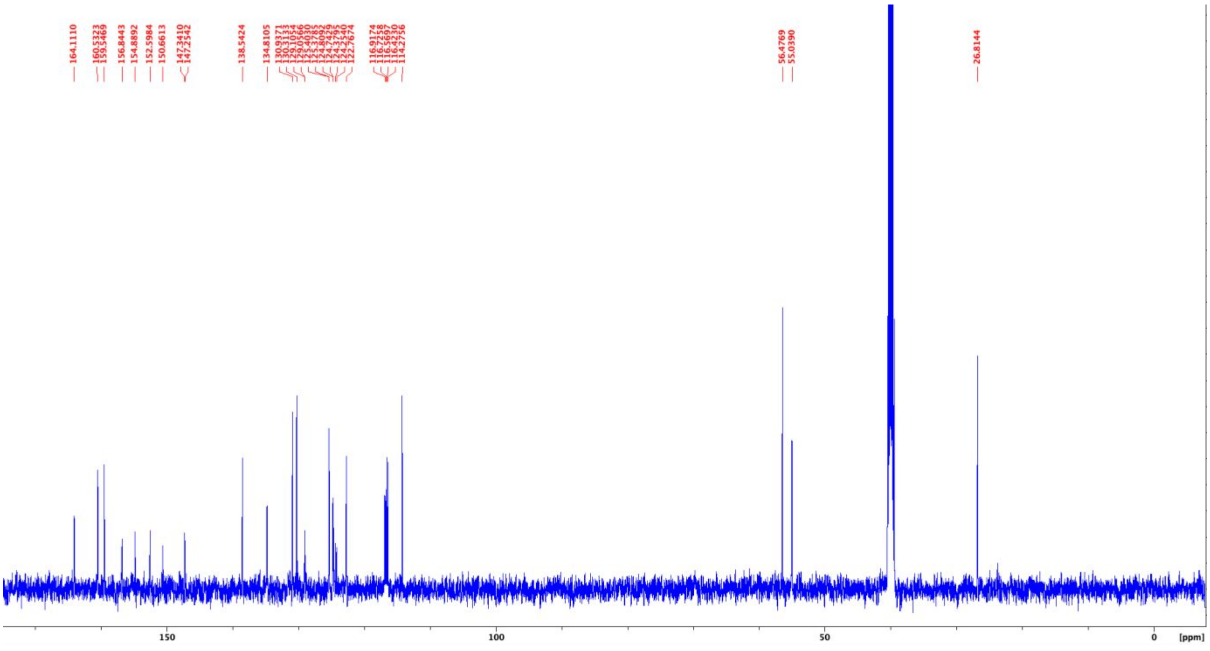

HPLC

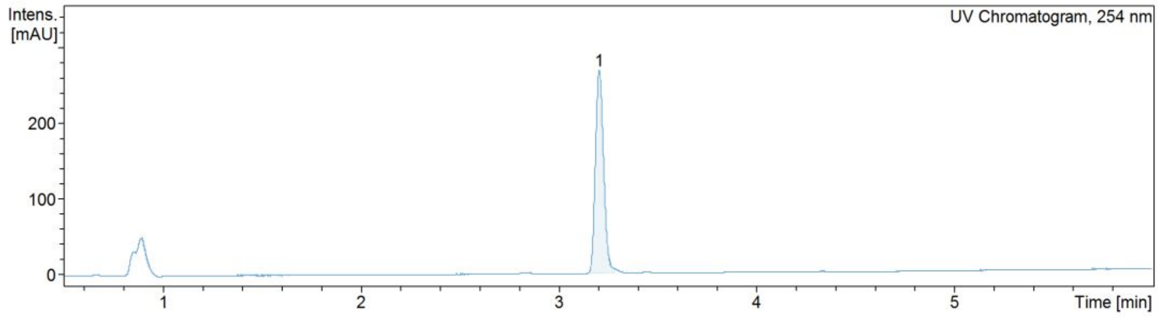

#	RT [min]	Area Frac. %	Chromatogram
1	3.2	100.00	UV Chromatogram, 254 nm
2	3.2	100.00	BPC 75.0000-1601.0000 +, Masses excluded

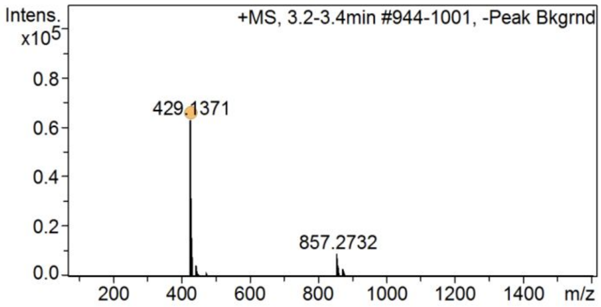

***In vitro* assays**

***T. cruzi* and human proteasome assays**

High throughput screen was carried out as previously described:

Zmuda, F.; Sastry, L.; Shepherd, S. M.; Jones, D.; Scott, A.; Craggs, P. D.; Cortes, A.; Gray, D. W.; Torrie, L. S.; De Rycker, M. Identification of Novel Trypanosoma cruzi Proteasome Inhibitors Using a Luminescence-Based High-Throughput Screening Assay. *Antimicrobial Agents and Chemotherapy*, 2019, 63(9), 1 – 15

Potency data for compounds **5** – **27** was generated using a modified version of this procedure:

The T. cruzi proteasome was partially purified as described in the above reference and Human 20S proteasome was purchased from Boston Biochem (now R&D Systems, catalogue number E-360). Titrations of test compounds were prepared from 10 mM stocks in 100% DMSO, using a Tecan Fluent to titrate over 11 points in Labcyte Echo-compatible Greiner Bio-One 384 well polypropylene microplates. Subsequently, 100 nl of test compound titrations were transferred to black Greiner Bio-One low volume 384 well microplates using a Labcyte Echo acoustic dispenser.

T. cruzi proteasome was diluted to a concentration of 0.94 μg/ml in an assay buffer comprising 50 mM HEPES pH 7.5, 150 mM sodium chloride, 5% Glycerol, 1 mM dithiothreitol, 1 mM CHAPS in deionised, filtered water (all chemicals purchased from Sigma Aldrich). Human 20S proteasome was diluted to a concentration of 1.75 μg/ml in the aforementioned assay buffer. These enzyme solutions were prepared immediately prior to assay initiation and stored at 4 °C before use.

To monitor the Chymotrypsin-like activity of the T. cruzi and Human 20S proteasome proteins a self-quenched, Rhodamine 110-labelled peptide with the sequence Suc-LLVY-Rho-110-DPro was used (prepared by Cambridge Research Biochemicals. Suc is succinyl, while DPro refers to D-proline). The peptide was initially prepared from solid as a 10 mM solution in 100% DMSO and stored at -20 °C. Prior to use in the assay, an aliquot was thawed at room temperature and then diluted to 12.5 μM in assay buffer, which is a concentration equivalent to twofold Km. For use with the Human 20S proteasome, the Suc-LLVY-Rho-110-DPro

peptide was diluted in assay buffer to a concentration equivalent to twofold Km, which for this enzyme was 2 μM. The peptide solutions were protected from light and stored at 4 °C before use.

A Multidrop Micro (Thermo Fisher), using a low volume dispense cassette, was used to add the assay solutions to the test compounds in the low volume 384-well microplates. Prior to addition of proteasome solution, 5 μl of assay buffer was added to one column (containing 100 nl, 100% DMSO) to act as no enzyme control to mimic full inhibition. Subsequently, 5 μl of proteasome solution (either T. cruzi or Human 20S) was added to all wells on the test microplate apart from the column containing 5 μl assay buffer. The microplate was then spun using a suitable bench top centrifuge for 1 minute at 1000 rpm, before incubation at room temperature for 10 mins.

Following the initial incubation, 5 μl of substrate solution was then added to all wells on the test microplate using a Multidrop Micro equipped with a discrete low volume dispense cassette. The test microplates were then spun for 10 seconds at 1000 rpm before being rapidly moved to a multi-mode microplate reader, capable of monitoring fluorescence intensity. The microplate reader used for these studies was a Tecan M1000 Pro, equipped with quad monochromators. Fluorescence intensity was measured by excitation at 485 ± 5 nm and detection of emission at 530 ± 5 nm. A series of fluorescence intensity measurements were taken every 30 seconds for 30 minutes.

Subsequently, the fluorescence intensity measurements (relative fluorescence units, RFU) were fit to a simple linear regression model (y = A + Bx, where y refers to the fluorescence intensity and x refers to the time in seconds) to determine the initial rate of reaction. The initial rate for every compound concentration tested was then fit a 4-parameter logistic model: y = (A + (B - A)) / (1 + (x / C)D), where y refers to the assay response, x refers to the inhibitor concentration, A and B are the range values (minimum and maximum, respectively), C is the IC50 value and D is the slope. Potency values were then reported as pIC50, which is the negative log of the IC50 (on a molar scale). Data analysis was automated and carried out using the ActivityBase XE software.

***T. cruzi* intracellular assay**

Potency against intracellular *T. cruzi* X10/7 A1 amastigotes was determined as described previously, with as sole modification that the compound treatment duration was increased from 72 h to 96 h:

MacLean, L.; Thomas, J.; Lewis, M. D.; Cotillo, I.; Gray, D.; & De Rycker, M. Development of Trypanosoma cruzi *in vitro* assays to identify compounds suitable for progression in Chagas' disease drug discovery. *PLoS Neglected Tropical Diseases*, **2018**, 12(7), 1 – 22

***T. cruzi* in vitro washout assay**

This assay was run as previously reported:

McGonagle K, Tarver G.J.; Cantizano J.; Cotillo I.; Dodd P.G.; Ferguson L.; Gilbert I.H.; Marco M.; Miles T.J.; Naylor C.; Osuna-Cabello M.; Paterson C.; Read K.D.; Pinto E.G.; Riley J.; Scullion P.; Shishikura Y.; Simeons F.; Stojanovski L.; Svensen N.; Thomas J.; Wyatt P.G.; Manzano P. De Rycker M.; Thomas M.G. Identification and development of a series of disubstituted piperazines for the treatment of Chagas disease. *Eur. J. Med. Chem.*, **2022**, 238, 114421

Mouse microsomal intrinsic clearance.

This assay was conducted as previously described:

Brand, S.; Ko, E. J.; Viayna, E.; Thompson, S.; Spinks, D.; Thomas, M.; Sandberg, L.; Francisco, A. F.; Jayawardhana, S.; Smith, V. C.; Jansen, C.; De Rycker, M.; Thomas, J.; MacLean, L.; Osuna-Cabello, M.; Riley, J.; Scullion, P.; Stojanovski, L.; Simeons, F. R. C.; Epemolu, O.; Shishikura, Y.; Crouch, S. D.; Bakshi, T. S.; Nixon, C. J.; Reid, I. H.; Hill, A. P.; Underwood, T. Z.; Hindley, S. J.; Robinson, S. A.; Kelly, J. M.; Fiandor, J. M.; Wyatt, P. G.; Marco, M.; Miles, T. J.; Read, K. D.; Gilbert, I. H. Discovery and Optimization of 5-Amino-1,2,3-triazole-4-carboxamide Series against Trypanosoma cruzi. **2017**, *60*, 7284-7299

Aqueous Solubility

Test compounds were dissolved in DMSO to give 10 mM solutions. Solubility test samples were prepared by adding a volume (5 µL) of the 10 mM solution to a volume (195 µL) of phosphate buffered saline, pH 7.4 (Sigma-Aldrich, Cat no. P4417, made as per manufacturer's instructions). This solution was then mixed for 24 hours (rotary mixing, 900 rpm, 25°C) excluding light. After mixing, the solubility test samples were filtered to remove any

undissolved material using a proprietary filter (Millipore Multiscreen HTS filter, 96-well format). Samples were then drawn through the filter using vacuum. The filtrate from the above was analysed for dissolved drug compound using a truncated UHPLC methodology. A Shimadzu Nexera X2 UHPLC system was used, with a reversed-phase column and a simple formic acid gradient elution. The UHPLC parameters are shown below:

Parameter	Value
**Mobile phase component A**	HPLC water plus 0.1% formic acid
**Mobile phase component B**	HPLC acetonitrile plus 0.1% formic acid
**Flow rate:**	0.6 ml/min
**Gradient program:**	Initial: 98% A, 2% B<br>At 1.2 mins: 2% A, 98% B<br>At 2.0 mins: 2% A, 98% B<br>Re-equilibration time: 0.6 min
**Autosampler temperature:**	25°C
**Column:**	Hypersil Gold, C_{18} 1.9 µm, 50 x 2.1 mm
**Column temperature:**	50°C
**Detector wavelength:**	254 nm
**Bandwidth:**	4 nm

A calibration solution was prepared in the following way: The same 10 mM solution used to prepare the solubility test sample was diluted in DMSO to give a 500 µM solution. This solution was then diluted with 50:50 acetonitrile:water to give a 50 µM solution. Aliquots (0.2, 2.0 and 5.0 µL) of this 50 µM solution were then injected onto the UHPLC system and the areas of the resultant peaks integrated to produce a calibration line. Aliquots of the test sample filtrate (0.4 and 5.0 µL) were then injected onto the UHPLC system and the resultant peak areas for any peaks corresponding to the test compound determined and quantified using the calibration line (the injection volume that gave a peak area closest to the calibrated range was used for determining solubility).

FaSSIF Solubility. This assay was conducted as previously described:

Brand, S.; Ko, E. J.; Viayna, E.; Thompson, S.; Spinks, D.; Thomas, M.; Sandberg, L.; Francisco, A. F.; Jayawardhana, S.; Smith, V. C.; Jansen, C.; De Rycker, M.; Thomas, J.; MacLean, L.; Osuna-Cabello, M.; Riley, J.; Scullion, P.; Stojanovski, L.; Simeons, F. R. C.; Epemolu, O.; Shishikura, Y.; Crouch, S. D.; Bakshi, T. S.; Nixon, C. J.; Reid, I. H.; Hill, A. P.; Underwood, T. Z.; Hindley, S. J.; Robinson, S. A.; Kelly, J. M.; Fiandor, J. M.; Wyatt, P. G.;

Marco, M.; Miles, T. J.; Read, K. D.; Gilbert, I. H. Discovery and Optimization of 5-Amino-1,2,3-triazole-4-carboxamide Series against Trypanosoma cruzi. **2017**, *60*, 7284-7299

Thomas, M. G., De Rycker, M., Ajakane, M., Albrecht, S., Álvarez-Pedraglio, A. I., Boesche, M., Brand, S., Campbell, L., Cantizani-Perez, J., Cleghorn, L. A. T., Copley, R. C. B., Crouch, S. D., Daugan, A., Drewes, G., Ferrer, S., Ghidelli-Disse, S., Gonzalez, S., Gresham, S. L., Hill, A. P., Hindley, S. J., Lowe, R. M., MacKenzie, C. J., MacLean, L., Manthri, S., Martin, F., Miguel-Siles, J., Nguyen, V. L., Norval, S., Osuna-Cabello, M., Woodland, A., Patterson, S., Pena, I., Quesada-Campos, M. T., Reid, I. H., Revill, C., Riley, J., Ruiz-Gomez, J. R., Shishikura, Y., Simeons, F. R. C., Smith, A., Smith, V. C., Spinks, D., Stojanovski, L., Thomas, J., Thompson, S., Underwood, T., Gray, D. W., Fiandor, J. M., Gilbert, I. H., Wyatt, P. G., Read, K. D.Miles, T. J. Identification of GSK3186899/DDD853651 as a Preclinical Development Candidate for the Treatment of Visceral Leishmaniasis. *J Med Chem* **2019**, *62*, 1180-1202

ChromLogD

Chrom LogD was measured as previously described:

Hill, A. P.; Young, R. J. Getting physical in drug discovery: a contemporary perspective on solubility and hydrophobicity. Drug Discov. Today **2010**, *15*, 648−655.

MDR1-MDCK Permeability assay

Bi-directional transport was assessed in the presence and absence of Pgp inhibitor GF120918 (Elacridar). MDCK-MDR1 cells (Netherlands Cancer Institute) were maintained in culture (DMEM, Gibco Cat: 61965-026 supplemented with 1% penicillin/streptomycin, 10% FCS) until required. For experimentation, cells were seeded onto individual transwell 'Thincerts' (Greiner, Cat 662610) at a density of 35,000 cells/well. Cells were grown at 37°C, 5% CO_2 for 3 days. On day 4, media was replaced with fresh media and incubated for 1 hour. Prior to experimentation, cells were incubated with Dulbeccos PBS (Gibco, 14287-080) with/without Pgp inhibitor, 2 μM GF120918, to ensure inactivation of the Pgp transporter. Cells were then dosed apically and basolaterally (1 μM test compound, 10 μM Lucifer Yellow (1% DMSO) with/without 2 μM GF120918) for 1 hour. 100 mL of solution from donor and receiver wells

was removed to a 96 well deepwell plate alongside 100 mL of dosing solution. 150 µL of acetonitrile containing internal standard (e.g., 100 ng/mL Sulfadimethoxine) was then added to all samples prior to analysis by LC-MS/MS. Amprenavir (positive control) were run alongside test compounds to confirm Pgp expression. To confirm monolayer integrity, a further 100 µL from each compartment was added to a 96 well F-bottomed microtitre plate containing a Lucifer Yellow standard curve for fluorescence determination of Lucifer Yellow concentrations. Papp (apparent permeability) values were calculated for each compound for both A to B and B to A directions in the presence and absence of Pgp inhibitor GF120918 and efflux ratios (ER) determined using the following equations:

$$\text{Papp (nm/sec)} = \frac{\text{(Volume receiver/ A) * (Response receiver/Response donor)}}{\text{Incubation time (seconds)}}$$

$$\text{Flux ratio} = \frac{\text{Abluminal-to-Luminal Papp (nm/sec) (A to B)}}{\text{Luminal-to-Abluminal Papp (nm/sec) (B to A)}}$$

***In vivo* pharmacokinetics**

All regulated procedures on living animals were carried out under the authority of a project license issued by the Home Office under the Animals (Scientific Procedures) Act 1986, as amended in 2012 (and in compliance with EU Directive EU/2010/63). Infected animals had access to food and water ad libitum and were housed under a 12-hour light/dark photoperiod. Animals used in these experiments were female NMRI and Balb/c mice (Harlan, UK).

Test compound DDD01008714 was dosed orally (P.O.) at 10 mg free base/kg (dose volume 10 mL/kg; dose vehicle: 10% (vol/vol) dimethyl sulfoxide (DMSO), 40% polyethylene glycol 400 (PEG400) and 50% deionized water) to female NMRI mice (n = 3) or intravenously (I.V.) at 3 mg free base/kg (dose volume 5 mL/kg; dose vehicle: 10% (vol/vol) dimethyl sulfoxide (DMSO) and 90% Saline) to female Balb/c mice (n = 3). Blood samples (10 µL) where taken from the tail vein of each mouse at 0.03, 0.08, 0.25, 0.5, 1, 2, 4, 6, 8, 24 hours post dose (0.03 and 24 hours post dose samples for I.V. leg only) and mixed with two volumes of deionized water (20 µL). After suitable sample preparation, the concentration of test compound in the whole blood from treated mice was determined by UPLC-MS/MS using a Quattro Premier XE (Waters, USA). Pharmacokinetic parameters were derived from the mean blood concentration time curve using PK solutions software v 2.0 (Summit Research Services, USA).

***In vivo* Mouse Efficacy Studies**.

The *T. cruzi* chronic efficacy study was run in Balb/c mice as previously described:

M.D. Lewis, A. Fortes Francisco, M.C. Taylor, J.M. Kelly, A new experimental

model for assessing drug efficacy against Trypanosoma cruzi infection based on

highly sensitive in vivo imaging, J. Biomol. Screen 20 (1) (2015) 36–43

Cryo-EM methods

The *Leishmania tarentolae* proteasome was prepared as described previously (Wyllie, S. *et al.*, 2019). The protein in 50 mM Tris pH 7.5, 150 mM NaCl, 5 mM $MgCl_2$, 4 mM ATP, 1 mM DTT was buffer exchanged to 50 mM Tris pH 7.5, 5 mM $MgCl_2$, 1 mM DTT. Protein samples used for grid preparation were prepared from two separate protein batches. One batch (Batch 1), previously frozen at -80°C, was passed through a GE Superose 3.2/300 column using the same final buffer prior to use. The protein samples were concentrated to 0.3-0.5 mg/ml using a Vivaspin 500 centrifugal concentrator MWCO 5 kDa. Samples were incubated with 20 µM compound taken from a 1 mM compound DMSO stock solution (Batch 1: 45 min at 37°C ; Batch 2: 1.5 hour at 20°C).

Quantifoil R 1.2/1.3 (Cu 300) with 2nm carbon grids were freshly glow discharged using a Pelco easiGlow at 25 mA for 1 min. A Vitrobot Mark IV (ThermoFisher) was used at 4°C and 100% humidity for double-sided blotting and plunging grids into liquid ethane. For Sample 1 (from protein Batch 1), 2.5 µl protein was applied to the grid, followed by 0.5 sec blotting, then a second 2.5 µl protein application was followed by 2.5 sec blotting. For Sample 2 (from protein Batch 2) a modified process using a triple protein application was used.

Two high resolution datasets were collected on a Titan Krios electron microscope equipped with a Falcon III direct electron detector at the Cambridge Pharmaceutical Cryo-EM Consortium, Nanoscience Centre, University of Cambridge. Data collection parameters are given in Supplementary Table 1. Dataset 1 from Sample 1 consisted of 1580 movies and Dataset 2 from Sample 2 consisted of 2713 movies. The particle density originally observed in Dataset 1 was low, which led to the collection of Dataset 2, having a much higher particle density. Dataset 1 contained mostly side view proteasome particles whilst Dataset 2 was heavily dominated by top view particles. After initially processing the two datasets individually, they were later merged together and re-processed as a single dataset. All data processing was performed using Relion 3.1 (Zivanov, J. *et al.*, 2020), initially carrying out motion correction, CTF estimation and autopicking (Laplacian of Gaussian). After particle extraction and 2D classification, template-based particle picking was run and after several rounds of 2D classification 683,992 particles were selected. A subset of these, consisting of 120,140 particles, were used to generate an initial model which was then used for 3D classification with the 683,992 particles. From the resulting 3D classes 109,642 particles were

selected and subjected to a further round of 3D classification with C2 symmetry, yielding 94,595 particles (12,548 from Dataset 1 and 82,047 from Dataset 2). Following 3D auto-refinement the indicated resolution was 3.49 Å. After generation of a mask and post-processing, the resolution had increased to 3.06 Å. A lengthy CTF refinement and particle polishing protocol was then run which yielded a final map resolution of 2.59 Å (Supplementary Figure 1). The final map required flipping to obtain the correct hand.

Model building was carried out using the CCP4 program suite (Winn, M. D. *et al.*, 2011). Initial coordinates for the proteasome model were obtained by running Molecular Replacement with Molrep (Vagin, A. & Teplyakov, A., 2010) using a high resolution unliganded CryoEM proteasome structure as a search model (unpublished). Model building was carried with Coot (Emsley, P. *et al.*, 2010) with ligand coordinate and dictionary files generated with Grade (Global Phasing Ltd). One half of the proteasome barrel was adjusted by rigid body fitting the individual protein chains to the map. The ligand was clearly identifiable in the density (Supplementary Figure 2). The other half of the model was generated using the C2 symmetry of the proteasome. The protein/ligand structure was refined using Refmac (Kovalevskiy, O. *et al.*, 2018) using restrained refinement and two-fold non-crystallographic symmetry restraints. Refinement details for the final model are given in Supplementary Table 2. The EM map and coordinates have been deposited in the Protein Data Bank (EMD-16963 and PDB 8OLU).

Supplementary Table 1. Microscope data collection parameters

**Hardware**	
Microscope	Krios D3512
Detector (mode)	Falcon III (Counting)
Accelerating voltage (kV)	300
Detector Pixel size (Å)	1.07

**Illumination parameters**	
Gun lens	5

Spot size	10
Illuminated area (μm)	1.38

**Dose**	
Nominal Magnification	75,000
Square pixel ($Å^2$)	1.07 x 1.07
Exposure time (s)	60
Number of Fractions	75
Total dose ($e^-/Å^2$)	30

**Apertures (μm)**	
C1	2000
C2	50
C3	2000
Objective	100

**EPU parameters**	
Defocus range (-μm)	-3.4, -3.2, -3.0, -2.8, -2.6, -2.4, -2.2, -2.0, -1.8
Autofocus frequency (μm)	10
Delay after stage shift (s)	5
Delay after image shift (s)	3.5
Exposures per hole	1

Supplementary Table 2. Model refinement

Refined resolution (Å)	2.59
R-factor	0.249
Average Fourier shell correlation	0.859
R.m.s. deviations	
Bond lengths (Å)	0.017
Bond angles (°)	2.13
Ramachandran plot	
Favoured (%)	95.4
Outliers (%)	0.6
Mean B-factors (Å^2)	
Protein atoms	28.3
Ligand atoms	38.3
MolProbity clash score	6.7
MolProbity score	2.3

Supplementary Figure 1. CryoEM data processing

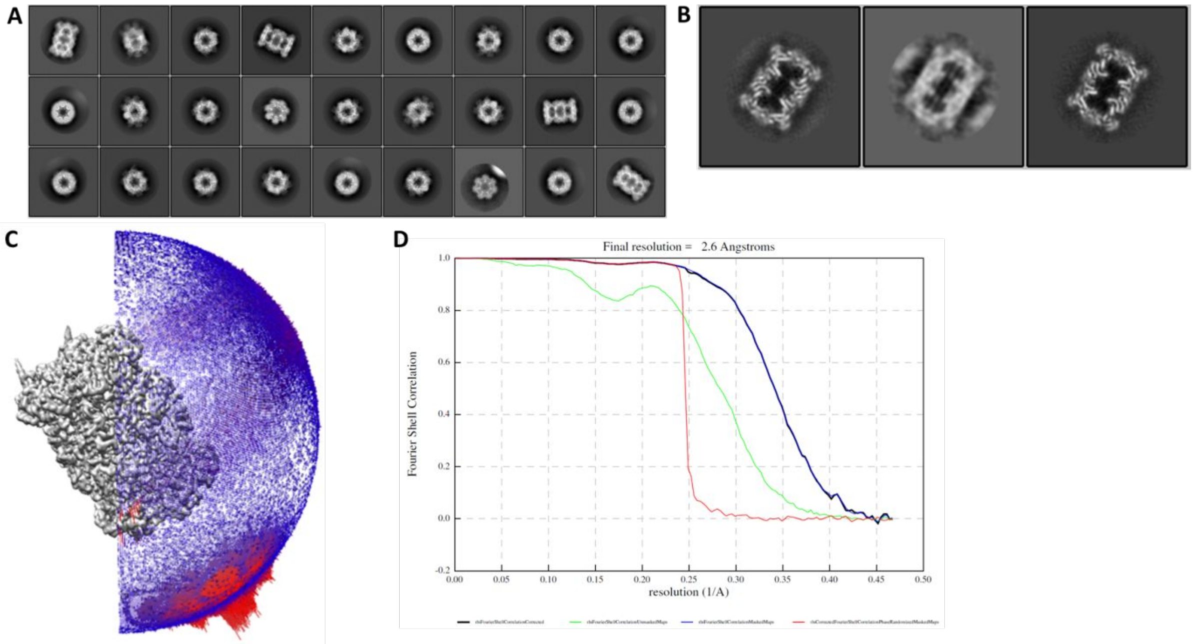

A) Final selected classes after 2D classification (683,992 particles); B) Final classes after 3D classification with C2 symmetry, classes 1 and 3 selected (94,595 particles selected out of 109,642); C) Angular distribution of particle views for final Refine3D map; D) Fourier shell correlation (FSC) plot.

Supplementary Figure 2. Final map with refined structure

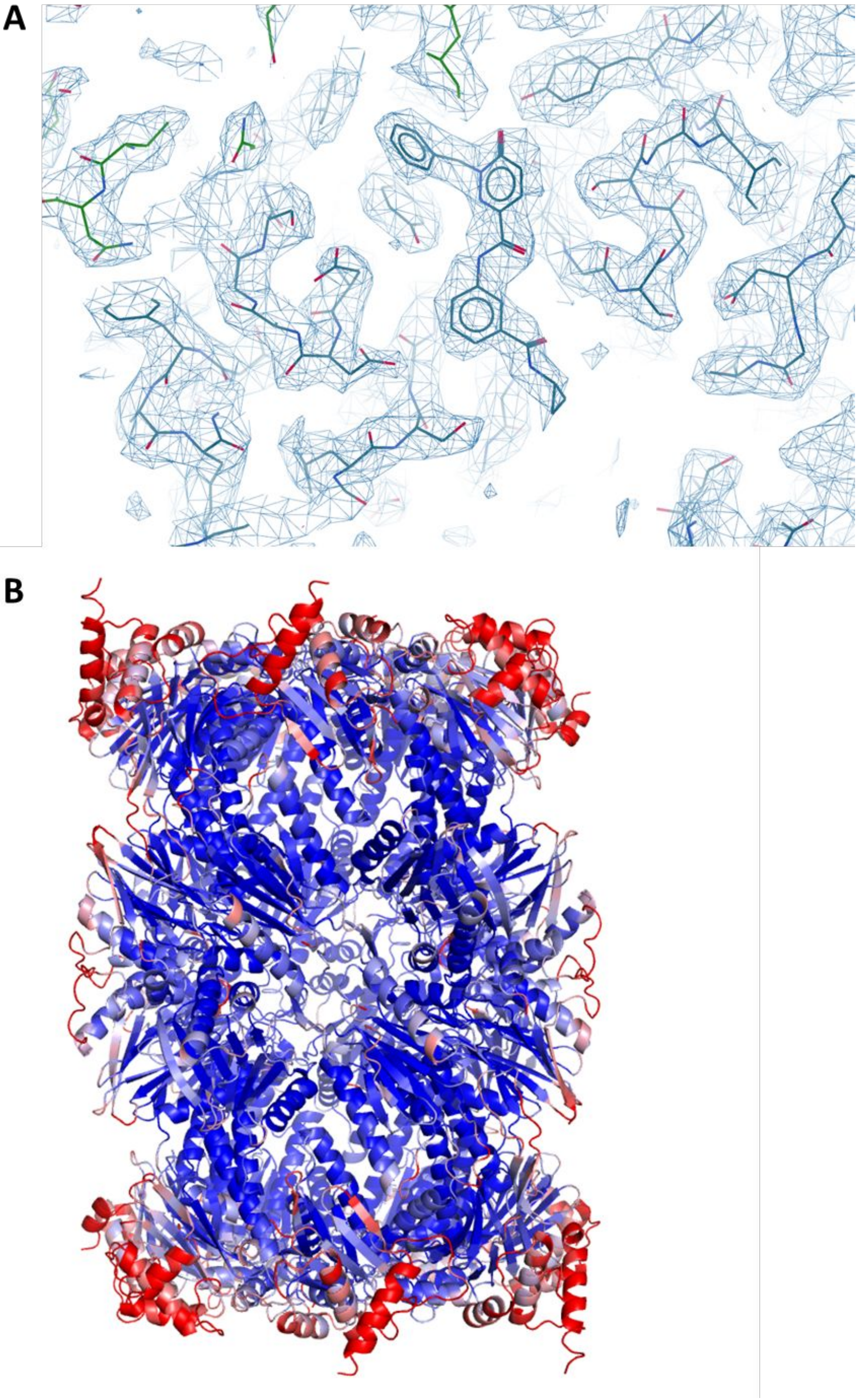

A) Electron potential map around ligand binding site contoured at 1.2σ; B. Refined structure coloured by B-factor (blue-lightblue-salmon-red) from 10 to 60 Å^2 (blue to red).

Cryo-EM methods references

Emsley, P.; Lohkamp, B.; Scott, W.G.; Cowtan, K.; Features and development of Coot, *Acta Cryst. D*, **2010**, *66*, 486 – 501

Kovalevskiy, O.; Nicholls, R.A. ; Long, F. ; Carlon, A. ; Murshudov, G.N.; Overview of refinement procedures within REFMAC5: utilizing data from different sources, *Acta Cryst. D*, **2018**, *74*, 215 – 227

Vagin, A.; Teplyakov, A.; Molecular replacement with MOLREP, *Acta Cryst. D*, **2010**, *66*, 22 – 25

Winn, M. D. ; Ballard, C.C. ; Cowtan, K.D. ; Dodson, E.J.; Emsley, P. ; Evans, P.R.; Keegan, R.M.; Krissinel, E.B.; Leslie, A.G.W.; McCoy, A. ; McNicholas, S.J. ; Murshudov, G.N. ; Pannu, N.S. ; Potterton, E.A. ; Powell, H.R. ; Read, R.J. ; Vagin, A. ; Wilson, K.S. ; Overview of the CCP4 suite and current developments, *Acta Cryst. D*, **2011**, *67*, 235 – 242

Wyllie, S.; Brand, S.; Thomas, M.; De Rycker, M.; Chung, C.W.; Pena, I.; Bingham, R. P.; Bueren-Calabuig, J. A.; Cantizani, J.; Cebrian, D.; Craggs, P. D.; Ferguson, L.; Goswami, P.; Hobrath, J.; Howe, J.; Jeacock, L.; Ko, E.-J.; Korczynska, J.; MacLean, L.; Manthri, S.; Martinez, M. S.; Mata-Cantero, L.; Moniz, S.; Nuhs, A.; Osuna Cabello, M.; Pinto, E.; Riley, J.; Robinson, S.; Rowland, P.; Simeons, F. R. C.; Shishikura, Y.; Spinks, D.; Stojanovski, L.; Thomas, J.; Thompson, S.; Viayna Gaza, E.; Wall, R. J.; Zuccotto, F.; Horn, D.; Ferguson, M. A. J.; Fairlamb, A. H.; Fiandor, J. M.; Martin, J.; Gray, D. W.; Miles, T. J.; Gilbert, I. H.; Read, K. D.; Marco, M.; Wyatt, P. G. Preclinical candidate for the treatment of visceral leishmaniasis that acts through proteasome inhibition. *Proc. Natl. Acad. Sci. U.S.A.* **2019**, 116, 9318−9323

Zivanov, J.; Nakane, T.; Scheres, S.H.W.; Estimation of high-order aberrations and anisotropic magnification from cryo-EM data sets in RELION-3.1, *IUCrJ*, **2020**, *7*, 253 – 267

Computational Methods

Homology modelling – Homology model of *T. cruzi* β4/β5 20S proteasome was generated using Maestro homology modelling tool (Schrödinger suites version 2020-04). Crystal structure of *L. tarentolae* β4/β5 20S proteasome in complex with **5** was used as template. The β4 and β5 subunits were first modelled separately then combined to form the whole proteasome complex. Multiple sequence alignments of the *T. cruzi* β4 and β5 subunits showed the sequence identities at 70% and 87%, respectively, to *L. tarentolae*. No big insertions were observed after alignment. The amino acid residues 5 Å from the ligand are almost identical between the two proteasomes, except Ser218 in *T. cruzi* in place of Thr218 in *L. tarentolae* proteasome. Both subunits were generated with **5** in place using energy-based modelling method without proximity constraints. All non-template residues were set to undergo minimisation and rotamers preserved. The combined *T. cruzi* proteasome **5** complexes were then minimised briefly with heavy atom convergence RMSD set to 0.3 Å.

Docking – Docking studies were performed using Glide (Schrödinger suites version 2020-04) with standard precision. Docking grid was generated using *T. cruzi* β4/β5 20S proteasome homology model generated in the previous section. 20 poses were sampled, and 10 poses were saved for analysis. Only poses reproducing the core ligand conformation of **5** *L. tarentolae* crystal complex were passed onto MP2-FMO calculations. All selected poses were merged with protein to form complexes and minimised briefly with heavy atom convergence RMSD set to 0.5 Å.

MP2-FMO Calculations – The binding site was selected as amino residues 5 Å from the ligand, and any connecting loops were expanded to reduce artefacts caused by truncation. Facio 23.1.5.64 was used for complex fragmentation and generation of FMO input files. MP2 theory and 6-31G* basic set were used for calculation in GAMESS (version 30).
